# Inverse relationship between neoantigen clonality and T-cell activity reveals distinct immune phenotypes in HNSCC

**DOI:** 10.1186/s12967-026-08371-z

**Published:** 2026-06-03

**Authors:** Babak Saravi, Daman Deep Singh, Lara Schorn, Julian Lommen, Felix Schrader, Max Wilkat, Andreas Vollmer, Michael Vollmer, Stefan Hartmann, Marius Hörner, Kaustubh Adhikari, Elisabeth Roider, Jakob Wollborn, Norbert Kübler, Christoph Sproll

**Affiliations:** 1 Department of Oral, Maxillofacial and Facial Plastic Surgery, Medical Faculty and University Hospital Düsseldorf, Heinrich-Heine-University Düsseldorf, 40225 Düsseldorf, Germany; 2 Department of Oral and Maxillofacial Plastic Surgery, University Hospital of Würzburg, 97070 Würzburg, Germany; 3 Department of Oral and Maxillofacial Surgery, Tübingen University Hospital, Osianderstrasse 2-8, 72076 Tuebingen, Germany; 4 School of Mathematics and Statistics, Faculty of Science, Technology, Engineering and Mathematics, The Open University, Milton Keynes, UK; 5 Department of Genetics, Evolution and Environment, and UCL Genetics Institute, University College London, London, UK; 6 Department of Dermatology, University Hospital of Basel, Basel, Switzerland; 7 Cutaneous Biology Research Center, Department of Dermatology, Massachusetts General Hospital, Harvard Medical School, Charlestown, USA; 8 Department of Anesthesiology, Brigham and Women’s Hospital, Mass General Brigham, Harvard Medical School, Boston, USA

**Keywords:** Head and neck squamous cell carcinoma, Neoantigen, Clonality, T-cell exhaustion, Tumour microenvironment, Immunotherapy, Immune checkpoint, Tumour mutational burden, TCGA, immunogenomics

## Abstract

**Background:**

Neoantigens derived from tumour-specific mutations are critical targets for T-cell recognition, yet the relationship between neoantigen clonality and immune response in head and neck squamous cell carcinoma (HNSCC) remains poorly understood. We hypothesised that clonal neoantigens, being present in all tumour cells, would drive T-cell exhaustion through persistent antigen exposure.

**Methods:**

We analysed 527 HNSCC tumours from The Cancer Genome Atlas. Neoantigens were predicted using pVACseq with MHCflurry binding prediction for patient-specific HLA class I alleles. We computed a Clonality Score (binder-weighted variant allele frequency normalised by neoantigen count) to quantify clonal origin independent of total burden. T-cell exhaustion was assessed using a 17-gene expression signature, alongside TIDE dysfunction scores and the Pan-Immune Score. Cox proportional hazards models with interaction terms tested whether prognostic effects of clonality depended on immune context.

**Results:**

Contrary to our hypothesis, neoantigen clonality showed strong inverse correlations with T-cell infiltration-associated signatures, including a 17-gene exhaustion/activation panel (ρ = −0.41, P = 9.8 × 10⁻²²), TIDE dysfunction (ρ = −0.53, P = 6.7 × 10⁻³⁸), and Pan-Immune Score (ρ = −0.50, P = 8.5 × 10⁻³³). Tumours stratified into four phenotypes: Hot/Low Clonality (33%) exhibited high immune infiltration and cytolytic activity; Cold/High Clonality (33%) showed minimal immune engagement despite abundant clonal neoantigens. High clonality also associated with reduced antigen presentation machinery expression (ρ = −0.31, P = 1.0 × 10⁻¹²), suggesting impaired antigen visibility as a potential upstream mechanism. Survival analysis revealed a significant clonality × immune status interaction (P = 0.034). In ‘hot’ tumours, high clonality predicted improved survival (HR = 0.72, P = 0.043), whereas clonality had no prognostic effect in ‘cold’ tumours (HR = 1.00, *P* = 0.98).

**Conclusions:**

Neoantigen clonality inversely predicts T-cell activity in HNSCC, identifying high-clonality tumours as immunologically ‘cold’ despite abundant tumour-specific antigens. Crucially, the prognostic benefit of clonality is realised only when T-cell effectors are present, reconciling our findings with the paradigm that clonal neoantigens represent optimal immunological targets. This context-dependent relationship generates the hypothesis that neoantigen clonality, when interpreted alongside immune context, may help identify patients who could benefit from immune-priming strategies prior to checkpoint inhibitor therapy, pending validation in immunotherapy-treated cohorts.

**Supplementary Information:**

The online version contains supplementary material available at 10.1186/s12967-026-08371-z.

## Introduction

Head and neck squamous cell carcinoma (HNSCC) represents a major global health burden, ranking as the sixth most common malignancy worldwide with approximately 890,000 new cases and 450,000 deaths annually [1, 2]. Despite advances in multimodal therapy combining surgery, radiotherapy, and chemotherapy, five-year survival remains stagnant at 40–50%, with nearly one-third of patients experiencing disease recurrence [3, 4]. The approval of immune checkpoint inhibitors (ICIs) targeting the PD-1/PD-L1 axis has transformed the therapeutic landscape for recurrent or metastatic HNSCC, yet objective response rates remain limited to 15–20% of patients [5, 6]. This therapeutic plateau underscores the urgent need to identify molecular determinants of immune responsiveness and resistance.

Tumour neoantigens—peptides derived from somatic mutations presented on major histocompatibility complex (MHC) molecules—have emerged as critical targets for T-cell-mediated antitumour immunity [7, 8]. The seminal work by McGranahan and colleagues demonstrated that clonal neoantigens, present in all tumour cells, elicit robust T-cell responses and predict sensitivity to checkpoint blockade in lung cancer and melanoma [9]. This paradigm posits that clonal neoantigens represent optimal immunotherapeutic targets because T cells recognising these antigens can theoretically eliminate the entire tumour mass, whereas responses against subclonal neoantigens target only tumour subpopulations [10, 11]. Consequently, neoantigen clonality has been incorporated into predictive frameworks for immunotherapy response, with high clonal neoantigen burden associated with improved survival outcomes [12, 13].

However, the relationship between neoantigen clonality and immune activity in the tumour microenvironment remains incompletely understood. Chronic antigen exposure drives T-cell exhaustion—a dysfunctional state characterised by progressive loss of effector function, sustained expression of inhibitory receptors (PD-1, TIM-3, LAG-3, CTLA-4), and distinct transcriptional programmes [14, 15]. This exhaustion paradigm suggests that clonal neoantigens, by virtue of their ubiquitous expression across tumour cells, should subject infiltrating T cells to persistent antigenic stimulation and thereby promote exhaustion [16]. Yet empirical evidence directly linking neoantigen clonality to T-cell exhaustion phenotypes in human tumours is lacking. Importantly, exhaustion-related transcriptional signatures derived from bulk RNA sequencing reflect a composite of immune cell abundance and functional state, complicating the distinction between reduced T-cell presence and altered exhaustion programmes in treatment-naïve tumours.

HNSCC presents a unique model to interrogate this relationship. The disease encompasses both HPV-positive and HPV-negative aetiologies with distinct immunological profiles, exhibits substantial heterogeneity in tumour-infiltrating lymphocyte abundance, and demonstrates variable responses to checkpoint inhibition [17, 18]. Moreover, comprehensive molecular characterisation through The Cancer Genome Atlas (TCGA) provides an unprecedented opportunity to integrate genomic, transcriptomic, and clinical data across large patient cohorts [19].

Here, we systematically investigated the relationship between neoantigen clonality and T-cell activity in HNSCC tumours. We hypothesised that high neoantigen clonality would associate with increased T-cell exhaustion through persistent antigen exposure. Contrary to this expectation, we discovered a paradoxical inverse relationship: tumours with high neoantigen clonality exhibited reduced immune infiltration, diminished immune-related transcriptional activity, and attenuated antigen presentation—a pattern more consistent with immune ignorance than with persistent antigen-driven exhaustion. Importantly, we demonstrate that the prognostic value of neoantigen clonality depends critically on immune context, with high clonality conferring survival benefit only in immunologically ‘hot’ tumours harbouring T-cell infiltration. These findings challenge prevailing assumptions about clonal neoantigens and immune engagement, reveal distinct immunophenotypes defined by the intersection of clonality and immune status, and identify neoantigen clonality as a context-dependent biomarker for patient stratification in immunotherapy.

## Materials and methods

### Data and study cohort

Somatic mutation data for head and neck squamous cell carcinoma (HNSCC) were obtained from The Cancer Genome Atlas (TCGA). We downloaded whole-exome sequencing variant calls for 527 HNSCC tumors from the TCGA Genomic Data Commons. High-confidence HLA class I genotypes for each patient were obtained using the method from Li et al., which combines multiple in silico HLA typing algorithms [20]. Briefly, this consensus approach integrated results from eight HLA callers (including POLYSOLVER, OptiType, xHLA, HLA-HD, hla-genotyper, SOAP-HLA, HLA-VBSeq, and Kourami) to establish reliable HLA-A, HLA-B, and HLA-C alleles per individual [20]. HLA loss of heterozygosity (LOH) events were identified using the LOHHLA algorithm [21]: for each tumor, HLA alleles were classified as lost or retained based on allele-specific copy-number analysis of the HLA locus. We annotated any sample with at least one lost HLA allele as HLA LOH-positive.

Two immune-related variables were derived from standardized TCGA pipelines: tumor mutational burden (TMB) and cytolytic activity (CYT). TMB was calculated by enumerating all non-synonymous somatic mutations detected in coding regions of the exome and dividing this count by the total megabases of coding sequence covered, following the convention established for TCGA studies [20]. Variants considered non-synonymous encompassed missense substitutions, frameshift and in-frame indels, stop-gain or stop-loss changes, splice-site–proximal alterations, and other protein-altering consequences, as defined through sequence ontology annotation. The cytolytic activity score (CYT) was derived from tumor RNA-seq expression profiles as a quantitative marker of immune effector function within the tumor microenvironment. Cytolytic signaling in cytotoxic T lymphocytes and natural killer cells is largely mediated by coordinated release of granzyme A (GZMA) and perforin (PRF1); thus, their expression patterns in bulk tumor tissue serve as surrogate indicators of intratumoral cytotoxic potential. Consistent with prior immunogenomic analyses, CYT was defined as the geometric mean of GZMA and PRF1 expression (transcripts per million, with a small offset to stabilize low-expression values) [20]. This metric captures the overall intensity of endogenous cytotoxic immune activity in each tumor.

We further incorporated immune and expression profiles from TCGA Pan-Cancer Atlas resources [22]. To characterize immune cell infiltration, we used CIBERSORT, a gene-expression deconvolution method that infers the composition of leukocyte subsets from bulk RNA-Seq data [23]. Pre-computed CIBERSORT estimates of 22 immune cell type fractions for TCGA HNSCC tumors were obtained and merged into our dataset. For gene expression, we retrieved the TCGA Pan-Cancer normalized RNA-Seq expression matrix (FPKM values); from this we extracted each tumor’s expression of the immune checkpoint ligand PD-L1 (*CD274* gene). Tumor Immune Dysfunction and Exclusion (TIDE) scores were calculated by applying the TIDE algorithm to the HNSCC RNA-Seq data [24]. TIDE generates two metrics per sample -- a T cell dysfunction score and a T cell exclusion score [24]. The dysfunction component is defined and most interpretable in immune-infiltrated tumours; in the present HNSCC cohort, it correlates strongly with immune infiltration-associated signatures (ρ = 0.79 with Pan-Immune Score), and we therefore use it as a continuous proxy for immune-active (‘hot’) context in stratified analyses. The exclusion score reflects factors preventing T-cell entry in non-inflamed tumours. In addition, clinical and demographic data (including patient age, sex, race, clinical stage, and survival follow-up) were obtained from TCGA. HPV status for TCGA HNSCC tumors was obtained from the PanImmune resource, which provides harmonized viral detection calls across TCGA cancers [22]. Briefly, the PanImmune pipeline screens tumor RNA-seq libraries against curated reference sets for oncogenic viruses, including human papillomavirus, using a k-mer–based classifier derived from BioBloom Tools. For each tumor, the method quantifies HPV-specific sequencing reads and normalizes this count to the total library depth to generate a normalized reads-per-million (NRPM) value. Samples exceeding the predefined HPV detection threshold (NRPM ≥ 10) are designated HPV-positive, whereas tumors below this threshold are considered HPV-negative. These assignments are based purely on viral transcript evidence and were cross-checked for consistency with available clinical annotations. All datasets were linked by the TCGA sample barcodes.

### Somatic mutation processing and annotation

We processed TCGA somatic mutation calls to enable downstream neoantigen prediction. The TCGA mutation data were provided in MAF (Mutation Annotation Format); we converted each MAF to a VCF (Variant Call Format) using the vcf2maf/maf2vcf conversion utility (MSKCC). Converting to VCF ensured standardized representation of indels and facilitated use of variant annotation tools. We then annotated all somatic variants using the Ensembl Variant Effect Predictor (VEP) [25]. VEP (release 110.1) was run in offline mode with the GRCh37/hg19 reference genome, incorporating the corresponding Ensembl gene models and variant consequences. We enabled VEP options to report gene symbols, sequence ontology terms, transcript support level, and HGVS nucleotide/protein changes. Importantly, we also integrated the “Wildtype” and “Frameshift” peptide sequence plugins from the pVACtools suite into VEP. These plugins generated the mutant peptide sequences (and corresponding wild-type peptides for frameshifts) for each coding variant, which are required for neoantigen prediction. The annotated output for each sample was a VCF file containing all somatic variants with their functional annotations and predicted peptide sequences.

### Neoepitope prediction with pVACseq

Predicted tumor neoantigens (mutant peptide epitopes) were identified using pVACseq, part of the pVACtools immunogenomics toolkit [26]. For each tumor, we compiled the patient’s HLA class I genotype (HLA-A, -B, and -C alleles) from the high-confidence calls described above. Alleles were formatted at six-digit resolution with the standard “HLA-” prefix (e.g., HLA-A*02:01, HLA-B*15:01). We then ran pVACseq (v6.0) on the VEP-annotated VCFs to predict peptide binders to the patient’s HLA alleles. We selected the MHCflurry algorithm (v2.1.5) as our binding affinity predictor, which uses allele-specific neural networks trained on peptide–MHC binding data [27]. pVACseq was configured to evaluate 8–11 amino acid peptides spanning each missense mutation or indel. Only variants passing quality filters in the VCF (PASS) were considered. For each candidate peptide, pVACseq calculated the predicted MHC class I binding affinity (IC50 in nanomolar) and percentile rank, among other metrics. The pVACseq pipeline output, for each sample, a comprehensive list of predicted neoepitopes binding affinities and supporting data, as well as summary counts of neoantigens per tumor.

### Neoantigen feature summarization

We aggregated the data to derive various neoantigen metrics per tumor. Predicted peptides were categorized by binding affinity: those with IC50 ≤ 500 nM were considered “binders”, and a subset with IC50 ≤ 50 nM were considered “strong binders.” For each tumor we counted the total number of predicted class I neoepitopes (all peptides evaluated), the number of binders (≤ 500 nM), and the number of strong binders (≤ 50 nM). These counts were also tallied by peptide length (8, 9, 10, and 11 amino acids) to assess the length distribution of neoantigens. For each tumour, binder-weighted VAF was calculated as the sum of variant allele frequencies across all predicted binder peptides (IC50 ≤ 500 nM). Because multiple peptides may derive from a single mutation, this metric weights mutations by both their VAF and their peptide yield [23]. To quantify neoantigen clonality independent of overall burden, we defined a Clonality Score as the binder-weighted variant allele frequency (VAF) divided by the number of binding neoantigens plus one:

$$\begin{gathered}{\text{Binder - weighted VAF }} = {\Sigma _i}(VA{F_i} \times {\text{ }}{I_i}),{\text{ }} \hfill \\ where{\text{ }}{I_i} = {\text{ }}1{\text{ }}if{\text{ }}IC50{\text{ }} \leqslant {\text{ }}500{\text{ }}nM \hfill \\ \end{gathered} $$

$$\begin{gathered}{\text{Clonality Score = }} \hfill \\{\text{Binder - weighted VAF /}} \hfill \\{\text{ }}\left( {neo\_n\_500{\text{ }} + {\text{ }}1} \right) \hfill \\ \end{gathered} $$

This metric captures the average clonality of neoantigens while normalizing for neoantigen count. Higher Clonality Scores indicate tumours whose neoantigens derive predominantly from clonal (high-VAF) mutations, whereas lower scores indicate subclonal origin. The denominator includes a pseudocount of 1 to avoid undefined values for tumours with zero predicted neoantigens.

The Clonality Score is best interpreted as a variant allele frequency (VAF)-based proxy for neoantigen clonality rather than a direct measurement of cancer cell fraction (CCF). VAF is influenced by tumour purity, ploidy, and local copy number state, and does not equate to true clonal versus subclonal architecture as estimated by tools such as PyClone or ABSOLUTE. Per-variant CCF estimation via PyClone requires allele-specific read counts at each variant locus, which necessitates re-processing of raw sequencing data (BAM files) and was not feasible at the scale of the full TCGA HNSCC cohort (*n* = 527). To validate the Clonality Score as a proxy for tumour clonal architecture, we compared it against ABSOLUTE-estimated Subclonal Genome Fraction (SGF), a copy-number-based clonality metric derived from allele-specific copy number analysis that is independent of our VAF-based approach (see Results; Supplementary Table S9; Supplementary Figure S2A). We also performed sensitivity analyses using purity-adjusted Clonality Scores and alternative metric formulations to assess robustness (Supplementary Table S12). An interactive web-based calculator for computing the Clonality Score and classifying tumour immune phenotypes is freely available at https://sciora.me/tools/clonality-score.

We emphasise that the Clonality Score should be interpreted as a VAF-weighted neoantigen clonality proxy rather than a direct measure of true tumour clonal architecture. Throughout this manuscript, references to ‘clonality’ in the context of this metric should be understood as a relative ranking of tumours along a VAF-weighted spectrum, in which higher scores reflect a greater contribution of high-VAF (typically more clonally distributed) variants to the predicted neoantigen pool. This interpretation is supported by the orthogonal validation against ABSOLUTE-estimated Subclonal Genome Fraction (rho = − 0.262, *P* = 3.95 × 10⁻⁹; Supplementary Table S9), which independently reproduced the central immune associations using a copy-number-based clonality metric not influenced by VAF or peptide yield.

To assess public vs. private neoantigens, we compared the peptide sequences across the cohort. Any neoepitope that was identically predicted in at least two different patients was labeled a “public” neoantigen (shared), whereas neoepitopes unique to a single patient were labeled “private.” For each tumor, we counted the number of public neoantigens and the number of private neoantigens it contained. Similarly, at the HLA allele level, we counted how many peptides restricted to a given allele were shared between patients versus private to one patient. This analysis highlights whether tumors tended to generate convergent (“public”) neoantigen peptides or largely distinct personal neoantigens.

Finally, we aggregated neoantigen data at the gene level. For each mutated gene across the cohort, we computed summary statistics such as: (i) the number of tumors in which the gene produced at least one predicted neoantigen, (ii) the median number of binder neoepitopes per tumor (neo_n_500) arising from that gene, (iii) the fraction of those neoepitopes that were strong binders, and (iv) the median TIDE dysfunction score, TIDE exclusion score, and PD-L1 expression in tumors harboring neoantigens from that gene. This allowed us to rank genes by their overall neoantigen contribution and examine associations with immune context (e.g. whether tumors with neoantigens from a particular gene tend to have high T-cell dysfunction or high PD-L1).

### T-cell exhaustion, antigen presentation, and immune score analyses

Throughout this manuscript, we use the following terminological conventions: “immune infiltration” refers to the overall abundance of immune cells inferred from bulk RNA-seq signatures (e.g., Pan-Immune Score, CIBERSORT estimates); “T-cell activity” encompasses both T-cell infiltration and effector function as captured by cytolytic activity (CYT) and TIDE dysfunction scores; and “exhaustion signatures” denotes the transcriptional expression of canonical exhaustion-associated genes (e.g., PDCD1, HAVCR2, CTLA4), which in bulk data reflect a combination of T-cell abundance and functional state. We recognise that bulk RNA-seq cannot fully disentangle these components. To characterize T-cell dysfunction in the tumour microenvironment, we extracted TCGA HNSCC RNA-sequencing expression data (STAR-aligned TPM values). We compiled a panel of 17 canonical T-cell exhaustion-associated genes based on established literature [14]: core exhaustion markers (*PDCD1*/PD-1, *LAG3*, *HAVCR2*/TIM-3, *CTLA4*, *TIGIT*, *ENTPD1*/CD39), exhaustion-associated transcription factors (*TOX*, *TCF7*/TCF-1, *TBX21*/T-bet, *EOMES*, *PRDM1*/Blimp-1, *BATF*, *IRF4*, *NR4A1*), and co-inhibitory receptors (*CD244*/2B4, *CD160*, *BTLA*). A composite Exhaustion Score was calculated as the geometric mean of all 17 gene expression values, with a pseudocount of 0.01 added to TPM values to avoid undefined logarithms. A Core Exhaustion Score was computed using only the six primary checkpoint/exhaustion markers (*PDCD1*, *LAG3*, *HAVCR2*, *CTLA4*, *TIGIT*, *TOX*) for sensitivity analyses.

To assess antigen presentation capacity, we quantified expression of 17 genes involved in MHC class I and class II antigen presentation pathways. The panel included: MHC class I heavy chains (*HLA-A*, *HLA-B*, *HLA-C*), beta-2-microglobulin (*B2M*), peptide transporter subunits (*TAP1*, *TAP2*), tapasin (*TAPBP*), endoplasmic reticulum chaperones (*CALR*/calreticulin, *PDIA3*/ERp57, *CANX*/calnexin), immunoproteasome subunits (*PSMB8*/LMP7, *PSMB9*/LMP2, *PSMB10*/MECL-1), and MHC class II pathway components (*HLA-DRA*, *HLA-DRB1*, *CD74*/invariant chain, *CIITA*). A composite Antigen Presentation Score was calculated as the geometric mean of all gene expression values. A separate MHC Class I Score was computed using only *HLA-A*, *HLA-B*, *HLA-C*, *B2M*, *TAP1*, and *TAP2* to assess classical antigen presentation capacity independent of professional antigen-presenting cell infiltration.

We assessed tumour-associated immunosuppression using a curated panel of 14 immunosuppressive genes derived from Budhwani et al. and established literature [28]. The panel included: adenosine pathway genes (*NT5E*/CD73), TWEAK receptor (*TNFRSF12A*), TGF-β pathway components (*TGFB1*, *TGFB2*, *TGFBR1*, *TGFBR2*), pro-inflammatory cytokines associated with immunosuppression (*IL1A*, *IL1B*), indoleamine 2,3-dioxygenase enzymes (*IDO1*, *IDO2*), regulatory T-cell markers (*FOXP3*, *IL10*, *IL10RA*), and angiogenic factors (*VEGFA*). The Immunosuppressive Gene Score was calculated as the geometric mean of expression values across all genes.

To validate our findings against established immune phenotyping frameworks, we integrated external immune scores from Budhwani et al. [28]. The Pan-Immune Score, originally developed using weighted gene co-expression network analysis (WGCNA) on TCGA cervical squamous cell carcinoma data and validated in TCGA HNSCC, represents overall immune activity based on 448 survival-associated immune genes. The Immune Suppression Score captures expression of 20 immune-suppressive genes (including checkpoint inhibitors, Treg-associated genes, and soluble mediators) for which high expression paradoxically associated with improved survival probability. We also obtained Immunophenoscore (IPS4) values, which integrate effector cells, suppressive cells, MHC-related molecules, and immunomodulators with positive weighting for CTLA-4/PD-1/PD-L1/PD-L2 expression [29]. Score values for TCGA HNSCC samples were obtained from Budhwani et al. [28] and merged with our dataset by TCGA patient barcode.

### Immune phenotype classification

To stratify tumours into discrete immune phenotypes, we performed median dichotomization of the Clonality Score and Pan-Immune Score. Tumours were classified into four mutually exclusive phenotypes: (1) Hot/Low Clonality: Pan-Immune Score ≥ median AND Clonality Score < median; (2) Hot/High Clonality: Pan-Immune Score ≥ median AND Clonality Score ≥ median; (3) Cold/Low Clonality: Pan-Immune Score < median AND Clonality Score < median; (4) Cold/High Clonality: Pan-Immune Score < median AND Clonality Score ≥ median. This classification generates groups reflecting the intersection of immune activity status (“hot” vs. “cold”) and neoantigen clonality.

### Data integration and quality control

All sample-level data were merged into a single analytical table keyed by the TCGA sample identifier. To ensure consistent matching, we truncated TCGA sample codes to the 12-character patient barcode (e.g. TCGA-AB-1234) before merging, since some data sources listed only the patient-level code. Numeric variables obtained from external files (e.g. TMB values, expression levels, cell fractions) were converted to numeric data types in R/Python and checked for out-of-range or impossible values. Missing data from any source were allowed to propagate and were encoded as NA (not available) in the merged dataset without imputation. The final master dataset included: clinical and demographic variables, TMB and CYT scores, TIDE dysfunction and exclusion scores, CIBERSORT immune cell fractions, PD-L1 expression, HLA LOH status, HLA genotype (for each class I allele), and all computed neoantigen metrics, including counts of total/strong binders, binder-weighted VAF, and public/private counts. We also added a few derived fields such as the neoantigen count normalized by TMB (neoantigens per mutation rate) and the gene-level neoantigen summary metrics described above. These comprehensive data served as the basis for downstream statistical analyses.

Of the 527 tumours in the full cohort, 457 had complete data for survival analysis (overall survival time > 0, Clonality Score, TIDE dysfunction score, and HPV status). Exclusions comprised 45 patients with missing survival time (no recorded date of death or last follow-up), 19 patients lacking a Clonality Score (due to absence of predicted neoantigen binders), and 6 patients missing TIDE dysfunction scores. Comparison of included versus excluded patients revealed no significant differences in age (median 61 vs. 59 years, *P* = 0.237), TMB (median 2.75 vs. 2.78 mut/Mb, *P* = 0.998), Clonality Score (median 0.23 vs. 0.22, *P* = 0.588), or HPV status distribution (chi-squared *P* = 0.702), suggesting that missingness was non-informative (Supplementary Table S14).

### Statistical analyses

All statistical analyses were performed using R (version 4.3.0) and Python (version 3.10). Data wrangling and summary computations made heavy use of the R packages *dplyr*, *data.table*, and *stringr*. Visualizations and clustering were generated with *ggplot2* and *pheatmap*, among other libraries. For group comparisons of continuous variables (e.g. neoantigen counts between subgroups), we used non-parametric tests: the Mann–Whitney U test (two groups) or Kruskal–Wallis test (for multi-group comparisons). Correlations between quantitative features (for example, between neoantigen counts and immune scores) were assessed using Spearman’s rank correlation (ρ), given the non-normal distributions of many variables. Where multiple comparisons were performed, we applied the Benjamini–Hochberg procedure to control the false discovery rate (FDR) and reported adjusted *q*-values as appropriate. Survival analyses were conducted using the Kaplan–Meier method and log-rank tests to compare groups (e.g. patients with high vs. low neoantigen metrics), and we built multivariable Cox proportional hazards models to evaluate the independent effect of neoantigen features on overall survival. The Cox models included covariates such as patient age and clinical stage to adjust for potential confounders. To test whether the prognostic effect of neoantigen clonality depended on immune context, we included a Clonality Score × TIDE dysfunction interaction term in an extended Cox model. Subsequently, we performed stratified analyses in ‘hot’ (TIDE dysfunction ≥ median) and ‘cold’ (TIDE dysfunction < median) tumour subgroups to characterise the effect of clonality within each immune context. Differences in immune parameters across phenotypes were assessed using Kruskal-Wallis tests, with post-hoc Dunn’s tests for pairwise comparisons. Association between HPV status and phenotype distribution was evaluated using chi-squared tests. Given the distinct biology of HPV-positive versus HPV-negative HNSCC, all primary correlation analyses were repeated within HPV strata to assess consistency of findings. Spearman correlations between clonality and immune variables were computed separately for HPV-positive and HPV-negative subgroups. Correlation coefficients were compared between strata using Fisher’s z-transformation to test for significant differences. To evaluate whether observed correlations between clonality and immune variables were confounded by overall immune infiltration, we computed partial Spearman correlations controlling separately for Pan-Immune Score, CD8 T-cell fraction (CIBERSORT), and total lymphocyte fraction. Partial correlations were obtained by rank-transforming both variables and the covariate, regressing each on the covariate, and correlating the residuals. To further quantify the independent contribution of clonality to exhaustion after accounting for immune context and mutational burden, we fitted ordinary least-squares regression models of the form: Exhaustion Score ~ Clonality Score + Pan-Immune Score + TMB. All variables were entered untransformed. To assess robustness to the specific clonality metric employed, we repeated all core correlation analyses using four alternative clonality definitions: raw binder-weighted VAF, mean VAF per binder, binder-weighted VAF normalised by total neoantigen count (rather than binder count), and log-transformed Clonality Score. Stratified analyses within immune-hot (Pan-Immune Score ≥ median) and immune-cold (Pan-Immune Score < median) subgroups tested whether clonality–immune associations persisted after restricting to tumours with comparable immune activity. Mann-Whitney U tests compared continuous immune variables between HPV groups. All statistical tests were two-sided unless otherwise specified, and a significance threshold of *P* < 0.05 (or *q* < 0.05 after FDR correction) was used.

To address potential confounding by human papillomavirus (HPV) status in survival analyses, all Cox proportional hazards models were repeated with HPV status (positive vs. negative) as an additional covariate. Specifically, we fitted four models: (i) Clonality Score alone; (ii) Clonality Score + HPV status; (iii) Clonality Score + HPV status + TMB; and (iv) an interaction model including Clonality Score, immune status (hot/cold), their interaction term, and HPV status. Stratified analyses within hot and cold tumour subgroups additionally included HPV status as a covariate. All continuous variables were z-standardised for Cox modelling (Supplementary Table S11; Supplementary Figure S2D).

To validate the four-phenotype classification derived from median dichotomization, we performed unsupervised clustering analyses. K-means clustering (k = 2 to 5) was applied to z-standardised Clonality Score and TIDE dysfunction values, with optimal k assessed by silhouette analysis. Gaussian Mixture Models (GMM; k = 2 to 5) were fitted with the Bayesian Information Criterion (BIC) used for model selection. Concordance between unsupervised clusters and median-split phenotypes was assessed using the Adjusted Rand Index (ARI). Additionally, tertile-based stratification was performed as a sensitivity analysis to test robustness to the choice of dichotomization threshold (Supplementary Table S13; Supplementary Figure S2C).

To examine whether immune exclusion programmes contribute to the clonality-immune disconnect, we correlated the Clonality Score with expression of genes involved in interferon-gamma (IFN-gamma) signalling (IFNG, STAT1, IRF1, JAK1, JAK2, CXCL9, CXCL10, CXCL11, IFNGR1, IFNGR2), WNT/beta-catenin signalling (CTNNB1, WNT5A), PTEN/PI3K pathway (PTEN), and MAPK pathway (BRAF, KRAS, MAP2K1). A composite IFN-gamma Score was calculated as the geometric mean of all ten IFN-gamma pathway gene expression values. AXL was additionally examined as a marker of epithelial-mesenchymal transition (EMT)-associated immune exclusion. Gene expression data were obtained from the TCGA PanCancer Atlas RNA-Seq expression matrix. Partial Spearman correlations controlling for ABSOLUTE tumour purity were computed to assess whether associations were independent of cellularity (Supplementary Tables S10, S10b; Supplementary Figure S3).

To independently validate the hot/cold immune phenotype classification, we evaluated whether alternative immune-context measures yielded concordant tumour assignments. We computed an ESTIMATE-style composite immune score (geometric mean of CD8A, CD3D, CD3E, GZMB, GNLY, NKG7, IL2RB, PDCD1, CTLA4, IFNG, STAT1, CXCL9, CXCL10), and additionally evaluated single-gene T-cell markers (CD3D, GZMB, GNLY, NKG7), CIBERSORT-derived CD8 T-cell fractions, the IFN-γ Composite Score, the LIexpression score from Wolf et al., and the Immunologic Constant of Rejection (ICR) score. Hot/cold classifications based on each alternative score were compared against the primary TIDE-dysfunction-based classification using percent concordance and Cohen’s kappa (Supplementary Table S15; Supplementary Figure S5). To independently confirm the four-phenotype survival differences and identify which specific group contrasts drive the global log-rank signal, we performed pairwise log-rank tests between all six pairs of immune phenotypes with Benjamini–Hochberg false discovery rate (FDR) correction and Bonferroni adjustment. We additionally fitted a multivariable Cox proportional hazards model treating the four-phenotype variable as a single categorical predictor (Hot/Low Clonality as the reference category), adjusting for age at diagnosis, advanced clinical stage, and HPV status (Supplementary Table S17; Supplementary Figure S6).

## Results

### Cohort characteristics and quality control

We analysed 527 head and neck squamous cell carcinoma (HNSCC) tumours (Table 1). The cohort comprised 385 males (73.1%) and 142 females (26.9%). Most patients self-identified as white (451/527, 85.6%), with additional representation from Black or African American (48/527, 9.1%), Asian (11/527, 2.1%), and American Indian or Alaska Native (2/527, 0.4%) populations; race information was missing for 15 patients (2.8%). Clinical stage at diagnosis was predominantly advanced—Stage IVA in 204/527 (38.7%)—with Stage III in 96/527 (18.2%), Stage II in 83/527 (15.7%), Stage I in 19/527 (3.6%), and Stage IVB/IVC in 10/527 (1.9%) and 6/527 (1.1%), respectively; clinical stage was missing for 115/527 (21.8%). Pathologic stage showed a similar distribution (Stage IVA in 199/527, 37.8%); data were missing for 155/527 (29.4%). Tumour grade was most frequently G2 (247/527, 46.9%), followed by G3 (99/527, 18.8%), G1 (51/527, 9.7%), G4 (5/527, 0.9%) and GX (17/527, 3.2%); grade was missing for 108/527 (20.5%). HPV status was positive in 72/527 (13.7%) and negative in 448/527 (85.0%); seven cases (1.3%) were missing. At last follow-up, 303/527 (57.5%) were alive and 224/527 (42.5%) deceased. Median age at diagnosis was 61 years (IQR 16), and median follow-up was 628 days (IQR 789.5).

**Table 1:** Patient and tumour characteristics

Variable	Category / Summary	Value
Sex	Male	385 (73.1%)
	Female	142 (26.9%)
Race	White	451 (85.6%)
	Black or African American	48 (9.1%)
	Asian	11 (2.1%)
	American Indian or Alaska Native	2 (0.4%)
	Missing	15 (2.8%)
Clinical stage	Stage I	19 (3.6%)
	Stage II	83 (15.7%)
	Stage III	96 (18.2%)
	Stage IVA	204 (38.7%)
	Stage IVB	4 (0.8%)
	Stage IVC	6 (1.1%)
	Missing	115 (21.8%)
Pathologic stage	Stage I	24 (4.6%)
	Stage II	69 (13.1%)
	Stage III	68 (12.9%)
	Stage IVA	199 (37.8%)
	Stage IVB	10 (1.9%)
	Stage IVC	1 (0.2%)
	Missing	155 (29.4%)
Tumour grade	G1	51 (9.7%)
	G2	247 (46.9%)
	G3	99 (18.8%)
	G4	5 (0.9%)
	GX	17 (3.2%)
	Missing	108 (20.5%)
HPV status	HPV+	72 (13.7%)
	HPV−	448 (85.0%)
	Missing	7 (1.3%)
Vital status	Alive	303 (57.5%)
	Dead	224 (42.5%)
	Missing	0 (0.0%)
Continuous variables (median [IQR])	Age at diagnosis (years)	61.00 [16.00]
	Follow-up (days)	628.00 [789.50]
	TMB (mut/Mb)	2.75 [2.38]
	Neo-epitopes (neo_n_500)	1242.00 [1183.00]
	CYT score	6.38 [11.21]
	PD-L1 (CD274_expr)	77.74 [134.25]
	TIDE dysfunction	0.02 [1.01]
	TIDE exclusion	0.08 [1.55]
	CD8 fraction	0.10 [0.11]
	Macrophages M1 fraction	0.07 [0.10]
	Macrophages M2 fraction	0.19 [0.12]
	Treg fraction	0.02 [0.04]

Counts are n (%) for categorical variables and medians with interquartile ranges (IQR) for continuous variables. “Missing” denotes unavailable clinical annotations. TMB is reported in mutations per megabase. neo_n_500 denotes the predicted number of MHC-I binding neoepitopes per tumour. CYT, PD-L1 (CD274_expr), TIDE dysfunction/exclusion and CIBERSORT cell fractions (CD8, M1, M2, Tregs) are shown as cohort medians (IQR). Missing entries: TMB (*n* = 20), neo_n_500 (*n* = 22)

Across the cohort, the median tumour mutational burden (TMB) was 2.75 mut/Mb (IQR 2.38), and the median predicted MHC-I neoepitope count (neo_n_500) was 1,242 peptides (IQR 1,183). CYT (cytolytic activity) had a median of 6.38 (IQR 11.21), PD-L1 (CD274) expression a median of 77.74 (IQR 134.25), TIDE dysfunction a median of 0.02 (IQR 1.01) and TIDE exclusion a median of 0.08 (IQR 1.55). Deconvolution of the tumour microenvironment (CIBERSORT) yielded median fractions of CD8⁺ T cells 0.10 (IQR 0.11), macrophages M1 0.07 (IQR 0.10), macrophages M2 0.19 (IQR 0.12) and regulatory T cells (Tregs) 0.02 (IQR 0.04).

Notably, TIDE dysfunction scores showed a wide range (median 0.02, IQR 1.01), reflecting substantial heterogeneity in T-cell infiltration across the cohort. As detailed below, we found that this heterogeneity is strongly associated with neoantigen clonality, with high-clonality tumours exhibiting paradoxically reduced immune infiltration.

**Fig. 1:**
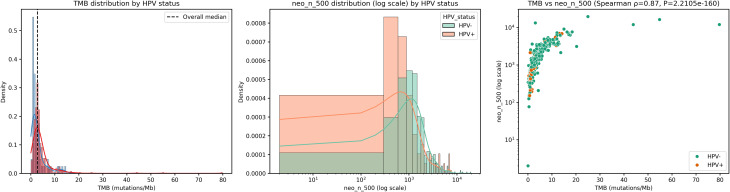
Distributions of mutational and neoepitope load and their association. **a**, Kernel density and histogram of tumour mutational burden (TMB, mutations per megabase) coloured by HPV status; dashed line indicates overall median. **b**, Kernel density and histogram of predicted MHC-I neoepitope count (neo_n_500) on a logarithmic x-axis, coloured by HPV status. **c**, Scatter plot of TMB versus neo_n_500 (log-scaled y-axis) with HPV status indicated and Spearman correlation reported (ρ = 0.87; *P* = 2.21 × 10⁻¹⁶⁰). Group comparisons between HPV-positive and HPV-negative tumours used Shapiro–Wilk normality tests followed by two-sided Mann–Whitney U tests due to non-normal distributions

Distributions of TMB and neoepitope burden were right-skewed (Fig. 1a, b), motivating non-parametric comparisons between HPV strata. Shapiro–Wilk tests confirmed non-normality in both HPV-positive and HPV-negative groups (*P* < 0.0001 for each). HPV-positive tumours had significantly lower TMB than HPV-negative tumours (median 1.60 vs. 2.93 mut/Mb; two-sided Mann–Whitney U = 9,244.5; *P* = 3.44 × 10⁻⁶). Neo-epitope counts were also significantly lower in HPV-positive tumours (median 738 vs. 1,301 peptides; U = 9,287.0; *P* = 9.73 × 10⁻⁶). As expected, TMB and neoepitope load were strongly correlated (Spearman ρ = 0.87; *P* = 2.21 × 10⁻¹⁶⁰; Fig. 1c).

### Neo-antigen quantity versus quality

**Fig. 2:**
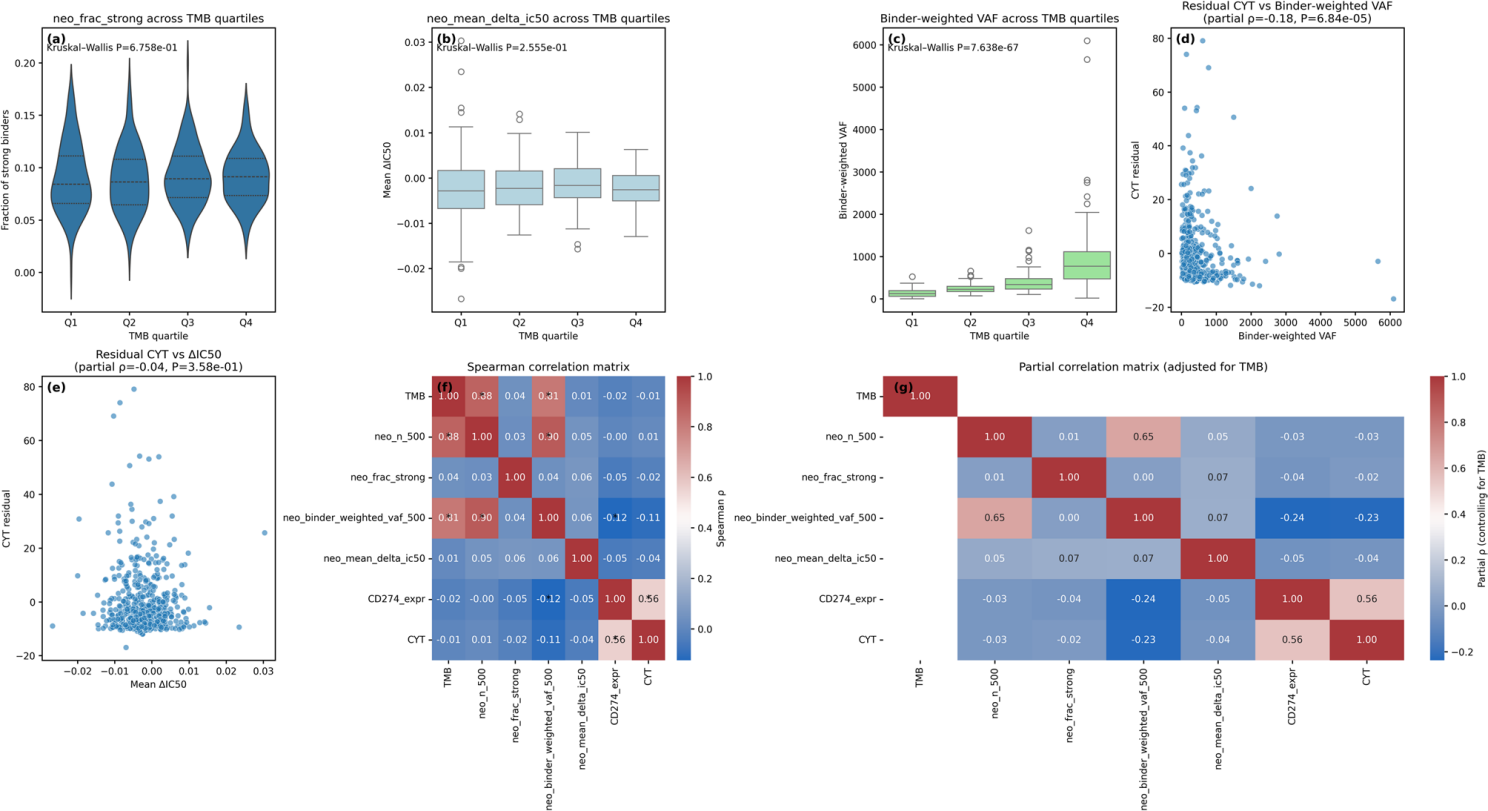
Neo-antigen quantity and quality in HNSCC, and their relationships with the immune microenvironment. **a**, Fraction of strong binders (neo_frac_strong) across TMB quartiles (Q1–Q4). No overall difference (Kruskal–Wallis *P* = 0.676). **b**, Mean ΔIC50 (mutant–wild-type) across TMB quartiles (*P* = 0.256). **c**, Binder-weighted VAF across TMB quartiles (*P* = 7.64 × 10⁻⁶⁷); medians rise from 126 to 773 across Q1–Q4. **d**, Partial-residual plot: CYT residual (after regressing on TMB) vs. binder-weighted VAF (partial Spearman ρ = −0.18, *P* = 6.84 × 10⁻⁵; *n* = 495). **e**, CYT residual vs. ΔIC50 (ρ = −0.04, *P* = 0.358; *n* = 495). **f**, Spearman correlation heatmap among TMB, neo_n_500, neo_frac_strong, binder-weighted VAF, ΔIC50, PD-L1 (CD274_expr) and CYT. Asterisks denote FDR-significant pairs (FDR < 0.05). **g**, Partial correlation heatmap for the same variables after controlling for TMB. Plots show medians with interquartile ranges where appropriate; P values from Kruskal–Wallis or Spearman tests as indicated

We first examined how qualitative properties of predicted MHC-I neoepitopes relate to mutation burden and the tumour immune state (Fig. 2). Across TMB quartiles (cut-points: Q1 0.03–1.80, Q2 1.80–2.75, Q3 2.75–4.18, Q4 4.18–79.76 mut/Mb), the fraction of strong binders (neo_frac_strong) was broadly stable (medians 0.084–0.091) with no evidence for a monotonic trend (Kruskal–Wallis H = 1.53, df = 3, *P* = 0.676; Spearman ρ = 0.040, *P* = 0.365; *n* = 505). Similarly, the average change in predicted binding affinity relative to the corresponding wild-type peptide (neo_mean_delta_ic50) did not differ across TMB strata (medians − 2.86 × 10⁻³ to − 1.65 × 10⁻³; H = 4.06, *P* = 0.256; Spearman ρ = 0.014, *P* = 0.759; *n* = 505). By contrast, binder-weighted clonal burden (binder-weighted VAF) increased sharply with TMB (medians Q1–Q4: 126, 228, 337, 773; H = 309.77, *P* = 7.64 × 10⁻⁶⁷; Spearman ρ = 0.810, *P* = 8.61 × 10⁻¹¹⁹; *n* = 505), reflecting that quantity scales with mutation load even when binder quality metrics remain relatively constant.

To test whether neoantigen quality carries information beyond TMB, we residualised CYT on TMB and correlated the residuals with neo-metrics. Higher binder-weighted VAF was associated with lower CYT after controlling for TMB (partial Spearman ρ = −0.178, *P* = 6.84 × 10⁻⁵; *n* = 495), whereas ΔIC50 showed no association (ρ = −0.041, *P* = 0.358; *n* = 495) (Fig. 2d–e; Supplementary Table S1). These findings indicate that clonal neoantigen load, when emphasised by predicted binding, is linked to T-cell suppression even at fixed TMB, whereas average affinity shift alone is not. “Clonal” here refers to the VAF weighting of binder counts, which enriches for variants present in a larger fraction of tumour cells.

Global associations among neoantigen metrics and immune features are summarised in correlation heatmaps (Fig. 2f–g). Spearman correlations identified ten FDR-significant pairs (FDR < 0.05), dominated by the expected positive co-variation of TMB with total neoantigens (ρ = 0.876; FDR = 5.66 × 10⁻¹⁵⁸) and of neo_n_500 with binder-weighted VAF (ρ = 0.896; FDR = 1.34 × 10⁻¹⁷⁵). Notably, binder-weighted VAF correlated inversely with PD-L1 expression (ρ = −0.125; FDR = 0.043), and strongly with TIDE dysfunction (ρ = −0.304; FDR = 1.01 × 10⁻¹⁰), suggesting that tumours with heavier clonal binder loads exhibit signatures of T-cell dysfunction. Partial correlations controlling for TMB (Fig. 2g) preserved the negative relationships between binder-weighted VAF and CYT/PD-L1, underscoring that these associations are not explained by mutation burden alone.

### Immune microenvironment and heterogeneity

We next explored how neoantigen features relate to T cell dysfunction/exclusion, PD-L1 expression and the broader immune contexture.

#### Clonal neoantigen burden predicts T-cell dysfunction

**Fig. 3:**
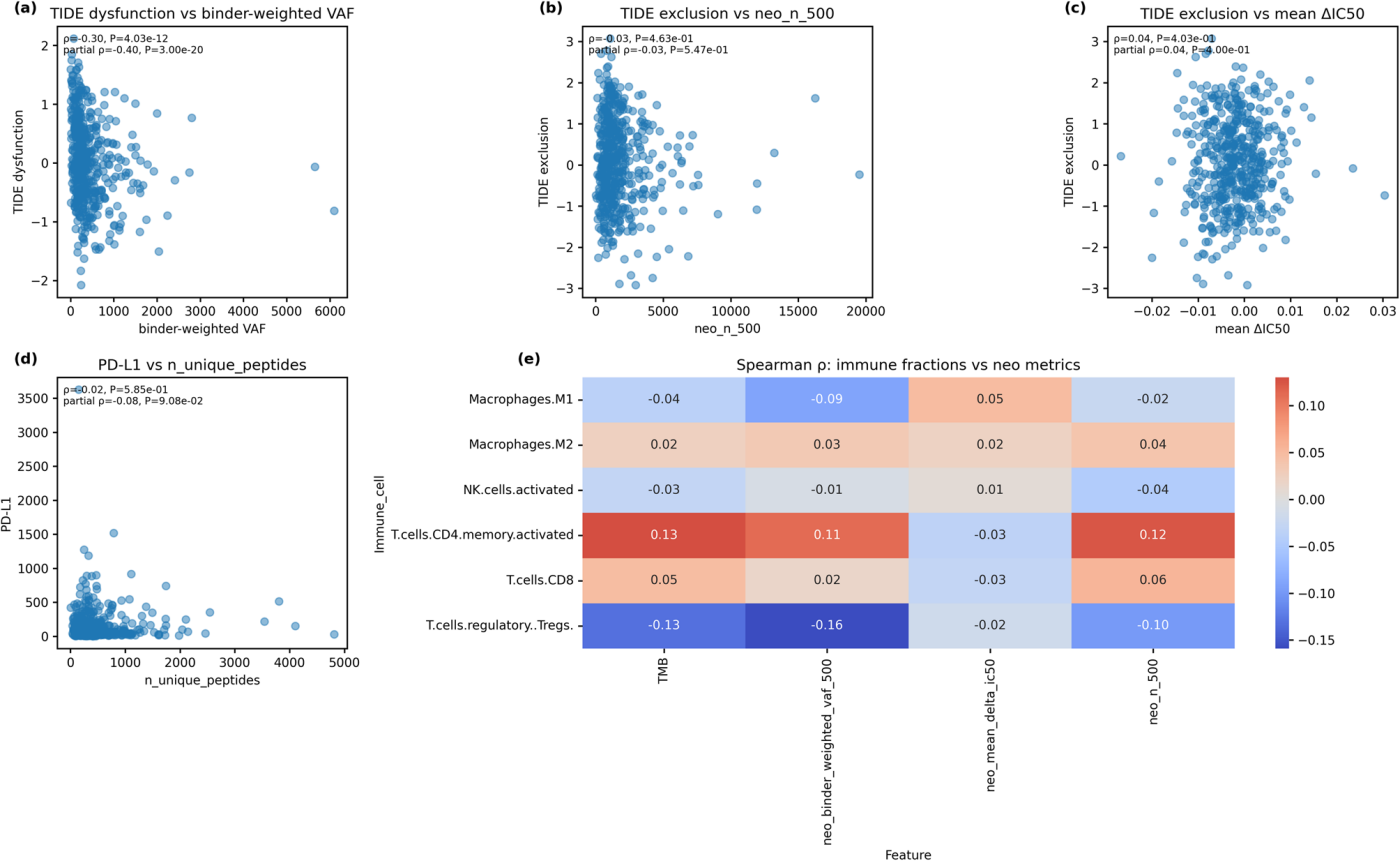
Immune contexture vs. neoantigen metrics. **a**, Scatter plot showing a strong inverse relationship between TIDE dysfunction and binder-weighted clonal neoantigen burden (Spearman ρ = −0.304, *P* = 4.03 × 10⁻¹²; partial ρ = −0.397, *P* = 3.00 × 10⁻²⁰). **b**, TIDE exclusion vs. total predicted neoantigen count (neo_n_500); no significant correlation observed (ρ = −0.033, *P* = 0.463). **c**, TIDE exclusion vs. mean affinity change (ΔIC50); no significant correlation (ρ = 0.038, *P* = 0.403). **d**, PD-L1 expression vs. number of unique predicted neoepitopes; weak non-significant correlation. **e**, Heatmap summarising Spearman correlations (ρ) between immune-cell fractions (rows) and neoantigen or mutation features (columns). Asterisks in the matrix denote FDR-significant correlations (*P* < 0.05 after Benjamini–Hochberg correction). Activated memory CD4 T cells correlate positively with TMB and neo_n_500, whereas Tregs correlate inversely with TMB and binder-weighted VAF

The strongest association involved TIDE dysfunction and binder-weighted clonal burden. Tumours with a higher clonal neoantigen load showed markedly lower predicted T-cell dysfunction (Spearman ρ = −0.304, *P* = 4.03 × 10⁻¹²), indicating reduced T‑cell infiltration. This relationship became even stronger after adjusting for TMB (partial ρ = −0.397, *P* = 3.00 × 10⁻²⁰), indicating that the link is not merely due to mutation burden (Fig. 3a). By contrast, neither the total predicted neoantigen count nor the average binding affinity change (ΔIC50) correlated with TIDE exclusion (Spearman ρ = −0.033 and 0.038; both *P* > 0.40; Fig. 3b–c). These initial findings suggested that clonal neoantigen burden associates with reduced TIDE dysfunction scores. However, as we demonstrate below, this relationship is more nuanced: when clonality is normalized for neoantigen count, high clonality paradoxically identifies immunologically “cold” tumours with reduced T-cell infiltration rather than driving exhaustion in infiltrated T cells. PD-L1 expression was also weakly related to neoantigen diversity: the number of unique predicted peptides per tumour did not correlate significantly with PD-L1 either before or after TMB adjustment (ρ = −0.025, *P* = 0.586; partial ρ = −0.076, *P* = 0.091; Fig. 3d).

#### Immune-cell fractions reflect mutation burden and clonal antigen load

We next correlated CIBERSORT-estimated immune-cell fractions with TMB and key neoantigen metrics (Fig. 3e; Supplementary Table S2). After FDR correction, four associations remained significant (FDR < 0.05). Memory-activated CD4 T cells correlated positively with both TMB (ρ = 0.130, FDR = 2.88 × 10⁻²) and total neoantigen count (ρ = 0.124, FDR = 3.29 × 10⁻²), whereas regulatory T cells (Tregs) correlated inversely with TMB (ρ = −0.131, FDR = 2.88 × 10⁻²) and with binder-weighted clonal burden (ρ = −0.159, FDR = 8.92 × 10⁻³). No significant associations were observed for CD8 T cells, NK cells, or macrophage subsets. These results suggest that tumours with higher mutation load and neoantigen abundance recruit more activated CD4 T cells but fewer Tregs, whereas the quality of clonal neoantigens (binder-weighted VAF) is specifically linked to T-cell dysfunction rather than broad immune infiltration.

### Tumour heterogeneity, HPV status and HLA loss of heterozygosity

**Fig. 4:**
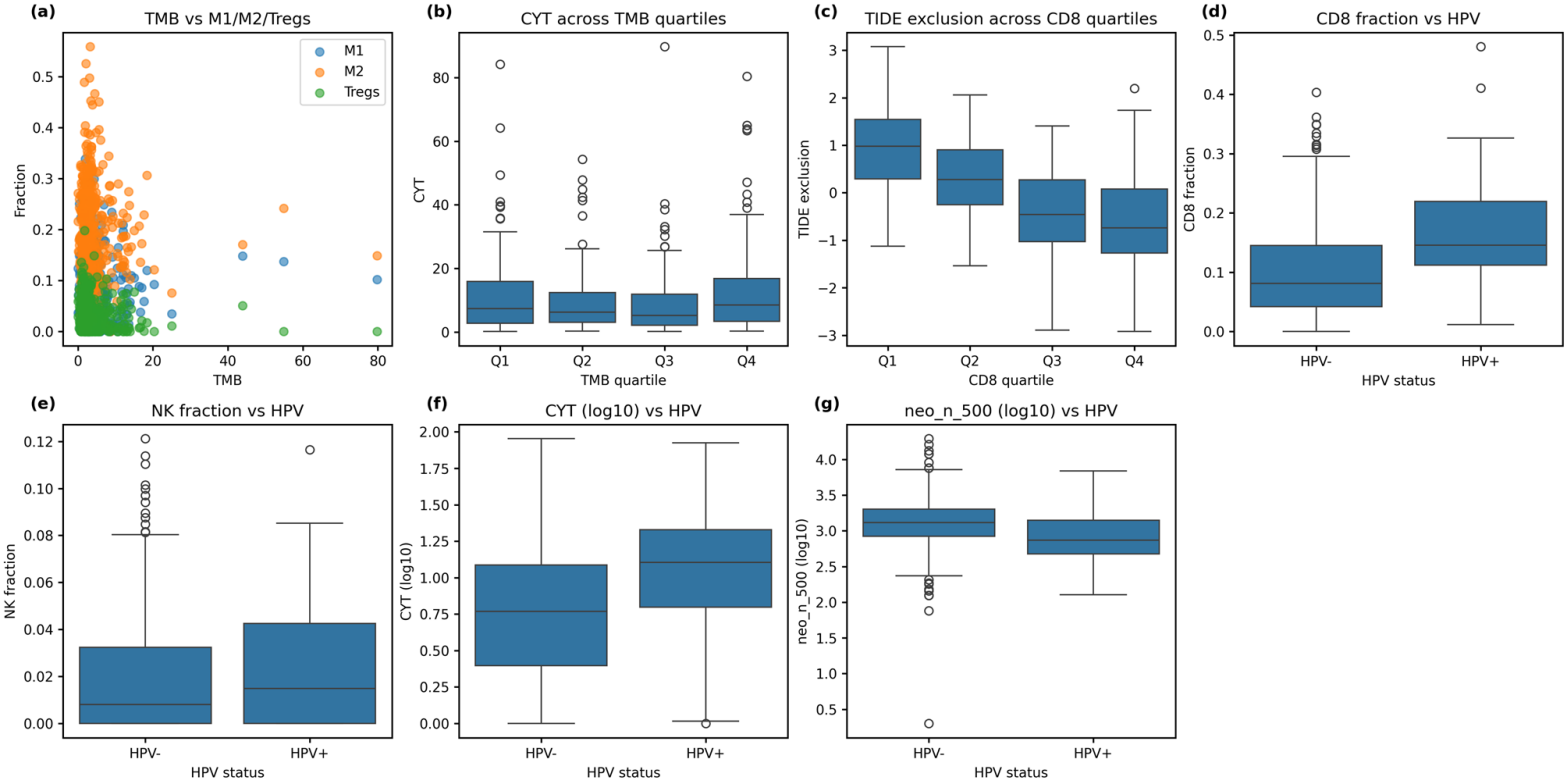
Tumour heterogeneity and HPV stratification. **a**, Scatter plots of fractions of M1 and M2 macrophages and regulatory T cells versus TMB. **b**, Boxplots of CYT scores across TMB quartiles (Q1–Q4). **c**, Boxplots of TIDE exclusion scores across CD8 quartiles. **d**, Boxplots comparing CD8 fractions between HPV-positive and HPV-negative tumours. **e**, Boxplots comparing NK fractions between HPV groups. **f**, Boxplots comparing log₁₀ CYT scores by HPV status. **g**, Boxplots comparing log₁₀ neoepitope counts (neo_n_500) by HPV status. Panels are labelled (**a**–**g**)

We assessed how the immune microenvironment varies with mutation load, HPV status and HLA LOH in the HNSCC cohort. TMB showed little association with macrophage polarization: M1 and M2 fractions were uncorrelated with mutation load (ρ = − 0.037 and 0.024; *P* > 0.40), whereas a modest negative correlation was observed for regulatory T cells (Tregs; ρ = − 0.131; *P* = 3.5 × 10⁻³). These relationships are visualised in Fig. 4a, where M1, M2 and Treg fractions are plotted against TMB. Cytolytic activity (CYT) varied across TMB quartiles (median 7.42 in Q1, 6.24 in Q2, 5.17 in Q3 and 8.49 in Q4), but the trend was not significant (Kruskal–Wallis H = 6.14, *P* = 0.10; Fig. 4b). Immune exclusion decreased sharply across CD8 T-cell quartiles (median 0.98 in Q1 vs. − 0.74 in Q4, H = 172.75, *P* = 3.24 × 10⁻³⁷; Fig. 4c), whereas NK quartiles showed no significant trend (H = 6.19, *P* = 0.10). HPV-positive tumours exhibited significantly higher CD8 T-cell infiltration (median 0.146 vs. 0.081; *P* = 1.67 × 10⁻¹⁰; Fig. 4d) and cytolytic activity (median 12.71 vs. 5.86; *P* = 4.24 × 10⁻⁷; Fig. 4f) compared to HPV-negative tumours, consistent with the known immunogenicity of HPV-driven HNSCC. NK cell fractions did not differ significantly between groups (median 0.015 vs. 0.008; *P* = 0.16; Fig. 4e). Notably, HPV-positive tumours harboured significantly fewer predicted neoepitopes (median 738 vs. 1,301; *P* = 9.73 × 10⁻⁶; Fig. 4g), reflecting their lower mutational burden. These results indicate that while mutation load influences Treg infiltration and immune exclusion, HPV status is a strong determinant of T-cell infiltration and cytolytic activity independent of neoantigen burden.

**Fig. 5:**
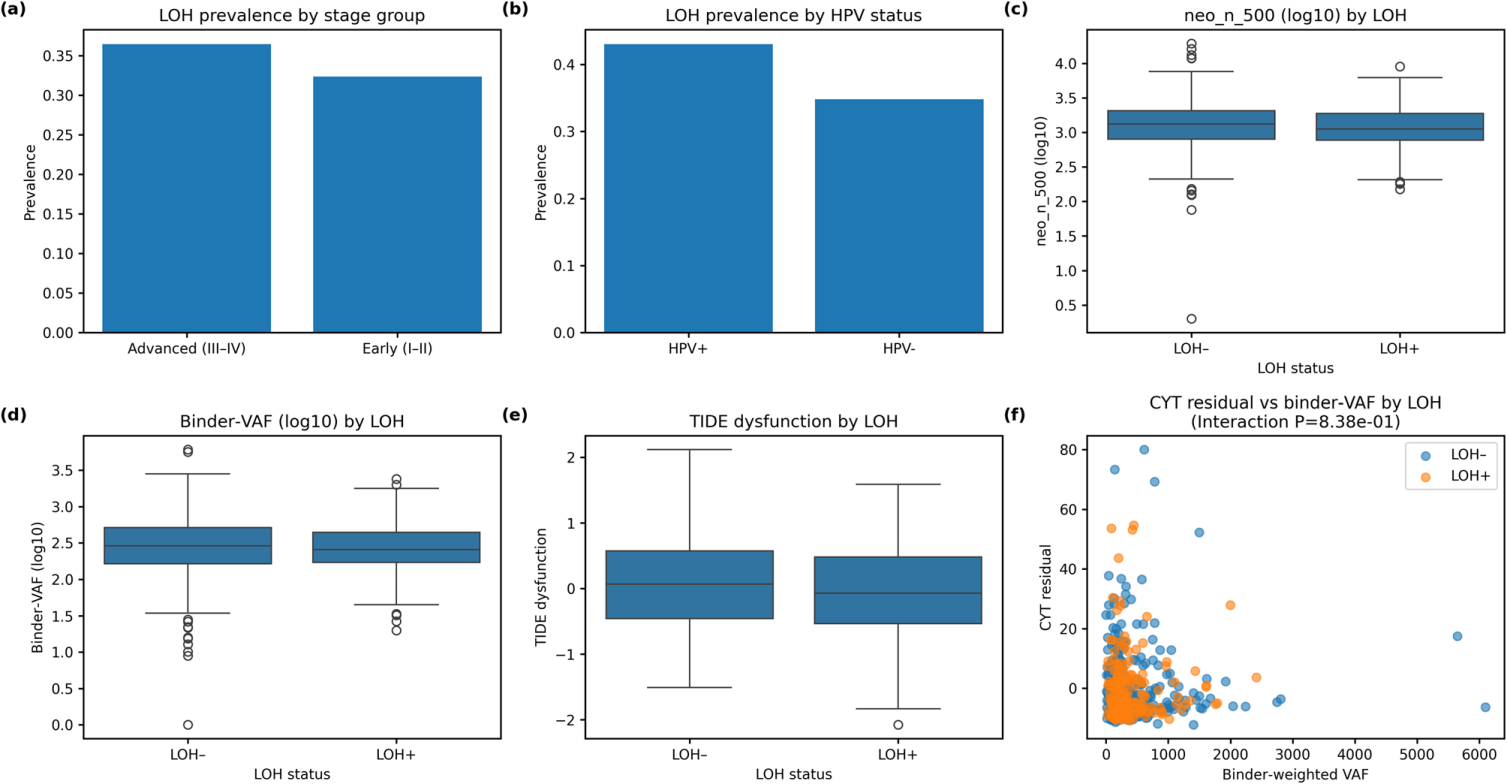
HLA loss of heterozygosity (LOH) and antigen-presentation defects. **a**, Bar plot showing the prevalence of LOH stratified by stage group. **b**, Bar plot showing LOH prevalence stratified by HPV status. **c**, Boxplots of log₁₀ neoepitope counts (neo_n_500) in LOH-positive and LOH-negative tumours. **d**, Boxplots of log₁₀ binder-weighted clonal neoantigen burden (binder-VAF) by LOH status. **e**, Boxplots of TIDE dysfunction scores by LOH status. **f**, Partial residual plot of CYT (adjusted for TMB) versus binder-weighted VAF, stratified by LOH status; the inverse relationship between binder-weighted VAF and CYT is similar in LOH-positive and LOH-negative tumours, and the interaction is not significant. Panels are labelled (**a**–**f**)

LOH occurred in 36.5% of advanced-stage tumours and 32.4% of early-stage tumours, and was more common in HPV-positive than HPV-negative cases (43.1% vs. 34.8%; Fig. 5a–b). LOH-positive tumours did not show significantly different median neoepitope counts (1,124 vs. 1,329; *P* = 0.145), binder-weighted VAF (256 vs. 287; *P* = 0.599), TMB (2.66 vs. 2.84; *P* = 0.464) or CYT (6.31 vs. 6.85; *P* = 0.442) (Supplementary Table S3). The only significant difference was a lower TIDE dysfunction score in LOH-positive tumours (median − 0.071 vs. 0.067; *P* = 0.048), suggesting reduced T-cell infiltration despite similar neoepitope loads. These comparisons are shown in Fig. 5c–e, where neo_n_500 and binder-weighted VAF are log₁₀-transformed for clarity. A linear model of CYT (dependent variable) including binder-weighted VAF, LOH status and their interaction plus TMB confirmed that binder-weighted VAF was inversely associated with CYT (β = − 0.0056; *P* = 0.002), TMB was positively associated (β = 0.603; *P* < 0.001) and the VAF × LOH interaction was not significant (*P* = 0.838); the partial-residual plot in Fig. 5f illustrates that the inverse relationship between binder-weighted VAF and CYT holds irrespective of LOH status. These findings suggest that clonal neoantigen burden suppresses cytolytic activity independent of HLA LOH, and that LOH itself exerts only a modest effect on T-cell dysfunction without materially altering neoantigen load or binding strength.

### Gene-centric neoepitope landscape

**Fig. 6:**
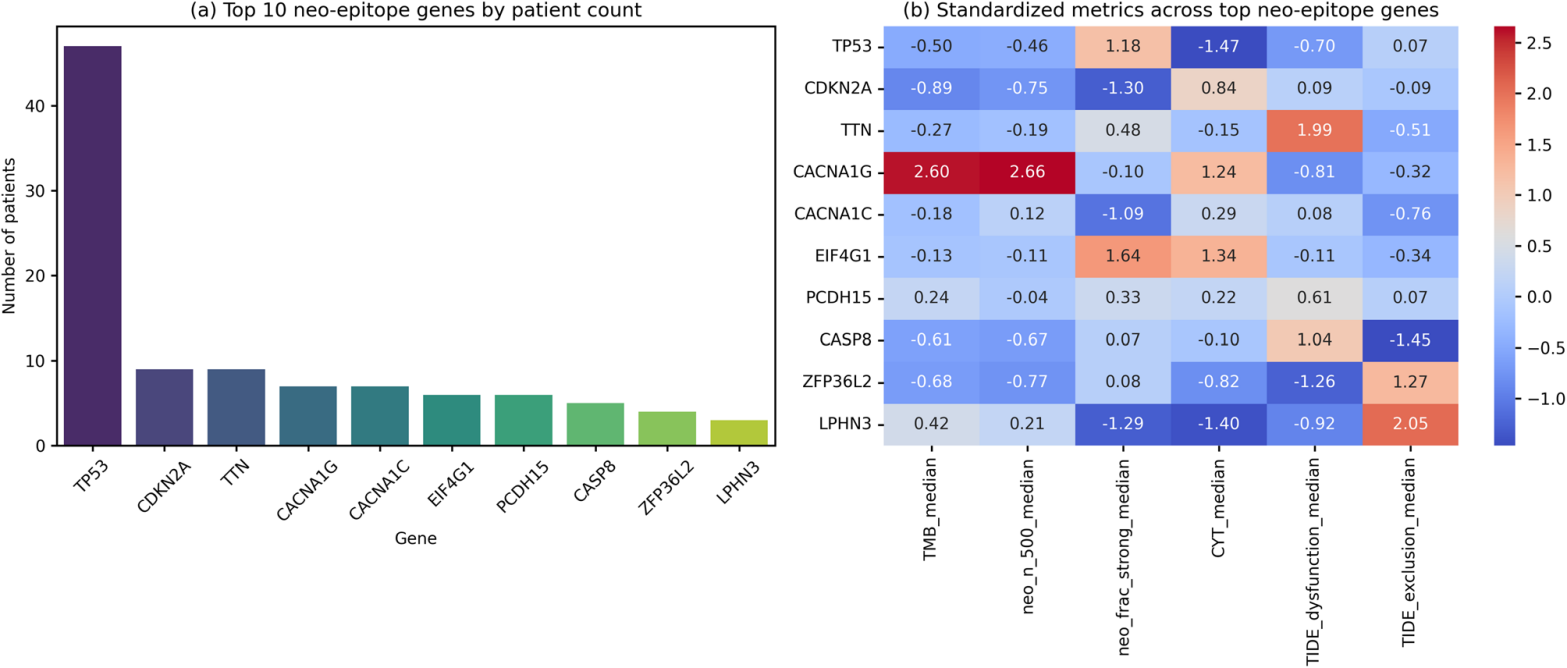
Gene-centric neoepitope landscape — summary panels. **a**, Bar chart showing the number of patients for whom each gene is the dominant neoantigen source (producing the highest binder count) in the cohort; *TP53* dominates with 47 cases, while *CDKN2A*, *TTN*, *CACNA1G*, *CACNA1C*, *EIF4G1*, *PCDH15*, *CASP8*, *ZFP36L2* and *LPHN3* occur in far fewer tumours. **b**, Heatmap displaying z-scored values of tumour mutational burden (TMB), predicted neoepitope count (neo_n_500), fraction of strong binders, cytolytic activity (CYT), TIDE dysfunction and TIDE exclusion for the same genes; the heatmap highlights gene-specific patterns, such as *CACNA1G* showing very high mutation and neoepitope burdens but relatively low TIDE dysfunction, whereas *ZFP36L2* exhibits high TIDE exclusion despite moderate neoantigen metrics

We analysed the genes generating the largest numbers of predicted neoepitope binders in HNSCC and summarised their immunological context (Fig. 6; Table 2). The top ten genes were *TP53*, *CDKN2A*, *TTN*, *CACNA1G*, *CACNA1C*, *EIF4G1*, *PCDH15*, *CASP8*, *ZFP36L2* and *LPHN3* (*n* = 47, 9, 9, 7, 7, 6, 6, 5, 4 and 3 patients, respectively).

- *TP53*, the most frequently mutated gene, showed moderate TMB and neoepitope burden but relatively low CYT and CD8 infiltration, consistent with its role as a tumour-suppressor whose mutations promote immune evasion.
- *CACNA1G* (calcium channel) mutations were associated with extremely high TMB and neoepitope counts, alongside high CYT, suggesting that this gene’s neoepitopes may be highly immunogenic.
- *CASP8* (caspase-8) mutations tended to occur in tumours with moderate TMB and binder-weighted VAF but modest immune infiltration; caspase-8 loss promotes resistance to apoptosis and may enable immune escape.
- *ZFP36L2* (RNA-binding protein) mutations were rare but linked to the highest median TIDE exclusion scores and low TIDE dysfunction, suggesting an immunosuppressive microenvironment.

**Table 2:** Summary of top neoepitope genes and immunological context

Gene	*n*	TMB (median)	TIDE dysfunction (median)	TIDE exclusion (median)	PDL1 (median)	neo_n_500 (median)	neo_frac_strong (median)	neo_best_ic50 (median)	neo_npi (median)	binderVAF (median)	Unique peptides (median)	Public peptides (median)	CYT (median)	CD8 fraction (median)	Treg fraction (median)
TP53	47	2.93	–0.26	0.49	50.64	1 347	0.093	22.90	0	258.31	359	11	3.91	0.081	0.0126
CDKN2A	9	2.31	–0.00	0.38	74.25	1 153	0.0768	24.19	0	201.62	297	15	8.74	0.114	0.0186
TTN	9	3.29	0.62	0.09	104.91	1 533	0.0887	21.49	0	327.23	364	7	6.67	0.115	0.0166
CACNA1G	7	7.90	–0.29	0.22	215.72	3 490	0.0848	19.38	0	914.04	791	5	9.58	0.149	0.00255
CACNA1C	7	3.44	–0.005	–0.09	72.63	1 746	0.0782	21.48	0	507.00	424	9	7.59	0.0863	0.00865
EIF4G1	6	3.52	–0.064	0.21	110.17	1 591	0.0965	14.99	0	396.07	394	4	9.79	0.0969	0.0189
PCDH15	6	4.12	0.17	0.49	68.87	1 634	0.0877	22.73	0	405.44	359	1.5	7.44	0.0688	0.0146
CASP8	5	2.75	0.31	–0.56	129.16	1 204	0.0860	14.79	0	254.97	269	6	6.76	0.0590	0.00950
ZFP36L2	4	2.65	–0.44	1.32	14.55	1 137	0.0861	22.39	0	334.93	390	94	5.27	0.0760	0.0204
LPHN3	3	4.40	–0.33	1.86	42.16	1 810	0.0768	24.25	0	356.95	441	3	4.04	0.0331	0.00004

This table lists the ten genes most frequently identified as the dominant neoantigen-producing gene per patient (i.e., generating the highest number of predicted MHC-I binding neoantigens within each tumour). For each gene, we report the number of patients (n), median tumour mutational burden (TMB), median TIDE dysfunction and TIDE exclusion scores, median PD-L1 expression (CD274), median predicted neoepitope count (neo_n_500), fraction of strong binders, best predicted binding affinity (neo_best_ic50), neo-presentation index (NPI; zero for all genes in this dataset), binder-weighted clonal neoantigen burden (binder-VAF), numbers of unique and public peptides, cytolytic activity (CYT), CD8 T-cell fraction and regulatory T-cell (Treg) fraction

**Fig. 7:**
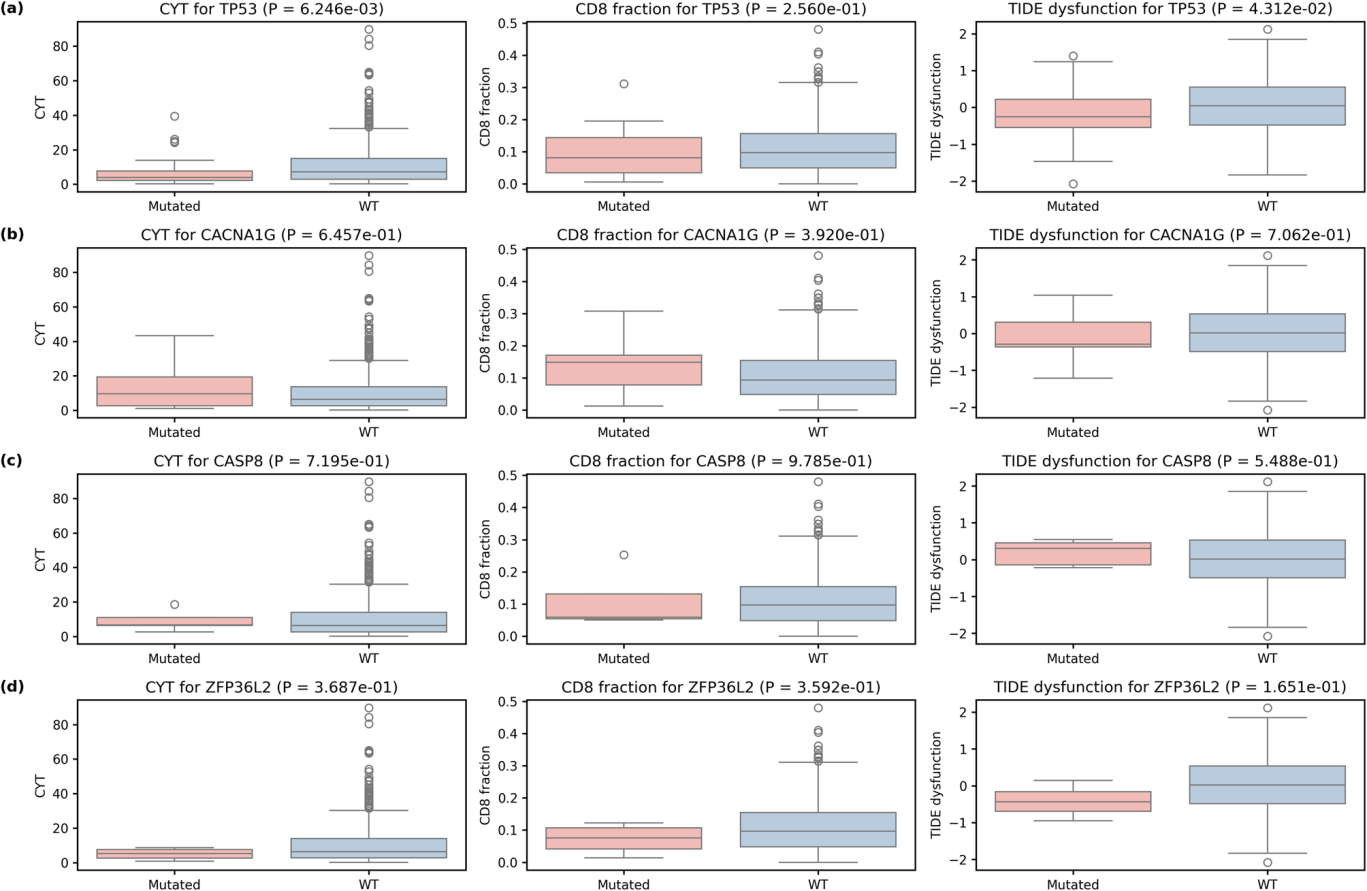
Immune context of selected neoepitope genes. Boxplots comparing tumours mutated in (**a**) TP53, (**b**) CACNA1G, (**c**) CASP8 and (**d**) ZFP36L2 with all other (wild-type) tumours for three metrics: cytolytic activity (CYT; left column), CD8 T-cell fraction (middle column) and TIDE dysfunction score (right column). Mutated’ indicates tumours where the gene is the dominant neoantigen producer; ‘WT’ indicates tumours where other genes dominate, regardless of mutation status. Each panel reports the two-sided Mann–Whitney *P* value. TP53 mutations are associated with significantly lower CYT (median 3.9 vs 7.1; P = 6.25 × 10⁻³) and lower TIDE dysfunction scores (median − 0.26 vs 0.04; P = 4.31 × 10⁻²) relative to wild-type tumours. Mutations in *CACNA1G*, *CASP8* and *ZFP36L2* do not significantly alter CYT, CD8 infiltration or TIDE dysfunction

Boxplots comparing mutated vs. wild-type tumours for selected genes (Fig. 7) revealed that TP53-mutant tumours had significantly lower CYT scores than wild-type tumours (median 3.9 vs. 7.1; Mann–Whitney *P* = 6.25 × 10⁻³) and displayed lower TIDE dysfunction scores (median − 0.26 vs. 0.04; *P* = 4.31 × 10⁻²). CASP8 and ZFP36L2 mutations did not show significant differences in CYT, CACNA1G, CD8 infiltration or TIDE dysfunction.

### Translational proof of concept

To explore the feasibility of off-the-shelf neoantigen vaccines, we assessed the distribution and population coverage of predicted public (shared) and private (patient-specific) neoepitope peptides. Across the 527 tumours, only 4 305 peptides (≈ 2%) were predicted to be public, whereas 228 618 peptides were private, underscoring the rarity of recurring neoepitopes. The average patient harboured just 8.52 public peptides (median 4; IQR = 8; maximum 155), indicating that most tumours contribute few shared epitopes to a common vaccine pool.

**Fig. 8:**
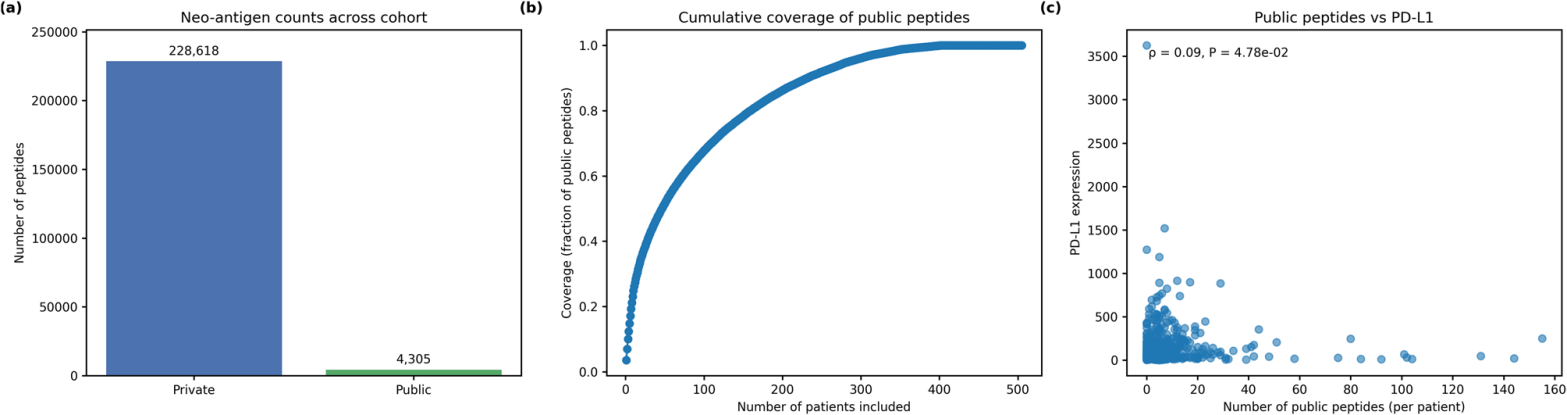
Population coverage of public neoepitopes. **a**, Bar chart comparing the total number of predicted private versus public neoepitope peptides across all tumours; 228 618 peptides are private and only 4 305 are public. Counts are displayed above each bar. **b**, Set-cover curve showing the cumulative fraction of all public peptides captured as patients are added in descending order of their public peptide counts. Coverage rises steeply: the first few dozen patients cover a large fraction of public peptides, but the curve plateaus as increasingly rare peptides are included. **c**, Scatter plot of the number of public peptides per patient versus PD-L1 expression, with the Spearman correlation coefficient (ρ = 0.09) and P value (*P* = 4.78 × 10⁻²) annotated; the weak positive correlation suggests that tumours with more public peptides tend to express slightly higher PD-L1

We ranked patients by their number of public peptides and constructed a “set cover” curve (Fig. 8b). Coverage increased rapidly: the top 50 patients accounted for about half of all predicted public peptides, while the top 200 patients covered nearly 90%. However, the curve plateaued thereafter, suggesting diminishing returns as additional patients contribute increasingly rare public peptides. These results imply that, although an off-the-shelf vaccine targeting a handful of public peptides could benefit a subset of patients, most tumours would still require personalised neoantigen approaches.

Finally, we examined whether the number of public peptides per patient correlated with immunological markers. Public peptide burden showed a weak positive correlation with PD-L1 expression (Spearman ρ = 0.089, *P* = 4.78 × 10⁻²) and no significant correlation with cytolytic activity (ρ = 0.061, *P* = 0.178). Thus, tumours rich in public neoepitopes are only slightly more likely to up-regulate PD-L1 and do not display heightened cytolytic activity, suggesting that public peptides alone are insufficient to drive robust anti-tumour immunity.

**Fig. 9:**
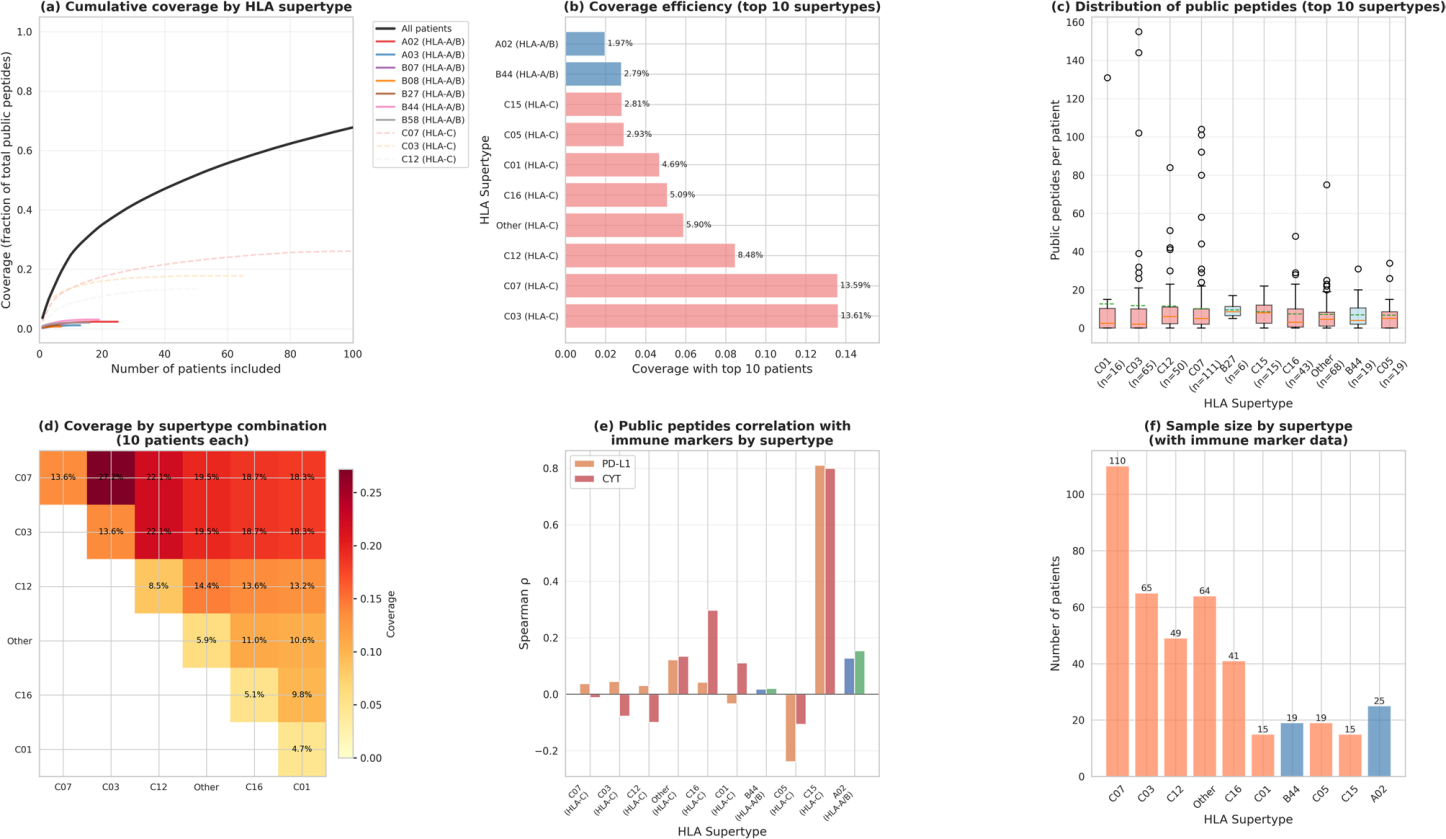
HLA supertype-stratified public peptide coverage and immune correlations. **a**, Cumulative coverage curves showing the fraction of total public peptides captured as patients are added, stratified by HLA supertype. HLA-A/B supertypes (solid coloured lines) and HLA-C supertypes (dashed lines) are shown separately; the black line denotes coverage across all patients regardless of supertype. HLA-C supertypes (C07, C03, C12) dominate the public peptide landscape. **b**, Coverage efficiency for the top 10 supertypes, measured as the fraction of total public peptides captured using the top 10 patients within each supertype. HLA-A/B supertypes (blue bars) show lower efficiency than HLA-C supertypes (red/coral bars). **c**, Boxplots showing the distribution of public peptides per patient for the top 10 supertypes, with sample sizes indicated below each box. **d**, Heatmap of population coverage achieved by combining multiple HLA supertypes (10 patients each), demonstrating synergistic coverage when supertypes are targeted together. **e**, Spearman correlation coefficients between public peptide counts and PD-L1 expression (orange) or cytolytic activity (CYT; red) for each supertype. C15 shows strong positive correlations with both markers. **f**, Bar chart showing the number

To further inform multi-epitope vaccine development, we stratified public peptides by HLA supertype and evaluated population coverage (Fig. 9; Supplementary Table S4). Among 505 patients with complete HLA typing, HLA-C alleles predominated as the top-ranked allele (382/505, 75.6%), followed by HLA-A (47/505, 9.3%) and HLA-B (76/505, 15.0%). We identified 18 distinct HLA supertypes, of which C07 (*n* = 111), C03 (*n* = 65), C12 (*n* = 50), C16 (*n* = 43) and A02 (*n* = 25) were most prevalent (Fig. 9f).

Cumulative coverage curves demonstrated that HLA-C supertypes dominated the public peptide landscape (Fig. 9a). When patients were ranked by public peptide count within each supertype, C07 achieved 13.6% coverage of the total public peptide repertoire using only the top 10 patients, followed by C03 (13.6%) and C12 (8.5%). By contrast, HLA-A/B supertypes—which are typically prioritised for vaccine design due to higher cell-surface expression and better-characterised peptide binding—showed lower coverage efficiency: B44 achieved 2.8% coverage, A02 1.97% and B58 1.95% with the top 10 patients each (Fig. 9b). The distribution of public peptides per patient varied substantially across supertypes, with C03 showing the highest mean burden per patient (11.8 peptides) and A02 the lowest among well-represented groups (4.0 peptides; Fig. 9c).

Multi-supertype combination strategies substantially improved population coverage (Fig. 9d). Targeting the top five supertypes by total peptide count (C07, C03, C12, Other, C16) with 20 patients per supertype achieved 59.3% coverage of public peptides, compared to only 8.0% when restricted to the top four HLA-A/B supertypes (B44, A02, B58, B27). Notably, the top three HLA-C supertypes alone (C07, C03, C12) with 20 patients each captured 44.3% of public peptides, suggesting that HLA-C–restricted peptides may be underexplored targets for shared neoantigen vaccines.

We examined whether public peptide counts correlated with immune markers across supertypes (Fig. 9e; Supplementary Table S4). Among HLA-C supertypes, C15 showed a strong positive correlation between public peptide burden and both PD-L1 expression (Spearman ρ = 0.81, *P* = 2.5 × 10⁻⁴) and cytolytic activity (ρ = 0.80, *P* = 3.5 × 10⁻⁴), suggesting that patients with C15-restricted public peptides may exhibit enhanced immunogenicity. Other supertypes showed weak or non-significant correlations (|ρ| < 0.30), indicating that the relationship between public peptide burden and immune activation is HLA-context dependent. Among HLA-A/B supertypes, none achieved statistical significance for PD-L1 or CYT correlations, although sample sizes were limited (*n* = 6–25 per supertype).

These analyses suggest that off-the-shelf vaccine strategies targeting HLA-C supertypes, particularly C07 and C03, could provide broad population coverage, while HLA-A/B–restricted peptides remain valuable for patients with appropriate alleles (Table 3). A multi-supertype approach combining both HLA classes may optimise the balance between population reach and immunological potency.

**Table 3:** Population coverage by supertype selection strategy

Strategy	Supertypes	Pts/Supertype	Total Pts	Coverage
Top 2 HLA-A/B	B44, A02	10	20	4.8%
Top 3 HLA-A/B	B44, A02, B58	10	30	6.7%
Top 4 HLA-A/B	B44, A02, B58, B27	10	36	8.0%
Top 3 (any HLA)	C07, C03, C12	10	30	35.7%
Top 5 (any HLA)	C07, C03, C12, Other, C16	10	50	46.7%
Top 5 (any HLA)	C07, C03, C12, Other, C16	20	100	59.3%
Common HLA-A/B	A02, A03, B07, B08	10	34	4.6%

This table compares different supertype selection strategies for multi-epitope vaccine design. For each strategy, we report the supertypes included, the number of patients per supertype, total patients pooled, and the resulting population coverage (fraction of total public peptides). Targeting HLA-C supertypes maximises coverage, whereas restricting to HLA-A/B supertypes—though immunologically preferred—limits population reach

### Neoantigen clonality inversely correlates with immune infiltration and T-cell-associated transcriptional signatures

The analyses above demonstrated that binder-weighted clonal burden (an absolute measure that scales with TMB) associates with reduced TIDE dysfunction scores. However, binder-weighted VAF conflates two properties: clonality (average VAF per neoantigen) and burden (total neoantigen count). To isolate the effect of clonality independent of burden, we computed a Clonality Score normalized for neoantigen count (see Methods). We then integrated RNA-sequencing-derived T-cell exhaustion signatures to determine whether clonality per se—rather than absolute burden—predicts immune activity. Surprisingly, this analysis revealed a paradoxical inverse relationship.

#### Neoantigen clonality shows paradoxical inverse correlation with T-cell exhaustion

**Fig. 10:**
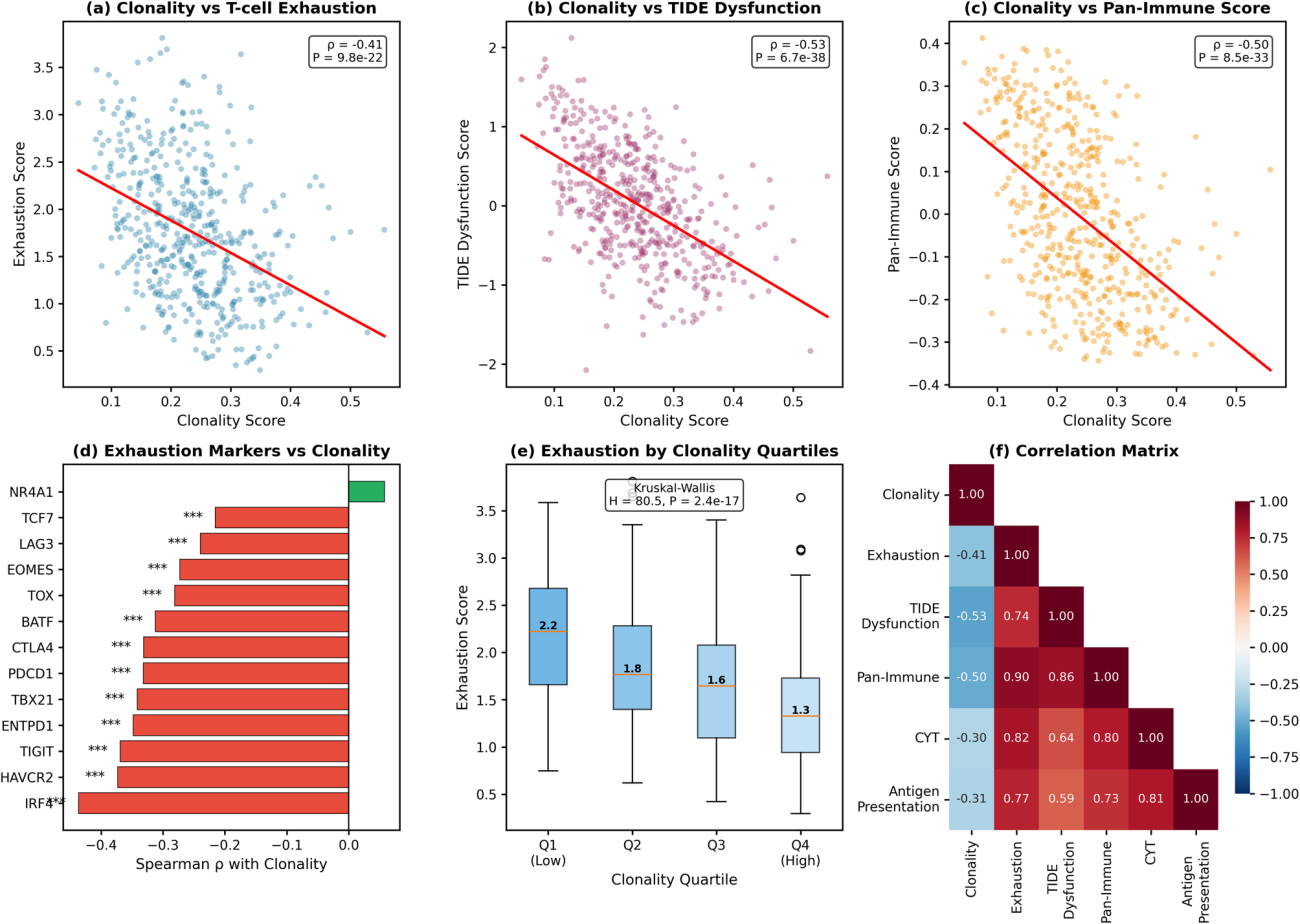
Neoantigen clonality inversely correlates with exhaustion-associated transcriptional signature and immune activity. **a**, Scatter plot demonstrating the negative correlation between clonality score and composite T-cell exhaustion score (ρ = −0.41, *P* = 9.8 × 10^− 22^). **b**, Clonality versus TIDE dysfunction score (ρ = −0.53, *P* = 6.7 × 10^− 38^). **c**, Clonality versus Pan-Immune Score from Budhwani et al. (ρ = −0.50, *P* = 8.5 × 10^− 33^). **d**, Horizontal bar chart showing Spearman correlations between individual exhaustion markers and clonality score. All markers except NR4A1 show significant negative correlations (***P < 0.001). **e**, Box plots showing exhaustion score distribution across clonality quartiles. Median values decrease progressively from Q1 (2.2) to Q4 (1.3); Kruskal-Wallis H = 80.5, *P* = 2.4 × 10^− 17^. **f**, Spearman correlation matrix showing relationships between clonality, exhaustion, TIDE dysfunction, Pan-Immune Score, cytolytic activity (CYT), and antigen presentation score. Red indicates positive correlations; blue indicates negative correlations

Contrary to the hypothesis that clonal neoantigens would drive T-cell exhaustion through persistent antigen exposure, we observed a robust inverse correlation between neoantigen clonality and T-cell-associated transcriptional signatures (Table 4). Because these metrics are derived from bulk RNA-seq, they reflect a combination of immune cell abundance and functional state; accordingly, reduced signatures in high-clonality tumours may primarily indicate diminished T-cell infiltration rather than altered exhaustion states per se. The clonality score demonstrated a strong negative correlation with our composite exhaustion signature (ρ = −0.412, *P* = 9.76 × 10^− 22^, *n* = 497; Fig. 10a). This inverse relationship was even more pronounced with TIDE dysfunction scores (ρ = −0.533, *P* = 6.71 × 10^− 38^; Fig. 10b) and the Pan-Immune Score from Budhwani et al. (ρ = −0.500, *P* = 8.49 × 10^− 33^; Fig. 10c).

**Table 4:** Correlation analysis between neoantigen clonality and immune variables

Variable	*N*	Spearman ρ	*P*-value	Sig.
Neoantigen Count	505	0.134	0.003	**
TMB	505	0.178	5.97 × 10⁻⁵	***
Exhaustion Score	497	−0.412	9.76 × 10⁻²²	***
Core Exhaustion	497	−0.373	7.54 × 10⁻¹⁸	***
Pan-Immune Score	497	−0.500	8.49 × 10⁻³³	***
Immunosuppressive Gene Score (14-gene)	497	−0.41	1.9 × 10⁻²¹	***
Immune Suppression Score (Budhwani et al.)	497	−0.422	6.79 × 10⁻²³	***
CYT	495	−0.301	8.60 × 10⁻¹²	***
TIDE Dysfunction	498	−0.533	6.71 × 10⁻³⁸	***
TIDE Exclusion	498	0.235	1.19 × 10⁻⁷	***
Antigen Presentation	497	−0.312	1.05 × 10⁻¹²	***
MHC Class I	497	−0.240	6.10 × 10⁻⁸	***

This table presents Spearman correlation coefficients (ρ) between neoantigen clonality score and key immune-related variables across the HNSCC cohort. Clonality demonstrates positive correlations with mutational burden metrics (neoantigen count, TMB) but strong inverse correlations with all immune activity markers, including exhaustion, Pan-Immune Score, cytolytic activity, and TIDE dysfunction. The sole positive immune correlation is with TIDE exclusion, consistent with the interpretation that high clonality tumours exhibit an immunologically “cold” phenotype. Significance: ***P < 0.001, **P < 0.01

Individual exhaustion marker analysis revealed that 16 of 17 canonical exhaustion genes showed significant negative correlations with clonality (Fig. 10d; Table 5). The strongest associations were observed for IRF4 (ρ = −0.436, *P* = 1.59 × 10^− 24^), BTLA (ρ = −0.430, *P* = 8.28 × 10^− 24^), and TIM-3/HAVCR2 (ρ = −0.374, *P* = 6.70 × 10^− 18^). Only NR4A1 showed no significant correlation (ρ = 0.058, *P* = 0.20). Quartile analysis confirmed a stepwise decrease in exhaustion scores with increasing clonality (Q1 median = 2.22, Q2 = 1.77, Q3 = 1.64, Q4 = 1.33; Kruskal-Wallis H = 80.5, *P* = 2.4 × 10^− 17^; Fig. 10e).

**Table 5:** Individual T-cell exhaustion marker correlations with neoantigen clonality

Gene	Protein	ρ vs. Clonality	*P* (Clonality)	ρ vs. CYT	*P* (CYT)
IRF4	IRF4	−0.436	1.59 × 10⁻²⁴	0.607	3.04 × 10⁻⁵³
BTLA	BTLA	−0.430	8.28 × 10⁻²⁴	0.657	4.79 × 10⁻⁶⁵
HAVCR2	TIM-3	−0.374	6.70 × 10⁻¹⁸	0.727	5.88 × 10⁻⁸⁶
TIGIT	TIGIT	−0.369	1.76 × 10⁻¹⁷	0.838	8.65 × 10⁻¹³⁷
CD244	2B4	−0.357	1.99 × 10⁻¹⁶	0.729	1.77 × 10⁻⁸⁶
ENTPD1	CD39	−0.348	1.34 × 10⁻¹⁵	0.486	6.45 × 10⁻³²
TBX21	T-bet	−0.342	4.63 × 10⁻¹⁵	0.819	7.37 × 10⁻¹²⁶
PDCD1	PD-1	−0.332	3.12 × 10⁻¹⁴	0.867	4.07 × 10⁻¹⁵⁷
CTLA4	CTLA-4	−0.331	3.32 × 10⁻¹⁴	0.752	8.78 × 10⁻⁹⁵
BATF	BATF	−0.312	1.08 × 10⁻¹²	0.725	3.55 × 10⁻⁸⁵
TOX	TOX	−0.281	1.72 × 10⁻¹⁰	0.393	1.96 × 10⁻²⁰
EOMES	Eomes	−0.273	6.17 × 10⁻¹⁰	0.653	6.31 × 10⁻⁶⁴
PRDM1	Blimp-1	−0.254	9.91 × 10⁻⁹	0.136	0.002
LAG3	LAG-3	−0.240	6.18 × 10⁻⁸	0.866	3.35 × 10⁻¹⁵⁶
CD160	CD160	−0.216	1.22 × 10⁻⁶	0.403	1.55 × 10⁻²¹
TCF7	TCF-1	−0.215	1.25 × 10⁻⁶	0.319	1.12 × 10⁻¹³
NR4A1	NR4A1	0.058	0.20	−0.047	0.29

This table details Spearman correlations between individual exhaustion-associated genes and both neoantigen clonality and cytolytic activity (CYT). Of 17 canonical exhaustion markers examined, 16 showed significant negative correlations with clonality, with IRF4, BTLA, and TIM-3 (HAVCR2) demonstrating the strongest inverse associations. Conversely, all markers except NR4A1 showed strong positive correlations with CYT, confirming their validity as markers of immune-infiltrated tumours. The divergent correlation patterns highlight that exhaustion markers track with immune activity, not neoantigen clonality

#### Sensitivity analysis confirms robustness across alternative clonality definitions

To confirm that these associations are not artefacts of the specific clonality metric employed, we repeated all core analyses using four alternative clonality definitions: raw binder-weighted VAF, mean VAF per binder, binder-weighted VAF normalised by total neoantigen count, and log-transformed Clonality Score (Supplementary Table S5; Supplementary Figure S1A). The direction and significance of associations with all eight immune variables were reproduced across all five metrics (40 of 40 tests significant at *P* < 0.05), supporting the robustness of the observed clonality–immune relationship. Neoantigen binding-quality metrics (e.g., fraction of strong binders) did not show comparable associations (Supplementary Table S1), supporting specificity for clonality-related metrics. These results demonstrate that the inverse association between clonality and immune activity is robust to the specific computational definition employed and is not driven by idiosyncrasies of our primary metric.

#### Partial correlations confirm infiltration-mediated confounding with residual clonality-specific signal

To assess whether these inverse associations are driven by overall immune infiltration rather than clonality-specific biology, we computed partial Spearman correlations controlling for Pan-Immune Score (Supplementary Table S6). Most associations were substantially attenuated or reversed direction after adjustment: the Exhaustion Score partial correlation shifted from ρ = −0.412 to + 0.089 (*P* = 0.047), a negligible effect size representing a complete reversal of direction, Core Exhaustion from ρ = −0.373 to + 0.183 (*P* < 0.0001), and CYT from ρ = −0.301 to + 0.201 (*P* < 0.0001), confirming that the observed inverse correlations are largely mediated by differences in immune abundance. Notably, TIDE dysfunction retained a partial ρ = −0.233 (*P* < 0.0001), suggesting a residual clonality-specific effect on T-cell dysfunction scores beyond immune presence alone. Controlling for CD8 T-cell fraction alone barely altered the correlations (all remained negative with similar magnitude), indicating that confounding operates through the broader immune microenvironment rather than CD8 abundance specifically (Supplementary Figure S1B). To further assess whether tumour cellularity confounds the observed clonality–immune associations, we obtained ABSOLUTE-estimated tumour purity data from the TCGA PanCancer Atlas [30]. As expected given its VAF-based derivation, the Clonality Score correlated strongly with purity (ρ = 0.80, *P* < 10⁻¹⁰⁰). However, partial Spearman correlations controlling for purity preserved the inverse clonality–exhaustion association (partial ρ = −0.27, *P* < 0.0001), and further adjustment for both purity and Pan-Immune Score attenuated the association to non-significance (partial ρ = −0.02), consistent with infiltration-mediated confounding rather than a purity artefact.

#### Validation of clonality score against ABSOLUTE clonality estimates

To address whether the Clonality Score captures genuine tumour clonal architecture rather than simply reflecting mutation burden or tumour purity, we performed three validation analyses using independent metrics from the TCGA PanCancer Atlas.

First, we compared the Clonality Score against the ABSOLUTE-estimated Subclonal Genome Fraction (SGF), a copy-number-based metric derived from allele-specific copy number analysis that quantifies the fraction of the genome harbouring subclonal copy number alterations [30]. The Clonality Score correlated negatively with SGF (Spearman rho = -0.262, *P* = 3.95 × 10^-9, *n* = 488; Supplementary Figure S2A), confirming that higher Clonality Scores correspond to more clonal tumour architecture as assessed by an independent, non-VAF-based metric. Critically, ABSOLUTE SGF independently reproduced the key biological finding: more clonal tumours (lower SGF) exhibited significantly reduced CD8 + T-cell infiltration (rho = -0.165, *P* = 2.68 × 10^-4), lower cytolytic activity (rho = -0.096, *P* = 0.035), reduced IFN-gamma signalling (rho = -0.113, *P* = 0.013), and higher TIDE exclusion (rho = + 0.141, *P* = 0.002; Supplementary Table S9). These directionally concordant associations using a completely independent clonality metric provides orthogonal support for the interpretation that the inverse relationship between clonality and immune activity reflects tumour biology rather than being solely attributable to a methodological artefact of our VAF-based score.

Second, we confirmed that the Clonality Score captures distinct information from neoantigen burden. The correlation between Clonality Score and neoantigen count (neo_n_500) was weak (Spearman rho = 0.134, *P* = 0.003; Supplementary Figure S2B), demonstrating that these metrics are largely orthogonal. Importantly, neoantigen burden alone showed no significant association with most immune variables (Exhaustion Score: rho = -0.058, *P* = 0.20; CYT: rho = 0.014, *P* = 0.76; APM Score: rho = -0.042, *P* = 0.35), confirming that the immune associations observed with the Clonality Score are specific to the clonality dimension rather than reflecting mutation burden.

Third, partial Spearman correlations controlling for ABSOLUTE tumour purity confirmed that core immune associations were preserved after purity adjustment: Exhaustion Score (partial rho = -0.273, *P* = 1.2 × 10^-9), TIDE dysfunction (partial rho = -0.256, *P* = 1.3 × 10^-8), cytolytic activity (partial rho = -0.178, *P* = 9.0 × 10^-5), APM Score (partial rho = -0.115, *P* = 0.012), and IFN-gamma Score (partial rho = -0.192, *P* = 2.2 × 10^-5; Supplementary Table S9b). While attenuated compared to unadjusted correlations, these associations remained statistically significant, indicating that the clonality-immune relationship is not solely driven by tumour purity confounding.

#### IFN-gamma signalling and immune exclusion pathways

To investigate whether the clonality-immune disconnect involves impairment of IFN-gamma signalling, which is closely linked to antigen presentation, we correlated the Clonality Score with expression of ten IFN-gamma pathway genes. All ten genes showed significant negative correlations with clonality (Supplementary Table S10; Supplementary Figure S3A). The composite IFN-gamma Score correlated inversely with clonality (rho = -0.368, *P* = 2.2 × 10^-17), and this association remained significant after controlling for tumour purity (partial rho = -0.192, *P* = 2.2 × 10^-5). Among individual genes, the strongest associations were observed for CXCL9 (rho = -0.398, *P* = 2.7 × 10^-20), JAK2 (rho = -0.363, *P* = 6.2 × 10^-17), and JAK1 (rho = -0.360, *P* = 1.2 × 10^-16), all of which are critical for IFN-gamma-mediated upregulation of MHC class I molecules. These results suggest that high-clonality tumours exhibit attenuated IFN-gamma signalling, which may contribute to the observed reduction in antigen presentation machinery expression.

We further examined known immune exclusion pathways (Supplementary Table S10b; Supplementary Figure S3B). PTEN expression showed a significant negative correlation with clonality (rho = -0.304, *P* = 4.5 × 10^-12) and a positive correlation with TIDE dysfunction (rho = 0.244, *P* = 1.8 × 10^-8), consistent with the established role of PTEN loss in promoting immune exclusion. AXL, a marker of epithelial-mesenchymal transition (EMT)-mediated immune exclusion, also correlated negatively with clonality (rho = -0.275, *P* = 4.4 × 10^-10) and positively with TIDE dysfunction (rho = 0.426, *P* = 2.5 × 10^-24). By contrast, MAPK pathway genes (BRAF, KRAS, MAP2K1) showed no significant correlation with clonality (all |rho| < 0.04, *P* > 0.4), though their expression differed significantly across the four immune phenotypes (Kruskal-Wallis *P* < 0.01 for KRAS and MAP2K1). CTNNB1 (beta-catenin) showed a weak negative correlation with clonality (rho = -0.129, *P* = 0.004). These findings indicate that PTEN loss and EMT-associated exclusion programmes may contribute to the immune-cold phenotype observed in high-clonality tumours, while MAPK pathway activation shows phenotype-associated rather than clonality-associated variation.

#### Within-hot tumour analysis confirms clonality–immune association is not purely a hot/cold confounder

Within immune-hot tumours only (Pan-Immune Score ≥ median; *n* = 249), the inverse association between clonality and TIDE dysfunction was preserved (ρ = −0.417, *P* = 7.2 × 10⁻¹²; Supplementary Figure S1D), and 11 of 17 individual exhaustion-associated genes remained significantly negatively correlated with clonality (Supplementary Table S7; Supplementary Figure S1C). The strongest within-hot associations included BTLA (ρ = −0.265, *P* = 2.3 × 10⁻⁵), ENTPD1 (ρ = −0.255, *P* = 4.6 × 10⁻⁵), IRF4 (ρ = −0.243, *P* = 1.1 × 10⁻⁴), and TOX (ρ = −0.226, *P* = 3.2 × 10⁻⁴). These results demonstrate that the clonality–immune association is not solely an artefact of comparing hot versus cold tumours but reflects a graded relationship even within immunologically active tumours. In a multivariable regression adjusting for Pan-Immune Score and TMB (R² = 0.804, *n* = 497), clonality became a significant positive predictor of exhaustion (β = +0.52, SE = 0.20, *P* = 0.010; Supplementary Figure S1E), indicating that within tumours of comparable immune infiltration, higher clonality associates with greater T-cell engagement.

#### Immune phenotype stratification reveals four distinct tumour microenvironment states

**Fig. 11:**
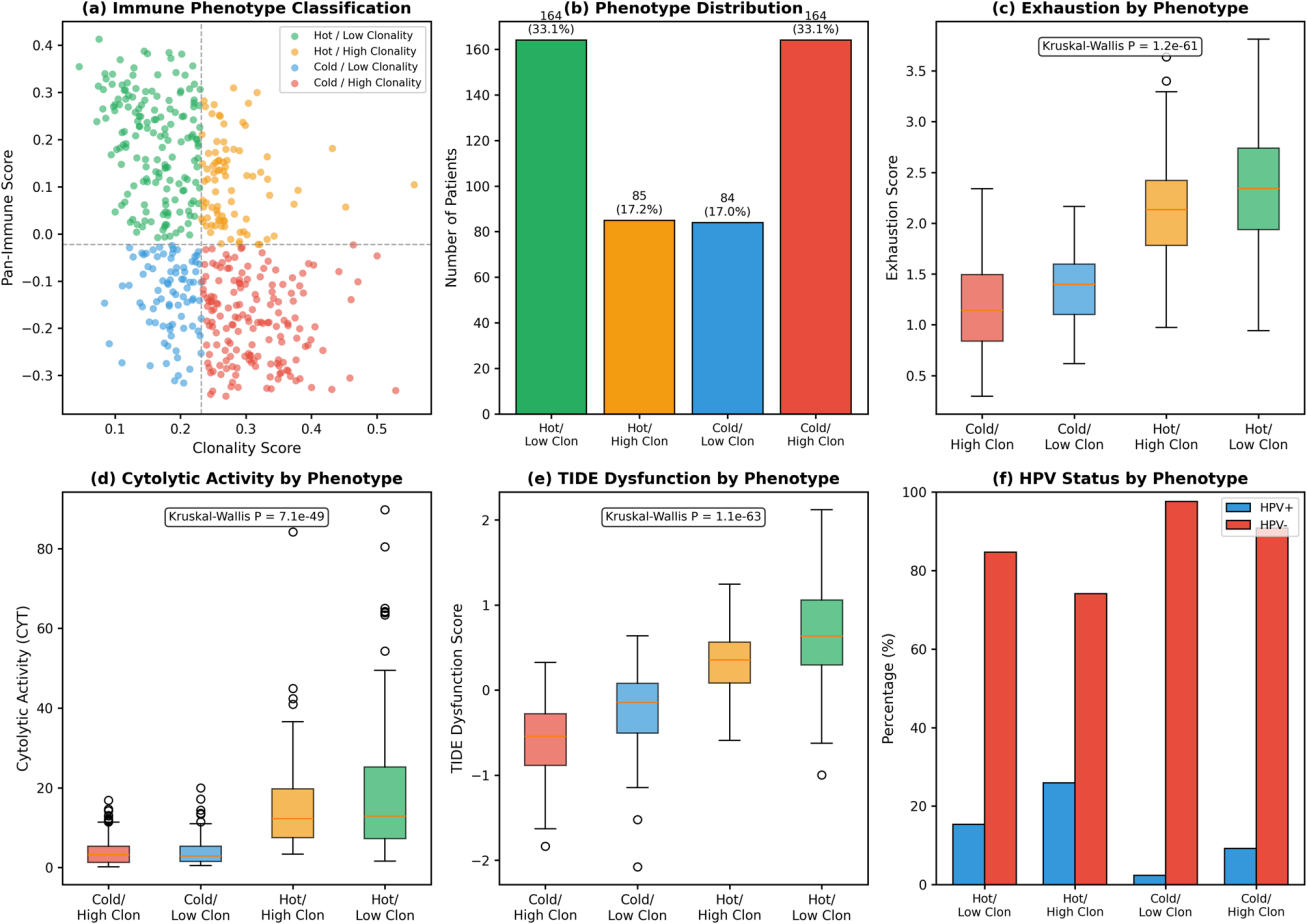
Immune phenotype stratification based on clonality and immune activity. **a**, Scatter plot of clonality score versus Pan-Immune Score with patients classified into four phenotypes based on median dichotomization: Hot/Low Clonality (green), Hot/High Clonality (orange), Cold/Low Clonality (blue), and Cold/High Clonality (red). Dashed lines indicate median thresholds. **b**, Bar chart showing distribution of patients across the four phenotypes with patient counts and percentages. **(c)** Box plots comparing exhaustion scores across phenotypes (Kruskal-Wallis *P* = 1.2 × 10^− 61^). **d**, Cytolytic activity (CYT) by phenotype (Kruskal-Wallis *P* = 7.1 × 10^− 49^). **e**, TIDE dysfunction score by phenotype (Kruskal-Wallis *P* = 1.1 × 10^− 63^). **f**, Stacked bar chart showing HPV status distribution within each phenotype. HPV status was significantly associated with phenotype distribution (χ² = 23.8, P = 2.72 × 10⁻⁵), with HPV-positive tumours enriched in immunologically ‘hot’ phenotypes

Based on median dichotomization of clonality and Pan-Immune scores, we classified tumours into four immune phenotypes (Fig. 11a-b). The cohort distributed as follows: Hot/Low Clonality (*n* = 164, 33.0%), Cold/High Clonality (*n* = 164, 33.0%), Hot/High Clonality (*n* = 85, 17.1%), and Cold/Low Clonality (*n* = 84, 16.9%). These phenotypes showed dramatically different immune characteristics (Fig. 11c-e; Table 6).

**Table 6:** Immune phenotype characteristics based on clonality and Pan-Immune Score stratification

Phenotype	*N*	% of Cohort	Clonality (median)	Exhaustion (median)	CYT (median)	TIDE Dysf (median)	HPV+ (%)
Hot / Low Clonality	164	33.1%	0.16	2.3	12.9	0.63	15.2%
Hot / High Clonality	85	17.2%	0.27	2.1	12.5	0.35	25.9%
Cold / Low Clonality	84	17.0%	0.19	1.4	2.9	-0.15	2.4%
Cold / High Clonality	164	33.1%	0.30	1.1	3.2	-0.54	9.8%

This table summarises the characteristics of four immune phenotypes defined by median dichotomization of clonality score and Pan-Immune Score. The Hot/Low Clonality phenotype exhibits the highest exhaustion, cytolytic activity, and TIDE dysfunction scores, representing “hot but exhausted” tumours potentially responsive to checkpoint inhibition. The Cold/High Clonality phenotype shows minimal immune activity despite abundant clonal neoantigens, suggesting immune evasion or exclusion mechanisms. HPV-positive tumours were significantly enriched in immunologically ‘hot’ phenotypes (χ² = 23.8, *P* = 2.72 × 10⁻⁵)

The Hot/Low Clonality phenotype exhibited the highest exhaustion scores (median = 2.3), cytolytic activity (CYT median = 12.9), and TIDE dysfunction (median = 0.63), consistent with an immunologically “hot” but exhausted tumour microenvironment. Conversely, the Cold/High Clonality phenotype displayed the lowest exhaustion (median = 1.1), minimal cytolytic activity (median = 3.2), and low TIDE dysfunction (median = − 0.54), representing an immunologically “cold” microenvironment despite high clonal neoantigen burden. These differences were highly significant across all immune parameters (Kruskal-Wallis *P* < 10^− 48^ for all comparisons). Notably, HPV status was significantly associated with immune phenotype distribution (χ² = 23.8, P = 2.72 × 10⁻⁵; Fig. 11f). HPV-positive tumours were strongly enriched in immunologically ‘hot’ phenotypes, comprising 25.9% of Hot/High Clonality and 15.2% of Hot/Low Clonality tumours, compared to only 9.8% of Cold/High Clonality and 2.4% of Cold/Low Clonality tumours. This pattern is consistent with the known immunogenicity of HPV-driven HNSCC, where viral oncoproteins (E6/E7) serve as foreign antigens that promote T-cell infiltration.

#### Validation of hot/cold classification using alternative immune scores

We tested whether the inverse Clonality Score–immune relationship and the resulting hot/cold classification were robust to the specific immune metric employed. Across eleven alternative immune readouts — spanning single T-cell marker genes (CD3D, GZMB, GNLY, NKG7), CIBERSORT CD8 fraction, cytolytic activity (CYT), an ESTIMATE-style composite immune score, the IFN-γ Composite Score, the Wolf LIexpression score, and the Immunologic Constant of Rejection (ICR) score — the Clonality Score showed significant negative correlations with 10 of 11 metrics (Supplementary Table S15; Supplementary Figure S5). The strongest associations were observed for the LIexpression score (rho = − 0.455, *P* = 1.9 × 10⁻²⁶), ESTIMATE-style score (rho = − 0.389, *P* = 1.8 × 10⁻¹⁹), the ICR score (rho = − 0.385, *P* = 9.5 × 10⁻¹⁹), and CD3D expression (rho = − 0.380, *P* = 1.6 × 10⁻¹⁸). Hot/cold classifications based on each alternative immune score were highly concordant with the primary TIDE-dysfunction-based classification, with concordance ranging from 67.7% to 79.8% (Cohen’s kappa 0.353–0.597 for transcriptomic scores), confirming that the hot/cold dichotomy is not specific to a single bulk-RNA-seq-derived metric. These results demonstrate that the inverse relationship between Clonality Score and immune infiltration is robust across diverse computational frameworks, and that the median-split hot/cold classification used throughout this work is well supported by independent immune-context measures.

#### Unsupervised clustering validates four-phenotype classification

To assess whether the four immune phenotypes identified by median dichotomization reflect natural data structure rather than arbitrary grouping, we performed unsupervised clustering analyses (Supplementary Table S13; Supplementary Figure S2C). K-means clustering with k = 4 yielded a silhouette score of 0.359 and an ARI of 0.496 when compared with the median-split classification, indicating moderate-to-good concordance. GMM with k = 4 produced an ARI of 0.402. The BIC favoured k = 2 (BIC = 2691.7), consistent with the primary biological axis being immune hot versus cold, while k = 4 provided the best Akaike Information Criterion (AIC; 2639.9), supporting the additional resolution provided by clonality stratification. Tertile-based stratification confirmed a monotonic relationship between clonality and immune activity: tumours in the lowest clonality tertile had the highest mean TIDE dysfunction (0.438) and cytolytic activity (CYT = 13.4), while the highest clonality tertile had the lowest values (-0.391 and 6.3, respectively). These analyses demonstrate that the four-phenotype model captures genuine biological heterogeneity, with the median-split classification providing a pragmatic approximation of the underlying continuous clonality-immune landscape.

#### Neoantigen clonality inversely associates with antigen presentation machinery

**Fig. 12:**
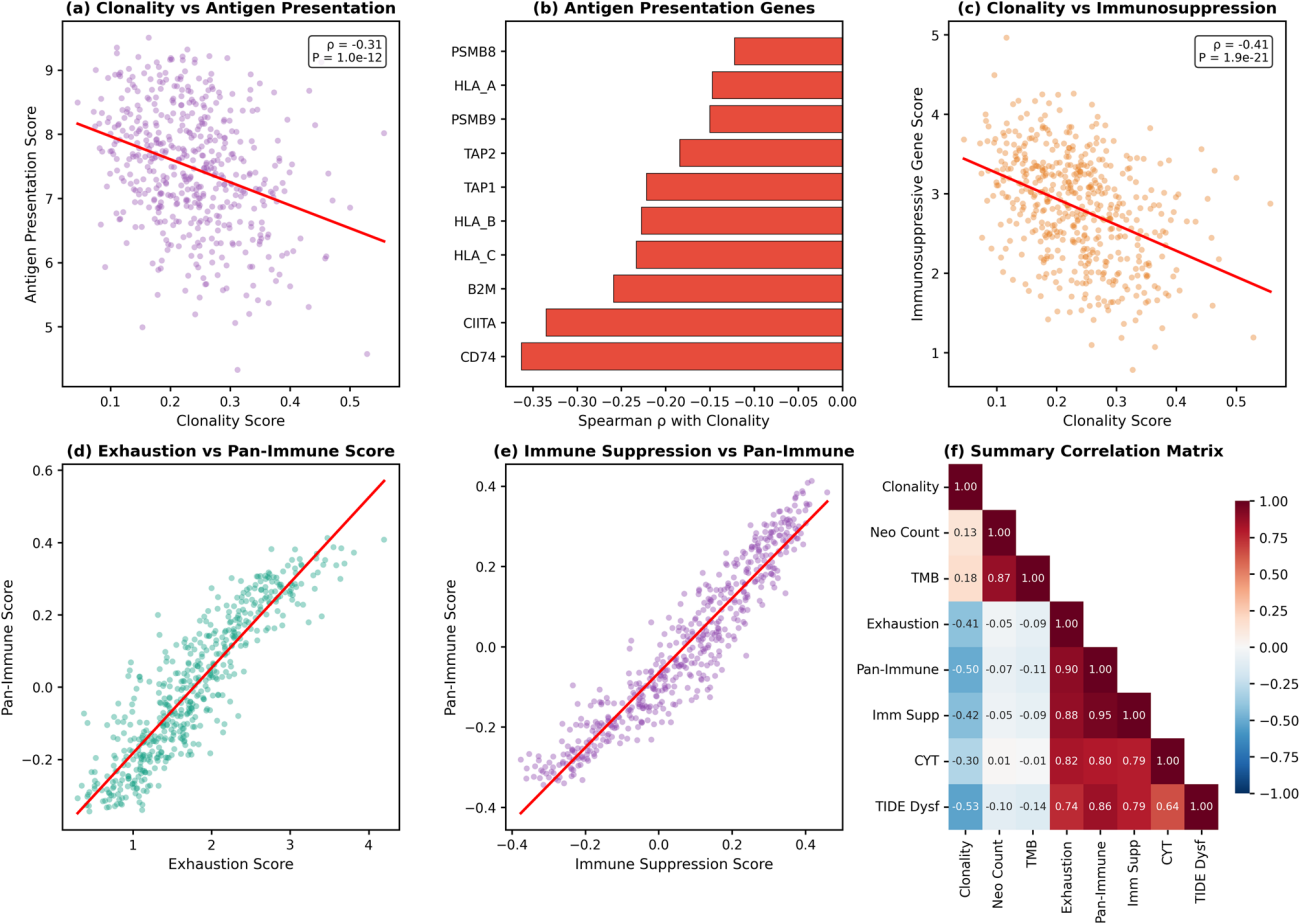
Antigen presentation, immunosuppression, and validation analyses **a**, Clonality score versus composite antigen presentation score (ρ = −0.31, *P* = 1.0 × 10^− 12^). **b**, Horizontal bar chart showing Spearman correlations between individual antigen presentation genes and clonality. All genes show negative correlations. **c**, Clonality versus 14-gene immunosuppressive gene score (ρ = −0.41, *P* = 1.9 × 10⁻²¹). **d**, Validation scatter plot showing strong positive correlation between our exhaustion score and the Pan-Immune Score from Budhwani et al. (ρ = 0.896). **e**, Exhaustion score versus Immune Suppression Score (ρ = 0.881), demonstrating co-occurrence of immune activity and immune suppression in “hot” tumours. **f**, Summary correlation matrix including clonality, neoantigen count, TMB, exhaustion, Pan-Immune Score, Immune Suppression Score, CYT, and TIDE dysfunction. Note the clustering of mutational burden metrics (positive inter-correlations) separate from immune metrics (negative correlations with clonality)

To investigate potential mechanisms underlying the clonality-immune paradox, we examined antigen presentation machinery (APM) gene expression. Clonality showed significant negative correlations with both the composite APM score (ρ = −0.312, *P* = 1.05 × 10^− 12^; Fig. 12a) and MHC Class I score (ρ = −0.240, *P* = 6.10 × 10^− 8^). Reduced APM expression would be expected to limit neoantigen visibility and impair T-cell priming upstream of any exhaustion process, potentially resulting in immune ignorance—a state in which the immune system fails to engage tumour antigens—rather than antigen-driven T-cell dysfunction. This interpretation positions impaired antigen presentation as a candidate upstream mechanism for the immunologically “cold” phenotype observed in high-clonality tumours. All examined APM genes demonstrated negative correlations with clonality, with CD74 (ρ = −0.34) and B2M (ρ = −0.19) showing the strongest and weakest effects, respectively (Fig. 12b).

Our 14-gene immunosuppressive signature inversely correlated with clonality (ρ = −0.41, *P* = 1.9 × 10⁻²¹; Fig. 12c). In bulk RNA-seq data, immunosuppressive gene expression often arises as a feedback response to immune activation and T-cell infiltration. Therefore, rather than indicating relief from immunosuppressive pressure, lower immunosuppressive signatures in high-clonality tumours more likely reflect limited immune activation and reduced immune cell abundance—consistent with an immune-absent rather than immune-permissive microenvironment. This was consistent with the Budhwani Immune Suppression Score (ρ = −0.42, *P* = 6.8 × 10⁻²³; Table 4). The summary correlation matrix (Fig. 12f) revealed a consistent pattern: clonality and traditional mutational burden metrics (TMB, neoantigen count) formed one correlation cluster, while all immune-related variables (exhaustion, Pan-Immune Score, CYT, TIDE dysfunction) formed a separate, tightly correlated cluster with opposite directionality to clonality. Our exhaustion signature demonstrated excellent concordance with previously established immune scores. The correlation with the Pan-Immune Score from Budhwani et al. [28] was exceptionally strong (ρ = 0.896, *P* = 5.05 × 10^− 184^; Fig. 12d), as was the correlation with their Immune Suppression Score (ρ = 0.881, *P* = 1.62 × 10^− 169^; Fig. 12e). This strong positive correlation between exhaustion and immune suppression scores is consistent with the “paradox” described by Budhwani et al. [28], wherein high immune activity and high immune suppression co-occur in immunologically “hot” tumours. Our findings extend this paradigm by demonstrating that neoantigen clonality inversely predicts this hot/exhausted phenotype, with high clonality tumours more likely to be immunologically “cold.”

#### Clonality-immune relationships are consistent across HPV strata

**Fig. 13:**
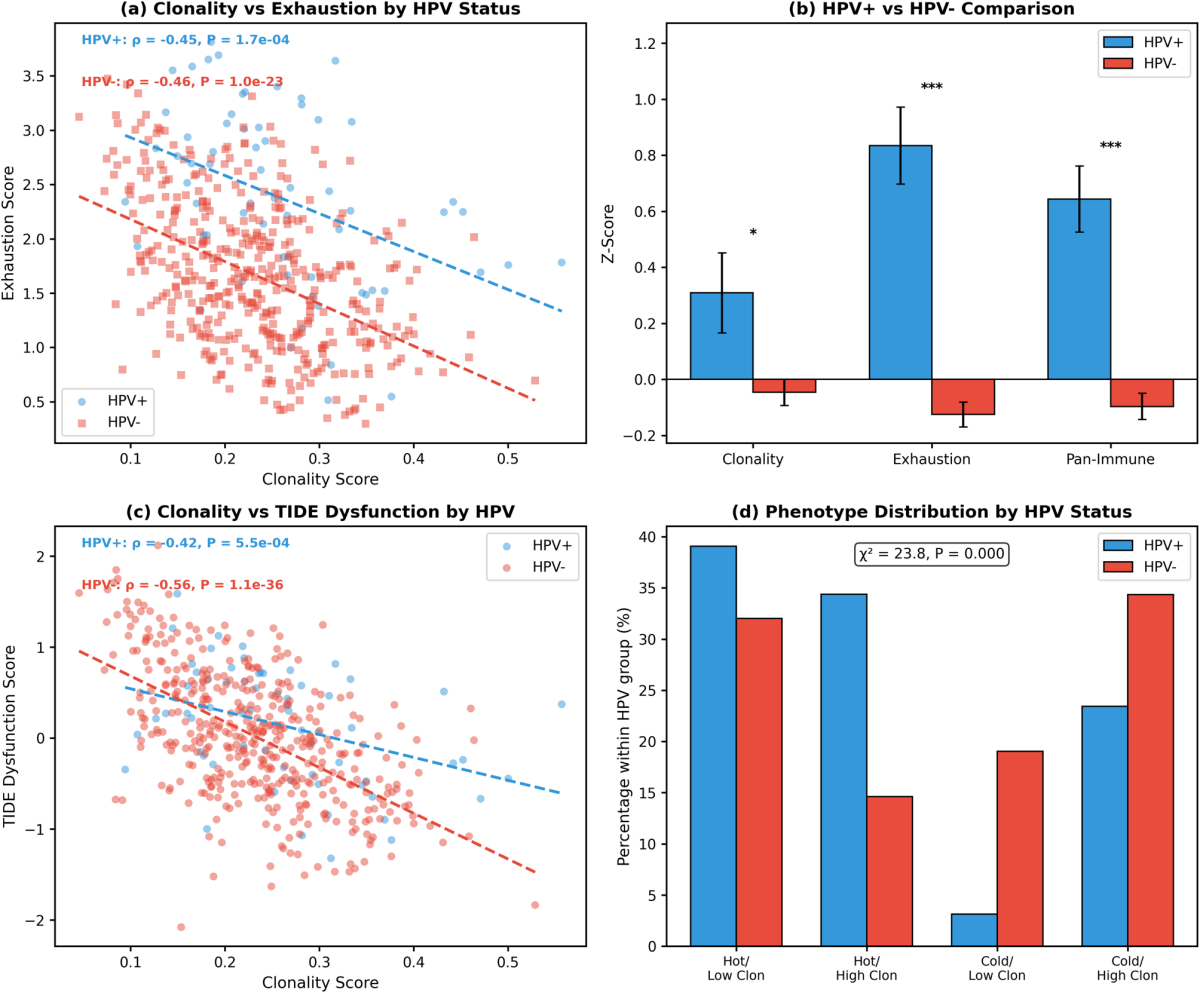
HPV-stratified analysis confirms robustness of clonality-immune relationships. **a**, Scatter plot of clonality versus exhaustion score stratified by HPV status. Both HPV-positive (ρ = −0.45, P = 1.7 × 10⁻⁴) and HPV-negative (ρ = −0.46, P = 1.0 × 10⁻²³) tumours show significant inverse correlations. **b**, Bar chart comparing Z-scores of clonality, exhaustion, and Pan-Immune Score between HPV-positive and HPV-negative tumours. No significant differences were observed (all P > 0.05). **c**, Clonality versus TIDE dysfunction by HPV status, showing strong inverse correlations in both groups (HPV+: ρ = −0.42; HPV−: ρ = −0.56). **d**, Phenotype distribution by HPV status showing significant association between HPV status and immune phenotype classification (χ² = 23.8, P = 2.72 × 10⁻⁵), with HPV-positive tumours enriched in ‘hot’ phenotypes

The inverse clonality-exhaustion relationship was preserved in both HPV-positive and HPV-negative subgroups (Fig. 13a, c; Supplementary Table S8). Both strata showed comparable correlation strengths: HPV-positive tumours (ρ = −0.449, P = 1.73 × 10⁻⁴, n = 65) and HPV-negative tumours (ρ = −0.457, P = 1.02 × 10⁻²³, n = 432), with both being highly significant. Similarly, TIDE dysfunction correlations were robust in both strata: HPV-positive (ρ = −0.417, P = 5.45 × 10⁻⁴) and HPV-negative (ρ = −0.558, P = 8.56 × 10⁻³⁷). While HPV status was significantly associated with immune phenotype distribution (Fig. 13d; χ² = 23.8, P = 2.72 × 10⁻⁵), with HPV-positive tumours enriched in immunologically ‘hot’ phenotypes, the clonality-immune relationship itself was consistent across both viral aetiologies. These findings suggest that the clonality-immune relationship represents a fundamental tumour biology principle that operates independently of the distinct immunogenic properties of HPV-driven carcinogenesis.

### Prognostic value of neoantigen clonality depends on immune context

Given the paradoxical inverse correlation between neoantigen clonality and T-cell infiltration, we investigated whether clonality carries prognostic significance and whether this association depends on immune context. We constructed a Cox proportional hazards model including neoantigen count (neo_n_500) and Clonality Score as separate predictors, along with TIDE dysfunction, TIDE exclusion, PD-L1 expression (CD274), age at diagnosis, and clinical stage.

In the main effects model (*n* = 386, 153 events), higher Clonality Score was associated with improved overall survival (HR = 0.79 per SD, 95% CI 0.64–0.98, *P* = 0.030), while neoantigen count showed no independent association (HR = 1.04, 95% CI 0.89–1.20, *P* = 0.64; Table 7). TIDE dysfunction was the strongest predictor (HR = 0.72, 95% CI 0.58–0.88, *P* = 0.002), confirming that immune infiltration is protective. TIDE exclusion was associated with worse survival (HR = 1.23, 95% CI 1.02–1.49, *P* = 0.030).

To test whether the prognostic effect of clonality depends on immune context, we included a Clonality Score × TIDE dysfunction interaction term. This interaction was statistically significant (HR = 0.85, 95% CI 0.72–0.99, P = 0.034), indicating that the association between clonality and survival differs between immunologically ‘hot’ and ‘cold’ tumours.

**Fig. 14:**
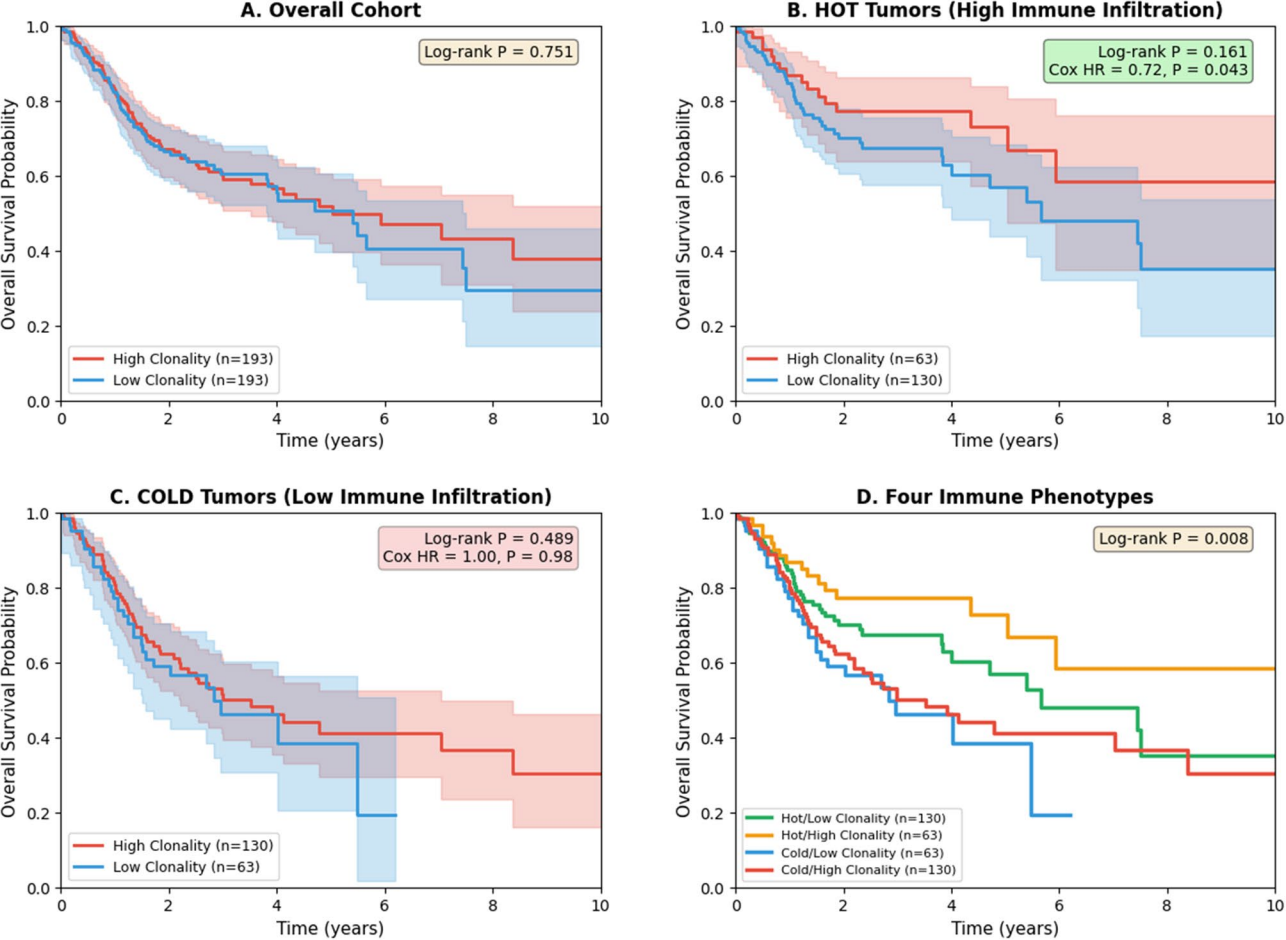
Survival analysis reveals immune context-dependent prognostic value of neoantigen clonality. (**a**) Kaplan–Meier curves for overall survival in the cohort stratified by median Clonality Score. No significant difference was observed (log-rank P = 0.751), reflecting the confounding influence of immune status. (**b**) In immunologically ‘hot’ tumours (TIDE dysfunction ≥ median, n = 193), high clonality was associated with improved survival (Cox HR = 0.72, 95% CI 0.53–0.99, P = 0.043), consistent with effective T-cell targeting of clonal neoantigens. (**c**) In ‘cold’ tumours (TIDE dysfunction < median, *n* = 193), clonality showed no prognostic association (Cox HR = 1.00, *P* = 0.98), indicating that neoantigen architecture is irrelevant in the absence of immune infiltration. (**d**) Kaplan–Meier analysis of the four immune phenotypes revealed significant survival differences (log-rank *P* = 0.008). Hot/High Clonality tumours showed the most favourable prognosis among immune-infiltrated tumours, while Cold/High Clonality tumours—despite abundant clonal neoantigens—showed poor outcomes due to immune exclusion. Cox HRs were adjusted for age at diagnosis and clinical stage; HPV-adjusted models yielded concordant results (Hot: HR = 0.71, *P* = 0.029; Cold: HR = 0.99, *P* = 0.96; Table 7)

Stratified analysis confirmed this finding (Fig. 14). In hot tumours (TIDE dysfunction ≥ median, *n* = 193), high clonality was associated with significantly improved survival after adjusting for age and clinical stage (HR = 0.72, 95% CI 0.53–0.99, *P* = 0.043). In striking contrast, clonality showed no prognostic association in cold tumours (TIDE dysfunction < median, *n* = 193; HR = 1.00, 95% CI 0.79–1.27, *P* = 0.98). Kaplan-Meier analysis of the four immune phenotypes revealed significant survival differences (log-rank *P* = 0.008), with Hot/High Clonality tumours demonstrating the most favourable prognosis among immune-infiltrated tumours. To rule out tumour cellularity as a confounder, we repeated the Cox model with ABSOLUTE-estimated purity as an additional covariate. Purity was not independently prognostic (HR = 1.12, *P* = 0.32), and the Exhaustion Score retained its significance (HR = 0.75 per SD, *P* < 0.005), confirming that the survival associations are not driven by differences in tumour cellularity.

These findings resolve the apparent paradox observed in our correlation analyses: while high clonality correlates with reduced immune infiltration at the population level, its prognostic benefit is realised only when T-cell effectors are present to recognise clonal neoantigens. This supports the hypothesis that clonal neoantigens represent optimal targets for T-cell-mediated tumour control, but only in the context of pre-existing anti-tumour immunity.

### Pairwise comparisons and categorical Cox regression of immune phenotypes

To formally identify which phenotype contrasts drive the overall survival difference observed in the four-group Kaplan–Meier analysis, we performed pairwise log-rank tests with Benjamini–Hochberg FDR correction (Supplementary Table S16; Supplementary Figure S6A; *n* = 476, 220 events). The global multivariate log-rank test confirmed significant heterogeneity (chi² = 14.48, *P* = 0.0023). Three pairwise comparisons remained statistically significant after FDR adjustment: Hot/High Clonality vs. Cold/High Clonality (chi² = 13.61, P_FDR = 0.0013), Hot/High Clonality vs. Cold/Low Clonality (chi² = 9.81, P_FDR = 0.0052), and Hot/Low Clonality vs. Hot/High Clonality (chi² = 5.23, P_FDR = 0.044). Comparisons among cold-phenotype groups and within hot/cold strata of similar clonality were non-significant after correction (P_FDR > 0.05). To corroborate these findings, we fitted a multivariable Cox proportional hazards model treating the four-phenotype variable as a categorical predictor with Hot/Low Clonality as the reference category, adjusting for age at diagnosis, advanced clinical stage, and HPV status (Supplementary Table S17; Supplementary Figure S6B; *n* = 405). Hot/High Clonality tumours exhibited significantly improved survival relative to Hot/Low Clonality (HR = 0.54, 95% CI 0.31–0.93, *P* = 0.026), whereas Cold/High Clonality (HR = 1.40, 95% CI 0.96–2.05, *P* = 0.080) and Cold/Low Clonality (HR = 1.31, 95% CI 0.82–2.08, *P* = 0.254) did not differ significantly from the reference. HPV status (HR = 1.10, *P* = 0.571) and advanced stage (HR = 1.36, *P* = 0.112) were not independently prognostic, while age remained a significant predictor (HR = 1.31, *P* = 0.002). These pairwise and categorical analyses formally demonstrate that the four-phenotype classification stratifies tumours into prognostically distinct subgroups, with the survival advantage concentrated specifically in tumours combining high immune infiltration with high neoantigen clonality. This pattern is consistent with the interaction model and supports the interpretation that clonal neoantigens confer survival benefit only when T-cell effectors are present to engage them.

#### Multivariable cox regression with HPV status as covariate

Given the strong association between HPV status and immune phenotype distribution, and the established prognostic importance of HPV in HNSCC, we repeated all survival models with HPV status as an additional covariate (Table 7; Supplementary Figure S2D). In the main effects model with HPV (*n* = 386, 153 events), the Clonality Score retained its prognostic significance (HR = 0.79, 95% CI 0.65–0.98, *P* = 0.030), TIDE dysfunction remained the strongest predictor (HR = 0.71, 95% CI 0.57–0.88, *P* = 0.002), and HPV status was not independently prognostic (HR = 1.12, 95% CI 0.79–1.59, *P* = 0.515). In the interaction model with HPV, the Clonality Score × TIDE dysfunction interaction remained statistically significant (HR = 0.84, 95% CI 0.71–0.98, *P* = 0.030), with HPV again non-significant (HR = 1.15, 95% CI 0.81–1.62, *P* = 0.434). In stratified analysis within hot tumours with HPV, age, and stage as covariates, clonality was significantly protective (HR = 0.71, 95% CI 0.52–0.97, *P* = 0.029), while HPV status had no independent effect (HR = 1.16, *P* = 0.588). In cold tumours, neither clonality (HR = 0.99, *P* = 0.958) nor HPV (HR = 1.12, *P* = 0.619) was prognostic. These results demonstrate that all prognostic associations are fully preserved after controlling for HPV status, and that immune context (hot vs. cold) rather than HPV status is the primary determinant of clonality-dependent survival benefit (Supplementary Figure S4).

**Table 7:** Multivariable Cox proportional hazards model for overall survival with interaction analysis

Variable	HR (95% CI)	*P*-value
*Main Effects Model* (*n = 386, events = 153)*		
Neoantigen count (neo_n_500)	1.04 (0.89–1.20)	0.64
Clonality Score	0.79 (0.64–0.98)	0.030*
TIDE dysfunction	0.72 (0.58–0.88)	0.002**
TIDE exclusion	1.23 (1.02–1.49)	0.030*
PD-L1 expression (CD274)	1.01 (0.87–1.17)	0.93
Age at diagnosis	1.30 (1.09–1.55)	0.003**
Advanced stage (III–IV)	1.32 (0.89–1.94)	0.16
*Interaction Model*		
Clonality Score × TIDE dysfunction	0.85 (0.72–0.99)	0.034*
*Stratified by Immune Status*		
Hot tumours (*n* = 193, events = 63): Clonality Score	0.72 (0.53–0.99)	0.043*
Cold tumours (*n* = 193, events = 90): Clonality Score	1.00 (0.79–1.27)	0.98
*HPV-Adjusted Main Effects Model (n* *= 386*,* events = 153)*		
Clonality Score	0.79 (0.65–0.98)	0.030*
TIDE dysfunction	0.71 (0.57–0.88)	0.002**
HPV positive	1.12 (0.79–1.59)	0.51
*HPV-Adjusted Interaction Model*		
Clonality Score × TIDE dysfunction	0.84 (0.71–0.98)	0.030*
HPV positive	1.15 (0.81–1.62)	0.43
*HPV-Adjusted Stratified by Immune Status*		
Hot tumours (*n* = 198): Clonality Score	0.71 (0.52–0.97)	0.029*
Hot tumours: HPV positive	1.16 (0.68–1.98)	0.59
Cold tumours (*n* = 188): Clonality Score	0.99 (0.78–1.26)	0.96
Cold tumours: HPV positive	1.12 (0.71–1.76)	0.62

This table presents hazard ratios (HR) with 95% confidence intervals from Cox regression models assessing the prognostic value of neoantigen clonality. The main effects model demonstrates that Clonality Score, but not neoantigen count, independently predicts survival. The significant interaction between Clonality Score and TIDE dysfunction (P = 0.034) indicates that the prognostic effect of clonality depends on immune context. Stratified analysis confirms that clonality is prognostically relevant only in immunologically ‘hot’ tumours harbouring T-cell infiltration. HPV-adjusted models confirm that all associations are preserved after controlling for HPV status, which is not independently prognostic. All continuous variables were z-standardised; hazard ratios represent the change in risk per one standard deviation increase. Hot tumours: TIDE dysfunction ≥ median (0.039); cold tumours: TIDE dysfunction < median. Stratified models were adjusted for age and clinical stage and HPV status. *P < 0.05, **P < 0.01

## Discussion

Our comprehensive analysis of HNSCC tumours reveals a paradoxical inverse relationship between neoantigen clonality and T-cell activity that challenges prevailing assumptions in tumour immunology. Contrary to the hypothesis that clonal neoantigens would drive T-cell exhaustion through persistent antigenic stimulation, we demonstrate that high-clonality tumours exhibit globally reduced immune infiltration, diminished immune-related transcriptional activity, and attenuated antigen presentation machinery expression—a pattern most consistent with immune ignorance or impaired immune engagement rather than persistent antigen-driven T-cell exhaustion. Importantly, survival analysis revealed that the prognostic value of clonality depends critically on immune context, with clonality predicting improved survival only in immune-infiltrated tumours. These findings have important implications for understanding immune evasion mechanisms and patient stratification for immunotherapy.

The seminal work by McGranahan and colleagues established that clonal neoantigens predict sensitivity to immune checkpoint blockade in lung cancer and melanoma, with T cells preferentially recognising antigens present in all tumour cells [9]. This paradigm has guided therapeutic development, with high clonal neoantigen burden considered a favourable biomarker for immunotherapy response [12, 31]. However, our data reveal that in HNSCC, clonality associates with the opposite phenotype: immunologically ‘cold’ tumours characterised by minimal T-cell infiltration despite abundant tumour-specific antigens. Crucially, our interaction analysis demonstrates that these observations are not contradictory: when T-cells are present, high clonality does indeed confer survival benefit (HR = 0.72), consistent with McGranahan’s findings [9]. The apparent paradox arises because high-clonality tumours have often already escaped immune surveillance and present as ‘cold’ tumours in treatment-naïve cohorts like TCGA.

Several non-mutually exclusive mechanisms may explain this paradox. First, high-clonality tumours may have undergone more extensive immunoediting, with early clonal evolution selecting for variants capable of escaping immune surveillance [13]. The cancer immunoediting hypothesis posits that the immune system shapes tumour evolution through three phases—elimination, equilibrium, and escape [32]. Tumours with predominantly clonal neoantigens may represent late-stage “escaped” tumours that have successfully evaded immune detection, whereas tumours with heterogeneous subclonal neoantigens may retain immunogenic subpopulations that continue to engage the immune system.

Second, our observation that high clonality correlates with reduced antigen presentation machinery (APM) expression provides a mechanistic link. Downregulation of MHC class I molecules, TAP transporters, and immunoproteasome components represents a well-characterised immune evasion strategy across multiple cancer types [33, 34]. The coordinated reduction in APM gene expression we observed suggests that high-clonality tumours may have undergone epigenetic or transcriptional reprogramming that simultaneously enriches for clonal variants while suppressing immune visibility. Indeed, loss of MHC-I expression occurs in 40–90% of human tumours and is associated with resistance to checkpoint blockade [35, 36].

Third, the inverse relationship between clonality and immune activity may reflect fundamentally different evolutionary trajectories. “Hot” tumours with active immune infiltration experience continuous selective pressure that generates subclonal diversity through ongoing immunoediting [10]. In contrast, “cold” tumours that evade immune recognition early in their evolution may expand clonally without the diversifying pressure of immune selection. This interpretation aligns with recent pan-cancer analyses demonstrating that immune-infiltrated tumours exhibit greater intratumoral heterogeneity than immune-excluded tumours [37].

Beyond these possibilities, additional non-mutually exclusive mechanisms may contribute to the clonality-immune disconnect. Tumour-intrinsic immune exclusion programmes—including activation of WNT/β-catenin signalling, loss of PTEN, or oncogenic MAPK pathway alterations—can actively prevent immune cell trafficking into the tumour microenvironment independently of antigen presentation [38, 39]. Stromal barriers, including dense extracellular matrix deposition and dysfunctional tumour vasculature, may physically prevent T-cell infiltration even when clonal neoantigens are abundant. Critically, the distinction between immune ignorance and immune evasion has important therapeutic implications: tumours exhibiting immune ignorance may harbour a naïve neoantigen-specific T-cell repertoire that has never been primed, whereas immune-evaded tumours may have already eliminated or exhausted neoantigen-reactive clones. Our data cannot definitively distinguish these scenarios; however, the reduced APM expression in high-clonality tumours (ρ = −0.31 with composite APM score) favours an immune ignorance model in which impaired antigen visibility prevents initial T-cell engagement. Recent work characterising the tumour immune macroenvironment using high-dimensional immune profiling has revealed that systemic immune dysfunction and plasticity of immune cells extend beyond the tumour microenvironment itself, underscoring the complexity of factors governing immune engagement in solid tumours [40]. Complementary single-cell and spatial transcriptomic analyses of colorectal cancer progression have demonstrated the dynamic enrichment of immunosuppressive regulatory T cells, myeloid subsets, and fibrotic stromal cells in advanced-stage tumours, alongside activation of cancer-associated regulatory hubs including the MAPK pathway, collectively contributing to impaired antitumour immunity — findings that reinforce the principle that tumour-intrinsic signalling programmes and microenvironmental remodelling can progressively suppress immune engagement independently of neoantigen availability [41].

Our analyses provide direct empirical support for the role of IFN-gamma signalling impairment in the clonality-immune disconnect. All ten IFN-gamma pathway genes examined showed significant negative correlations with clonality, with the composite IFN-gamma Score demonstrating a robust inverse association (rho = -0.368, *P* = 2.2 × 10^-17) that persisted after purity adjustment (partial rho = -0.192; Supplementary Table S10). Since IFN-gamma signalling is the principal upstream driver of MHC class I upregulation and antigen presentation, attenuated IFN-gamma activity in high-clonality tumours provides a mechanistic link between clonal enrichment and impaired antigen visibility. Furthermore, PTEN expression inversely correlated with clonality (rho = -0.304) and positively with immune infiltration, consistent with the established role of PTEN loss in T-cell exclusion across cancer types (Supplementary Table S10b; Supplementary Figure S3). These pathway-level findings strengthen the interpretation that the clonality-immune disconnect reflects a convergence of impaired antigen presentation and active immune exclusion programmes.

Our four-phenotype classification—Hot/Low Clonality, Hot/High Clonality, Cold/Low Clonality, and Cold/High Clonality—extends current frameworks for characterising the tumour immune microenvironment. The traditional dichotomy of “hot” versus “cold” tumours, based on T-cell infiltration and inflammatory signatures, has proven useful for predicting checkpoint inhibitor response [38, 39]. However, this classification does not capture the neoantigen landscape that ultimately determines immunogenicity.

The validity of the four-phenotype classification was further supported by unsupervised clustering analyses. K-means clustering with k = 4 achieved an ARI of 0.496 compared with the median-split classification, and BIC-based model selection from GMM identified k = 2 as the optimal fit, consistent with the primary biological axis being immune hot versus cold (Supplementary Table S13; Supplementary Figure S2C). Tertile-based stratification confirmed a monotonic gradient in immune activity across clonality levels, demonstrating that the four-phenotype model represents a pragmatic discretisation of a continuous biological relationship rather than an arbitrary grouping artefact.

Our survival analysis provides critical mechanistic insight into this paradox. While neoantigen clonality showed an overall protective effect (HR = 0.79, P = 0.030), we observed a significant interaction between clonality and immune status (P = 0.034). Stratified analysis revealed that high clonality conferred a survival benefit exclusively in immunologically ‘hot’ tumours (HR = 0.72, P = 0.043), whereas clonality had no prognostic value in ‘cold’ tumours (HR = 1.00, *P* = 0.98). This finding reconciles our correlation data with the McGranahan paradigm [9]: clonal neoantigens do indeed represent superior immunological targets, but their prognostic benefit is contingent upon pre-existing T-cell infiltration. In the absence of immune effectors, even optimal neoantigen architecture cannot confer survival advantage. This interaction effect has not been previously demonstrated in HNSCC and provides a biological explanation for the inconsistent prognostic value of neoantigen metrics reported across studies.

The largest phenotype groups in our cohort—Hot/Low Clonality (33%) and Cold/High Clonality (33%)—represent opposite ends of the immunological spectrum and likely require distinct therapeutic approaches. Hot/Low Clonality tumours exhibit the classical exhausted phenotype: abundant T-cell infiltration, high expression of checkpoint molecules (PD-1, TIM-3, LAG-3, CTLA-4), and elevated cytolytic activity. These tumours represent optimal candidates for checkpoint blockade monotherapy, as reinvigorating exhausted T cells should restore antitumour immunity [14].

Conversely, Cold/High Clonality tumours present a therapeutic challenge. Despite harbouring abundant clonal neoantigens—theoretically optimal targets for T-cell recognition—these tumours lack the immune infiltrate necessary for checkpoint inhibitor efficacy. This phenotype may explain why neoantigen burden alone inadequately predicts immunotherapy response, as the presence of antigens is insufficient without concurrent T-cell engagement [42]. For these patients, immune-priming strategies such as oncolytic viruses, STING agonists, or radiation therapy may be necessary to convert “cold” tumours to “hot” phenotypes before checkpoint blockade can be effective [43, 44]. Our survival data underscore this point: Cold/High Clonality tumours showed the poorest prognosis despite harbouring abundant clonal neoantigens, whereas Hot/High Clonality tumours—representing successful immune engagement with clonal targets—showed favourable outcomes. This suggests that therapeutic strategies for Cold/High Clonality patients should prioritise immune activation (e.g., oncolytic viruses, STING agonists, or radiation) before or concurrent with checkpoint inhibition, to convert these tumours to a ‘hot’ phenotype where their clonal neoantigens can become therapeutically relevant.

The strong negative correlation between clonality and our 17-gene exhaustion-associated signature (ρ = −0.41) requires careful interpretation given the limitations of bulk RNA-seq. T-cell exhaustion is a conditional functional state that is biologically meaningful only in the presence of infiltrating T cells [15, 16]. In bulk tumour transcriptomes, exhaustion-related gene expression reflects a composite of immune cell abundance and cellular functional state, and these components cannot be fully disentangled without single-cell or spatial resolution. Thus, reduced exhaustion signatures in high-clonality tumours most likely indicate diminished T-cell infiltration rather than altered exhaustion states per se—an interpretation consistent with the immune phenotype stratification showing that exhaustion scores closely track immune “hot” versus “cold” status (Fig. 11). We note that tumour purity and stromal composition may additionally influence bulk transcriptomic estimates of immune signatures; tumours with high purity (low stromal/immune content) would inherently show lower expression of immune-associated genes regardless of functional state. Future studies employing single-cell RNA sequencing or spatial transcriptomics will be essential to resolve whether the T cells that are present in high-clonality tumours exhibit qualitatively different exhaustion profiles compared to those in low-clonality tumours. Indeed, the multivariable regression presented revealed that after adjusting for Pan-Immune Score and TMB, clonality became a significant *positive* predictor of exhaustion (β = +0.52, *P* = 0.010; Supplementary Figure S1E). This reversal indicates that within tumours of comparable immune infiltration, higher clonality is associated with greater T-cell engagement—consistent with the original hypothesis that clonal neoantigens drive anti-tumour immune responses [9]. The paradoxical unadjusted inverse correlation is therefore explained by the strong association between high neoantigen clonality and immune-cold status (ρ = −0.50 with Pan-Immune Score), which overwhelms the within-context positive effect in unadjusted analyses. This finding provides a mechanistic reconciliation of our data with the McGranahan paradigm: clonal neoantigens do drive T-cell exhaustion through persistent engagement, but only when immune effectors are present; their population-level inverse correlation with exhaustion signatures reflects the preponderance of immune-cold tumours among high-clonality cases.

We further emphasise that genes such as PDCD1, CTLA4, LAG3, TIGIT, TOX, and other canonical exhaustion markers are predominantly expressed by infiltrating T cells, and their bulk-tissue abundance therefore primarily tracks T-cell infiltration density rather than per-cell exhaustion intensity. Lower expression of these markers in high-clonality tumours should accordingly be interpreted as evidence of reduced T-cell infiltration rather than as evidence that the few T cells that are present are functionally less exhausted. Throughout the manuscript, we have consistently reframed these findings in terms of immune infiltration, immune ignorance, and antigen visibility, rather than as direct evidence of differential per-cell exhaustion. Definitive resolution of per-cell exhaustion state will require single-cell or spatial transcriptomic profiling, which we explicitly identify as a critical future direction.

This interpretation is supported by the excellent correlation between our exhaustion score and established metrics of immune activity, including the Pan-Immune Score (ρ = 0.90) and cytolytic activity (ρ = 0.87 with GZMA/PRF1 expression). The co-occurrence of exhaustion signatures with immune suppression markers, as described by Budhwani and colleagues [28], reflects the “paradox” that immunologically active tumours exhibit both effector and suppressive features—a signature of ongoing immune engagement rather than dysfunction per se.

The persistence of clonality-immune relationships across HPV strata (HPV-positive: ρ = −0.45; HPV-negative: ρ = −0.46) demonstrates that this phenomenon represents fundamental tumour biology independent of viral aetiology. HPV-positive and HPV-negative HNSCC differ substantially in their mutational landscapes, immune microenvironments, and clinical outcomes [19, 45]. The consistency of our findings across these biologically distinct entities strengthens confidence in the generalisability of the clonality-immune relationship.

Our findings have direct implications for patient stratification and therapeutic development. First, neoantigen clonality may represent a candidate biomarker complementing existing predictors of checkpoint inhibitor response. Whereas high tumour mutational burden (TMB) and PD-L1 expression have been associated with response to pembrolizumab or nivolumab [5, 6], our data suggest that clonality provides orthogonal information about the tumour immune microenvironment. However, we emphasise that all analyses were performed in silico using treatment-naïve TCGA cohorts, and no direct association with immune checkpoint inhibitor response was evaluated. This biomarker hypothesis therefore requires prospective validation in immunotherapy-treated HNSCC cohorts before clinical implementation.

Second, the Cold/High Clonality phenotype identifies patients who may benefit from combination approaches targeting both immune priming and checkpoint blockade. Personalised neoantigen vaccines represent a particularly attractive strategy for these patients, as they can prime T-cell responses against tumour-specific antigens that are already clonally expressed [8, 46]. Recent clinical trials of personalised neoantigen vaccines in HNSCC, including TG4050, have demonstrated the ability to induce neoantigen-specific T-cell responses and preliminary evidence of clinical benefit [47, 48]. Based on the immune phenotype characterisation presented here, we hypothesise that patients with Cold/High Clonality tumours—those with abundant clonal antigens but absent immune infiltration—may represent a population for whom such immune-priming approaches could be explored. This remains a hypothesis-generating observation that requires testing in prospective clinical studies. Beyond tumour-antigen–directed approaches, emerging evidence suggests that mRNA vaccination may augment responsiveness to checkpoint blockade by inducing innate immune activation and upregulating PD-L1 in otherwise immunologically cold tumour [49, 50]. Although these observations are retrospective and require prospective validation, they are consistent with our central model that immune priming may be a prerequisite for exploiting clonal neoantigens in immune-excluded tumours (47,48).

Third, our HLA supertype analysis reveals that HLA-C-restricted peptides dominate the public neoantigen landscape in HNSCC. This finding has implications for “off-the-shelf” vaccine development, as targeting HLA-C supertypes (particularly C07 and C03) could provide broader population coverage than traditional HLA-A/B-focused approaches. The correlation between C15-restricted public peptides and immune markers (PD-L1, cytolytic activity) suggests that certain HLA contexts may be particularly favourable for neoantigen immunogenicity.

This study has several strengths. The large sample size (*n* = 527) and comprehensive integration of genomic, transcriptomic, and clinical data from TCGA provide robust statistical power. Our clonality metric, which normalises binder-weighted VAF by neoantigen count, isolates the effect of clonality independent of overall burden—addressing a limitation of prior studies that conflated these properties. We acknowledge that alternative clonality definitions—such as PyClone-based clonal cluster assignments or cancer cell fraction estimates—may yield different quantitative results, and our findings should be interpreted in the context of the specific metric employed. Reassuringly, the consistent direction and strength of correlations across multiple orthogonal immune readouts (TIDE dysfunction, Pan-Immune Score, cytolytic activity, individual exhaustion markers) suggest that the observed associations are robust to the specific metric used. Indeed, a formal sensitivity analysis using four alternative clonality definitions confirmed that all core associations with immune variables were reproduced across all five metrics (40 of 40 tests significant; Supplementary Table S5; Supplementary Figure S1A), and partial correlations controlling for Pan-Immune Score demonstrated that most associations are infiltration-mediated while TIDE dysfunction retains a residual clonality-specific signal (Supplementary Table S6). Validation against established immune scores (Pan-Immune Score, TIDE) confirms the biological relevance of our findings, and HPV stratification demonstrates robustness across aetiological subgroups. From a biomarker perspective, our findings caution against using neoantigen clonality in isolation for patient stratification. The significant clonality × immune status interaction (*P* = 0.034) demonstrates that clonality metrics must be interpreted alongside measures of immune infiltration. A patient with high clonality and high immune infiltration may be more likely to benefit from checkpoint blockade, whereas a patient with identical clonality but low immune infiltration may require combination approaches. This two-dimensional stratification—combining neoantigen quality with immune context—may improve predictive accuracy compared to single-parameter biomarkers.

Importantly, we validated the Clonality Score against ABSOLUTE-estimated Subclonal Genome Fraction, a copy-number-based clonality metric that is entirely independent of our VAF-based approach. The significant negative correlation (rho = -0.262, *P* = 3.95 × 10^-9; Supplementary Figure S2A) supports that the Clonality Score captures biologically meaningful variation related to tumour clonality, while remaining an imperfect VAF-weighted proxy rather than a direct CCF-based measure of clonal architecture. Moreover, the Subclonal Genome Fraction independently reproduced the key biological finding that more clonal tumours exhibit reduced CD8 + T-cell infiltration (rho = -0.165, *P* = 2.68 × 10^-4) and lower cytolytic activity (rho = -0.096, *P* = 0.035; Supplementary Table S9), providing orthogonal support for the conclusion that the inverse clonality-immune relationship is not solely driven by the specific clonality metric employed. We further demonstrated that the Clonality Score is weakly correlated with neoantigen burden (rho = 0.134; Supplementary Figure S2B), confirming that it captures the clonality dimension specifically rather than serving as a proxy for mutation load.

We further emphasise that the term ‘Clonality Score’ should be interpreted with appropriate caution. Because the metric is constructed from variant allele frequencies, it inherits sensitivity to tumour purity, copy-number state, ploidy, and local loss of heterozygosity, and it is therefore more accurately described as a VAF-weighted neoantigen clonality proxy rather than a direct measurement of cancer cell fraction. The convergence of evidence from the ABSOLUTE Subclonal Genome Fraction validation (Supplementary Table S9), the partial correlations controlling for purity (Supplementary Table S9b), the multiple alternative metric formulations (Supplementary Table S5), and the alternative immune-score validations (Supplementary Table S15) collectively supports the conclusion that the inverse clonality–immune relationship reflects a real biological pattern that is robust across complementary computational definitions, even though no individual VAF-based metric perfectly captures true clonal architecture.

However, several limitations warrant consideration. First, as a retrospective in silico analysis of treatment-naïve TCGA data, we lack prospective validation in immunotherapy-treated cohorts. No direct association between clonality and response to immune checkpoint blockade was evaluated, and all therapeutic implications therefore remain hypothesis-generating. Whether clonality predicts checkpoint inhibitor response in HNSCC requires confirmation in clinical trial datasets. Second, bulk RNA sequencing cannot fully distinguish between reduced immune cell presence and altered functional states. In particular, T-cell exhaustion is a conditional functional state that is biologically meaningful only when T cells are infiltrating the tumour; in immune-cold tumours, reduced exhaustion signatures may reflect immune absence rather than functional competence. Tumour purity and stromal composition further confound bulk transcriptomic estimates of immune cell activity. Validation using single-cell RNA sequencing or spatial transcriptomic datasets would substantially strengthen the biological interpretation by resolving whether the rare T cells in high-clonality tumours differ qualitatively in exhaustion state from those in low-clonality tumours. Third, our neoantigen predictions rely on computational algorithms (pVACseq, MHCflurry) with imperfect accuracy; experimental validation of predicted neoepitopes would strengthen conclusions about immunogenicity. Fourth, the observational design precludes causal inference; interventional studies are needed to determine whether modulating clonality or immune infiltration affects therapeutic outcomes. Finally, TCGA samples represent primary untreated tumours; the clonality-immune relationship may differ in recurrent or metastatic disease following prior therapy.

Fifth, our Clonality Score is based on variant allele frequency and is therefore influenced by tumour purity and copy number state. While we validated the score against ABSOLUTE Subclonal Genome Fraction (Supplementary Table S9; Supplementary Figure S2A) and demonstrated that core associations are preserved after purity adjustment (Supplementary Table S9b), per-variant cancer cell fraction estimation using tools such as PyClone was not feasible at cohort scale. Future studies with access to allele-specific copy number calls at each variant locus could refine the clonality metric and potentially strengthen the observed associations. Sixth, the four-phenotype classification relies on median dichotomization, which inherently simplifies continuous distributions. Although unsupervised clustering and tertile-based analyses supported the biological validity of this classification (Supplementary Table S13; Supplementary Figure S2C), continuous or data-driven phenotyping approaches may capture additional nuance in larger validation cohorts. Seventh, the term ‘Clonality Score’ reflects a VAF-weighted neoantigen clonality proxy rather than a direct measure of true tumour clonal architecture, and inherits sensitivity to tumour purity, ploidy, copy-number state, and local LOH. We have therefore explicitly framed this metric as a proxy throughout the manuscript and validated it against the orthogonal, copy-number-based ABSOLUTE Subclonal Genome Fraction. Eighth, individual exhaustion marker genes in bulk RNA-seq predominantly track T-cell infiltration density rather than per-cell exhaustion intensity. Lower expression of PDCD1, CTLA4, LAG3, TIGIT, and TOX in high-clonality tumours should accordingly be interpreted as evidence of reduced T-cell infiltration, not as evidence that the rare T cells in those tumours are less functionally exhausted; definitive resolution will require single-cell or spatial profiling.

## Conclusions

We demonstrate that neoantigen clonality inversely predicts T-cell activity in HNSCC, challenging the assumption that clonal neoantigens uniformly promote antitumour immunity. High-clonality tumours exhibit an immunologically “cold” phenotype characterised by reduced immune infiltration and attenuated antigen presentation, suggesting that clonal enrichment may reflect immune ignorance or impaired immune engagement rather than enhanced immunogenicity. Crucially, survival analysis revealed that the prognostic benefit of neoantigen clonality is context-dependent: high clonality predicted improved survival only in immunologically ‘hot’ tumours (HR = 0.72, P = 0.043), whereas clonality had no prognostic effect in ‘cold’ tumours (HR = 1.00, *P* = 0.98). This finding reconciles our observations with the paradigm that clonal neoantigens represent optimal immunological targets, but only when T-cell effectors are present. Our four-phenotype classification—integrating clonality and immune status—provides a framework for patient stratification that may guide therapeutic decision-making. We hypothesise that patients with Cold/High Clonality tumours may benefit from immune-priming strategies (vaccines, oncolytic viruses, STING agonists) prior to checkpoint inhibition, whereas Hot/Low Clonality patients may be candidates for checkpoint blockade alone; however, these stratification strategies require prospective validation in immunotherapy-treated cohorts. These findings underscore the complexity of tumour-immune interactions and highlight the need for multidimensional biomarker approaches in precision immuno-oncology. Taken together, our findings indicate that neoantigen clonality represents a context-dependent biomarker whose therapeutic relevance is unlocked only in the presence of effective immune infiltration. The observation that high-clonality tumours frequently exhibit an immune-excluded phenotype suggests that immune priming may be a prerequisite for exploiting clonal neoantigens as immunological targets. In this context, recent mechanistic and clinical evidence that mRNA vaccination can induce innate immune activation and enhance responsiveness to immune checkpoint inhibition provides a compelling proof-of-concept that immune-priming strategies warrant prospective evaluation. Although prospective validation is required, these data collectively support a model in which converting Cold/High Clonality tumours into an immune-infiltrated state may be necessary to realise the prognostic and therapeutic potential of clonal neoantigens.

## Supplementary Information

Below is the link to the electronic supplementary material.

Supplementary Material 1

Supplementary Material 2

Supplementary Material 3

Supplementary Material 4

Supplementary Material 5

Supplementary Material 6

Supplementary Material 7

Supplementary Material 8

Supplementary Material 9

Supplementary Material 10

Supplementary Material 11

Supplementary Material 12

Supplementary Material 13

Supplementary Material 14

Supplementary Material 15

Supplementary Material 16

Supplementary Material 17

Supplementary Material 18

Supplementary Material 19

Supplementary Material 20

Supplementary Material 21

Supplementary Material 22

Supplementary Material 23

Supplementary Material 24

## Data Availability

All data generated or analyzed during the study are included in the paper or its online supplemental information, or available upon request. Due to TCGA privacy concerns, the HLA allele data is provided only to researchers who have access to the primary sequence data from the TCGA. Analysis code, including all custom Python scripts used for neoantigen summarisation, Clonality Score computation, immune score calculation, partial correlation analyses, unsupervised clustering, pairwise log-rank tests, and Cox regression, is deposited at https://github.com/Freiburg-AI-Research/HNSCC_neoantigen_clonality, together with the processed sample-level analytical table linking TCGA barcodes to all computed neoantigen, immune, and clonality features (provided as Supplementary Data S1; *n* = 527 tumours; full variable definitions in the accompanying README). Detailed pVACseq parameters (binding affinity predictor MHCflurry v2.1.5; peptide lengths 8–11 amino acids; binder threshold IC50 ≤ 500 nM; strong binder threshold IC50 ≤ 50 nM), HLA typing consensus calls (POLYSOLVER, OptiType, xHLA, HLA-HD, hla-genotyper, SOAP-HLA, HLA-VBSeq, Kourami), and full gene lists for the 17-gene exhaustion panel, the 17-gene antigen-presentation machinery panel, the 14-gene immunosuppression panel, and the 10-gene IFN-γ pathway panel are reported in Methods.
