# Supplementary material for: Inverse relationship between neoantigen clonality and T-cell activity reveals distinct immune phenotypes in HNSCC
Source: J Transl Med. 2026 Jun 3;24:731. doi: 10.1186/s12967-026-08371-z (PMC13235206; doi:10.1186/s12967-026-08371-z)

**Supplementary Figure S1 | Robustness and Mechanistic Analyses.**

Multi-panel analysis supporting the robustness and interpretation of the clonality–immune relationship. **(A)** Sensitivity heatmap: Spearman correlations across five alternative clonality metrics and eight immune variables; four of five metrics replicate all significant associations. **(B)** Partial correlation comparison: bivariate correlations (blue) are substantially attenuated when controlling for Pan-Immune Score (red) but unchanged when controlling for CD8 fraction alone (green), indicating confounding through the broader immune microenvironment. **(C)** Within-hot tumour analysis: individual exhaustion gene correlations with clonality among immune-hot tumours (n = 249); red bars denote P < 0.05. **(D)** TIDE dysfunction versus clonality stratified by immune status, with regression lines; the inverse relationship is preserved within both hot and cold strata. **(E)** Residual exhaustion score (after regressing on Pan-Immune Score and TMB) versus Clonality Score, showing a positive relationship (β = +0.52, P = 0.010) indicating that within tumours of comparable immune infiltration, higher clonality predicts greater T-cell engagement.


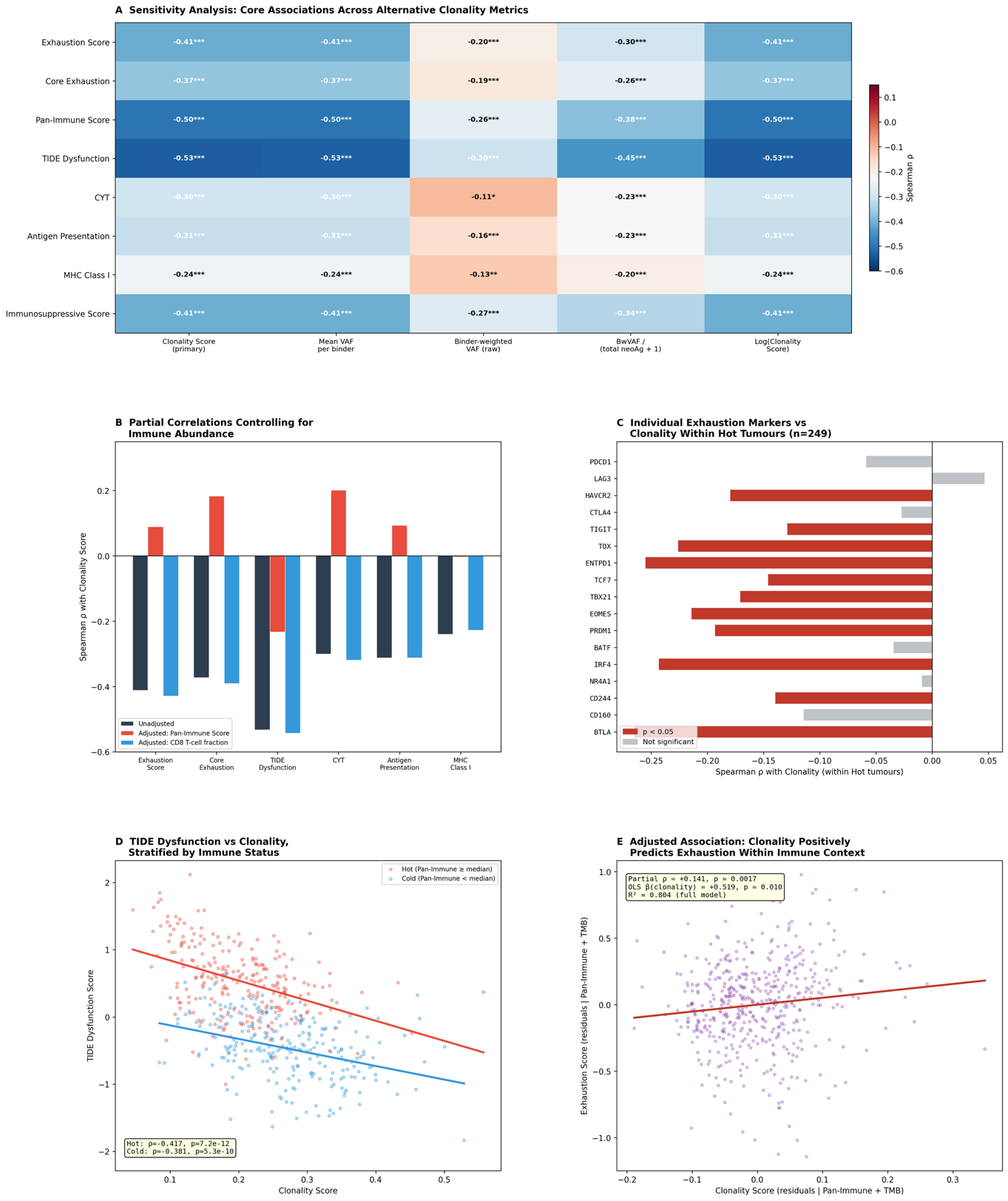

Supplement: Supplementary file 1 — Supplementary Material 1 [file 12967_2026_8371_MOESM1_ESM.docx]
