# Supplementary material for: Inverse relationship between neoantigen clonality and T-cell activity reveals distinct immune phenotypes in HNSCC
Source: J Transl Med. 2026 Jun 3;24:731. doi: 10.1186/s12967-026-08371-z (PMC13235206; doi:10.1186/s12967-026-08371-z)

**Supplementary Figure S2 | Clonality Score validation, burden orthogonality, clustering, and HPV-adjusted survival.**

(A) Scatter plot of Clonality Score versus ABSOLUTE-estimated Subclonal Genome Fraction (SGF). The significant negative correlation (Spearman ρ = −0.262, P = 3.95 × 10⁻⁹, n = 488) confirms that higher Clonality Scores correspond to more clonal tumour architecture as assessed by an independent, copy-number-based metric. Red line indicates linear fit. (B) Scatter plot of Clonality Score versus neoantigen burden (neo_n_500). The weak correlation (ρ = 0.134, P = 0.003) demonstrates that the Clonality Score captures distinct information from mutation burden, confirming it is not a proxy for neoantigen count. (C) K-means clustering (k = 4) of z-standardised Clonality Score and TIDE dysfunction values. Colours indicate unsupervised cluster assignments; dashed grey lines indicate median-split thresholds used for the four-phenotype classification. The Adjusted Rand Index (ARI) of 0.496 indicates moderate-to-good concordance between unsupervised clusters and median-split phenotypes, supporting the biological validity of the four-phenotype model. Silhouette score = 0.359. (D) Forest plot of hazard ratios from the multivariable Cox proportional hazards model including Clonality Score (z-standardised), immune status (hot/cold), their interaction term, and HPV status as covariates (n = 386, 153 events). Immune hot status is the strongest predictor of improved survival (HR = 0.54, P = 0.0001). HPV status is not independently prognostic (HR = 1.01, P = 0.96). Error bars represent 95% confidence intervals; dashed vertical line indicates HR = 1 (no effect).


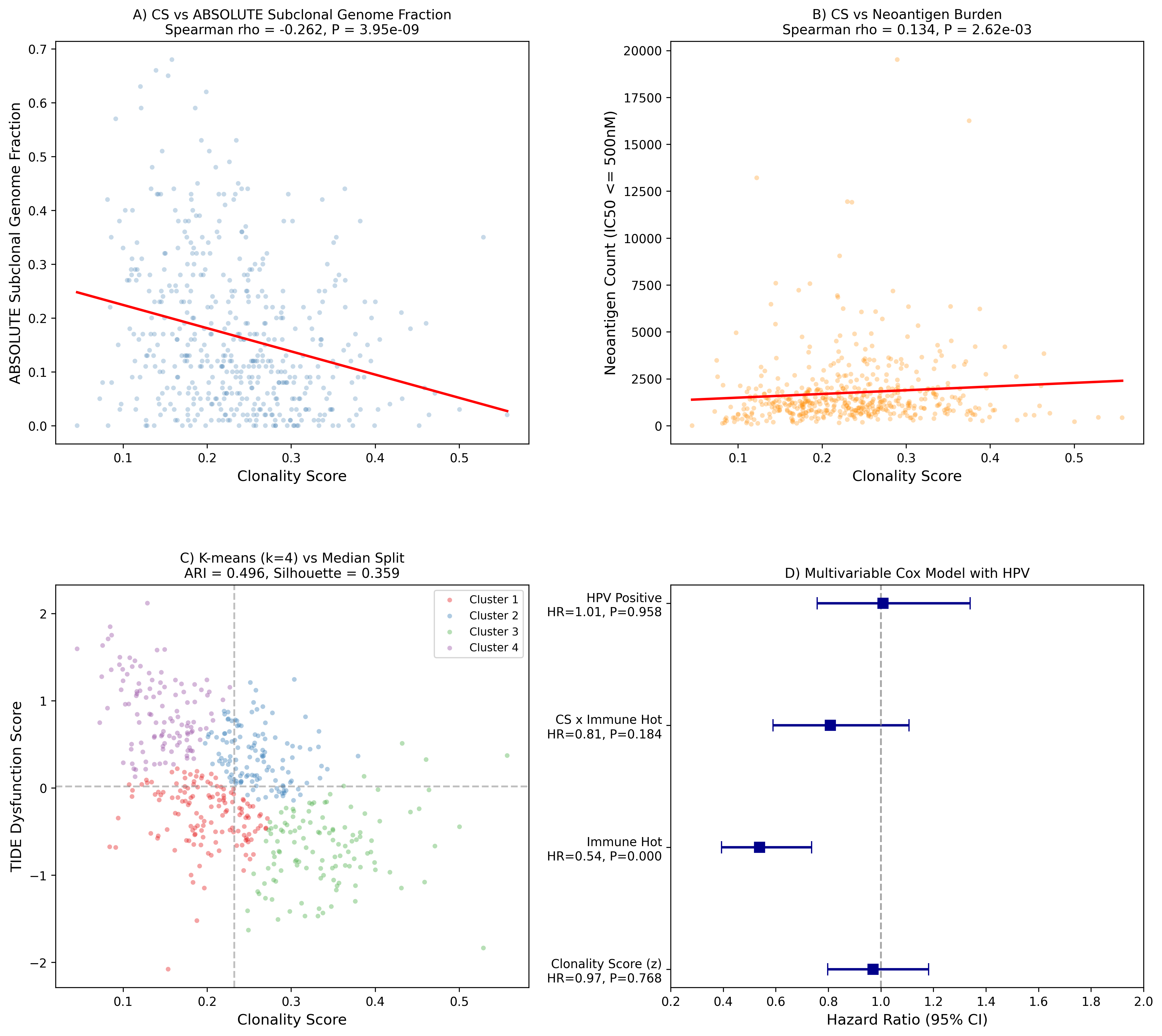

Supplement: Supplementary file 2 — Supplementary Material 2 [file 12967_2026_8371_MOESM2_ESM.docx]
