# Supplementary material for: Inverse relationship between neoantigen clonality and T-cell activity reveals distinct immune phenotypes in HNSCC
Source: J Transl Med. 2026 Jun 3;24:731. doi: 10.1186/s12967-026-08371-z (PMC13235206; doi:10.1186/s12967-026-08371-z)

**Supplementary Figure S3 | IFN-γ signalling and immune exclusion pathway correlations with Clonality Score.**

(A) Horizontal bar chart showing Spearman correlation coefficients between individual IFN-γ pathway genes and the Clonality Score. All ten genes show significant negative correlations, with CXCL9 (ρ = −0.398), JAK2 (ρ = −0.363), and JAK1 (ρ = −0.360) demonstrating the strongest associations. These genes are critical for IFN-γ-mediated upregulation of MHC class I molecules, suggesting that attenuated IFN-γ signalling in high-clonality tumours may contribute to reduced antigen presentation. Significance: ***P < 0.001, **P < 0.01, *P < 0.05, ns = not significant. Bar colour intensity reflects significance level. (B) Horizontal bar chart showing Spearman correlations between immune exclusion pathway genes and the Clonality Score. PTEN (ρ = −0.304, P = 4.5 × 10⁻¹²) and AXL (ρ = −0.275, P = 4.4 × 10⁻¹⁰) show significant negative correlations, consistent with roles of PTEN loss and epithelial-mesenchymal transition (EMT) in immune exclusion. MAPK pathway genes (BRAF, KRAS, MAP2K1) show no significant direct correlation with clonality. CTNNB1 and WNT5A show weak negative correlations (ρ = −0.129 and −0.127, respectively).


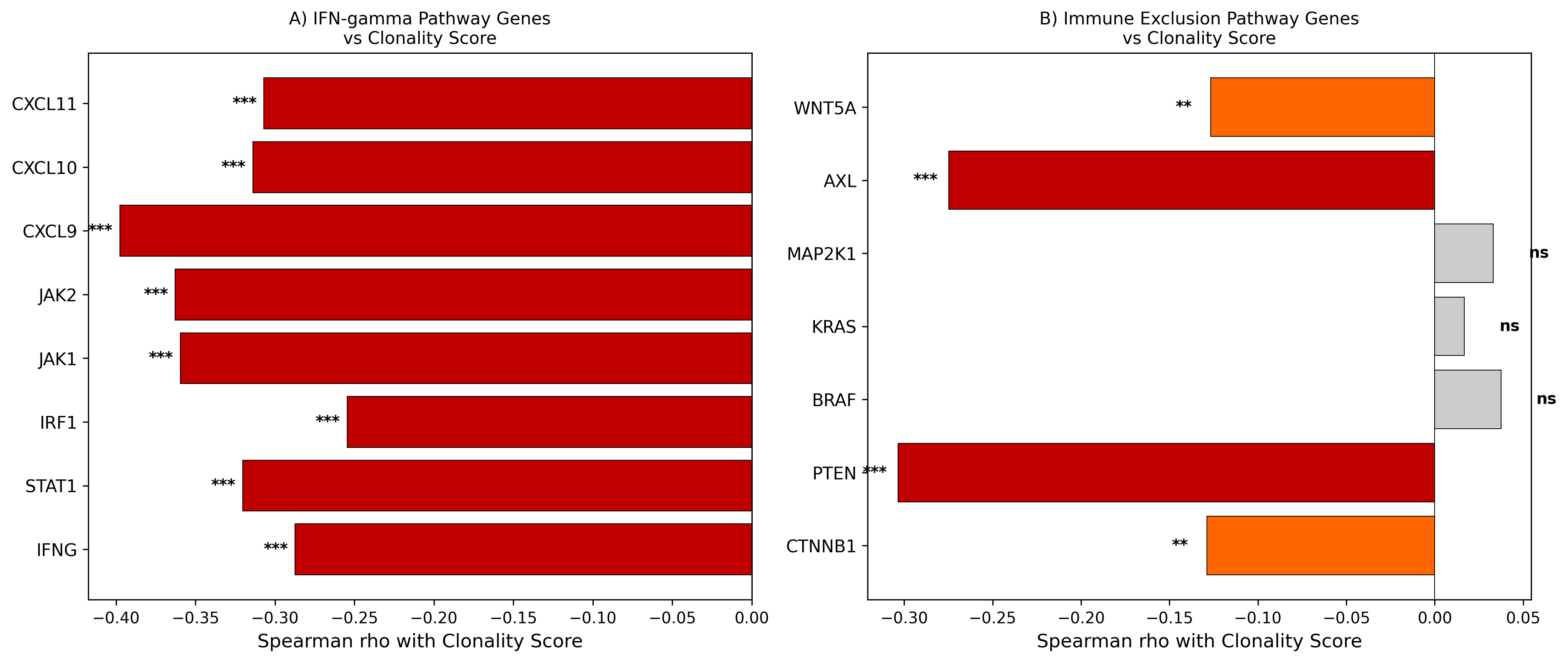

Supplement: Supplementary file 3 — Supplementary Material 3 [file 12967_2026_8371_MOESM3_ESM.docx]
