# Supplementary material for: Inverse relationship between neoantigen clonality and T-cell activity reveals distinct immune phenotypes in HNSCC
Source: J Transl Med. 2026 Jun 3;24:731. doi: 10.1186/s12967-026-08371-z (PMC13235206; doi:10.1186/s12967-026-08371-z)

**Supplementary Figure S4 | Kaplan–Meier survival analysis by immune phenotype and HPV-stratified clonality.**

(A) Kaplan–Meier curves for overall survival stratified by the four immune phenotypes defined by median dichotomization of Clonality Score and TIDE dysfunction. Hot/Low Clonality (green) and Hot/High Clonality (orange) tumours show more favourable survival compared to cold phenotypes. Log-rank test across all four groups is shown. (B) Kaplan–Meier curves for overall survival within immunologically ‘hot’ tumours (TIDE dysfunction ≥ median), further stratified by HPV status and median Clonality Score. This analysis demonstrates that the survival benefit of high clonality in hot tumours is consistent across HPV strata, and that HPV status does not confound the clonality-survival relationship within immune-infiltrated tumours. Sample sizes for each subgroup are indicated in the legend.


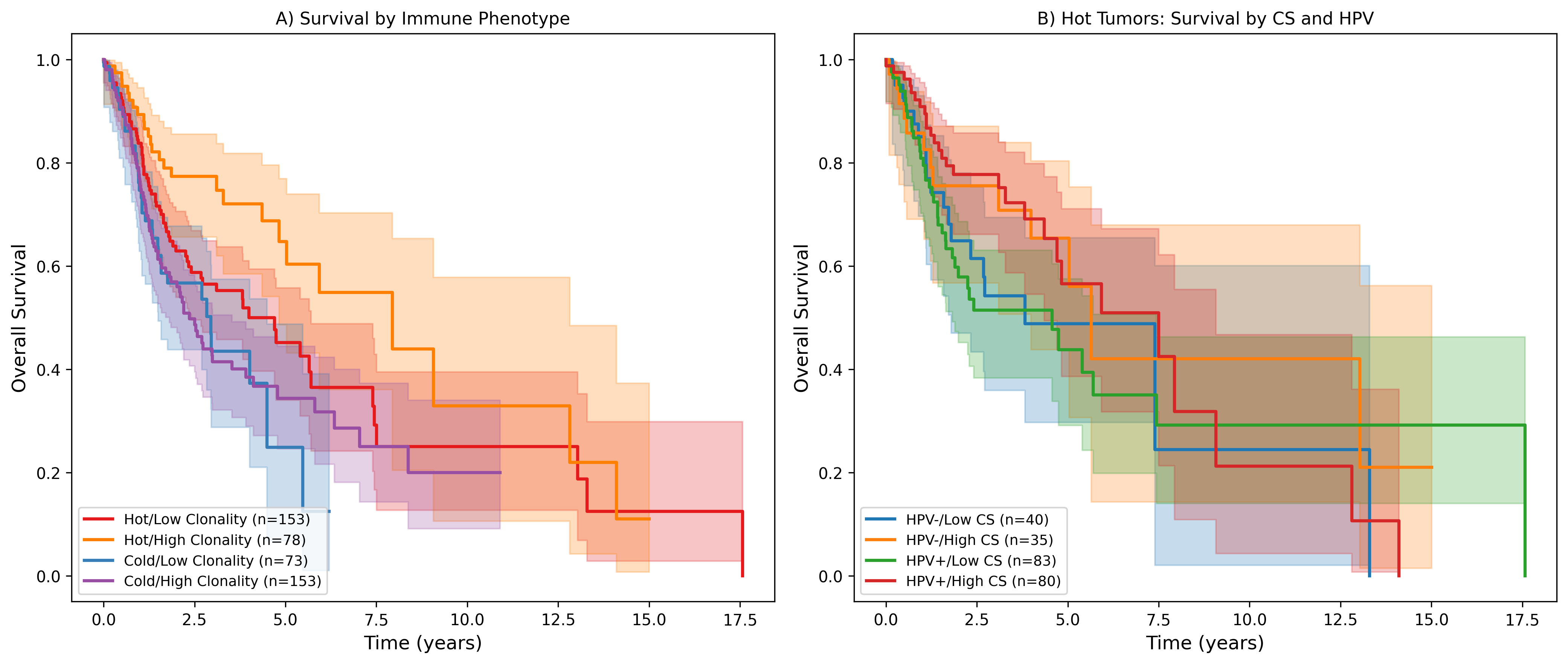

Supplement: Supplementary file 4 — Supplementary Material 4 [file 12967_2026_8371_MOESM4_ESM.docx]
