# Supplementary material for: Inverse relationship between neoantigen clonality and T-cell activity reveals distinct immune phenotypes in HNSCC
Source: J Transl Med. 2026 Jun 3;24:731. doi: 10.1186/s12967-026-08371-z (PMC13235206; doi:10.1186/s12967-026-08371-z)

**Supplementary Figure S5 | Alternative immune score correlations with the Clonality Score.**

Scatter plots showing correlations between the Clonality Score (x-axis) and nine alternative immune-context metrics derived from bulk RNA-seq and gene-expression deconvolution. Spearman correlation coefficients (rho) and P-values are reported in each panel. Metrics include individual T-cell marker genes (CD3D, GZMB, GNLY, NKG7), cytolytic activity (CYT), an ESTIMATE-style composite immune score, the IFN-γ Composite Score, the Wolf LIexpression score, and the Immunologic Constant of Rejection (ICR) score. The Clonality Score shows significant negative correlations with all nine alternative immune metrics, demonstrating that the inverse clonality–immune relationship is robust across diverse computational frameworks and not specific to a single bulk-RNA-seq-derived metric.


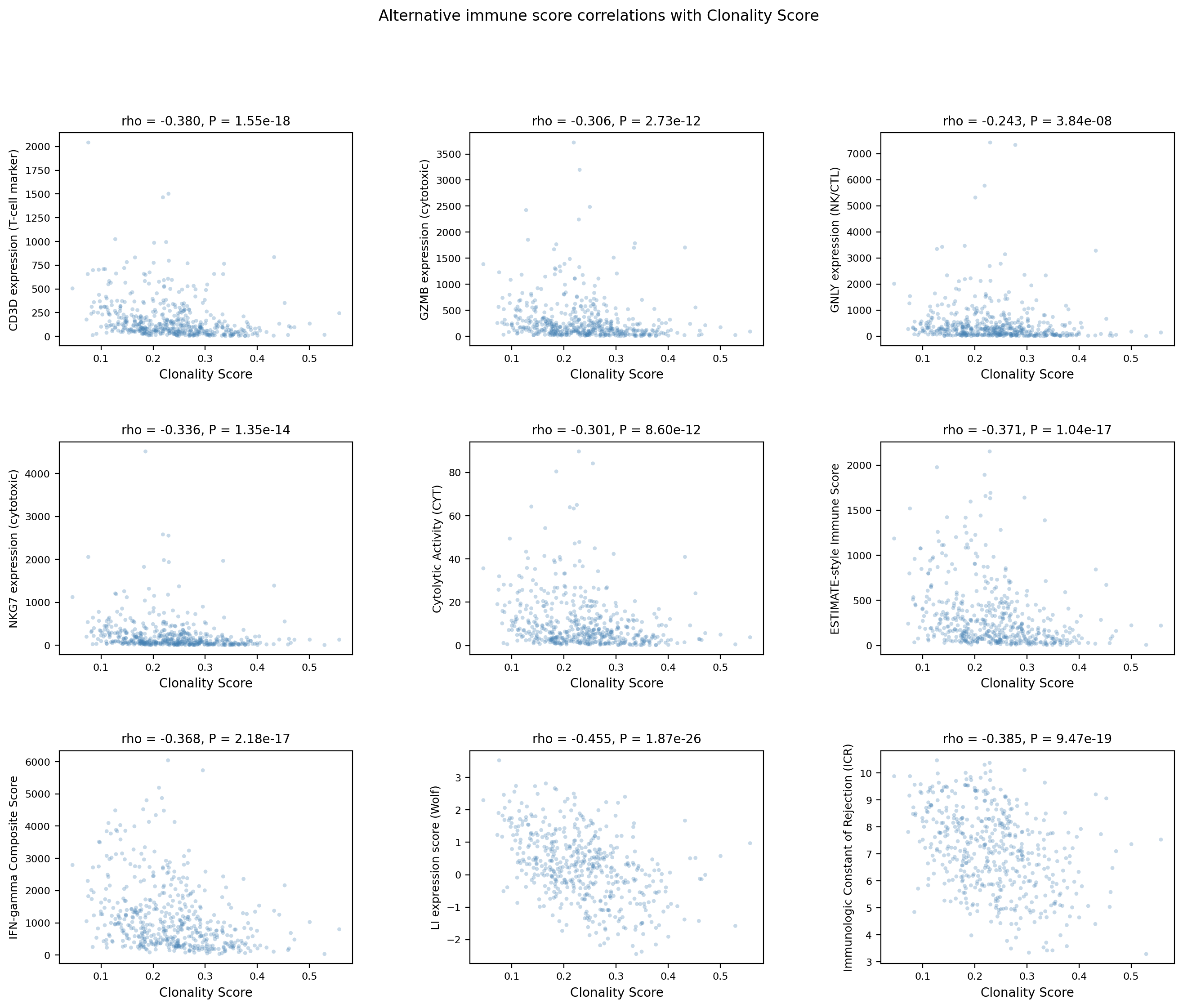

Supplement: Supplementary file 5 — Supplementary Material 5 [file 12967_2026_8371_MOESM5_ESM.docx]
