# Supplementary material for: Inverse relationship between neoantigen clonality and T-cell activity reveals distinct immune phenotypes in HNSCC
Source: J Transl Med. 2026 Jun 3;24:731. doi: 10.1186/s12967-026-08371-z (PMC13235206; doi:10.1186/s12967-026-08371-z)

**Supplementary Figure S6 | Pairwise log-rank tests and categorical Cox model for the four immune phenotypes.**

(A) Heatmap of FDR-adjusted log-rank P-values for all six pairwise comparisons between the four immune phenotypes (Hot/Low Clonality, Hot/High Clonality, Cold/Low Clonality, Cold/High Clonality). Cell shading reflects -log10(P_FDR); annotations show P_FDR and significance labels (***P < 0.001, **P < 0.01, *P < 0.05, ns = not significant after FDR correction). Three comparisons reach FDR significance, all involving the Hot/High Clonality group. (B) Forest plot of hazard ratios from a multivariable Cox proportional hazards model treating the four-phenotype variable as a single categorical predictor (reference: Hot/Low Clonality), adjusted for age, advanced stage, and HPV status (n = 405). Hot/High Clonality tumours show significantly improved survival relative to Hot/Low Clonality (HR = 0.54, P = 0.026), whereas the cold-phenotype groups do not differ significantly from the reference. Error bars indicate 95% confidence intervals; dashed line indicates HR = 1 (no effect).


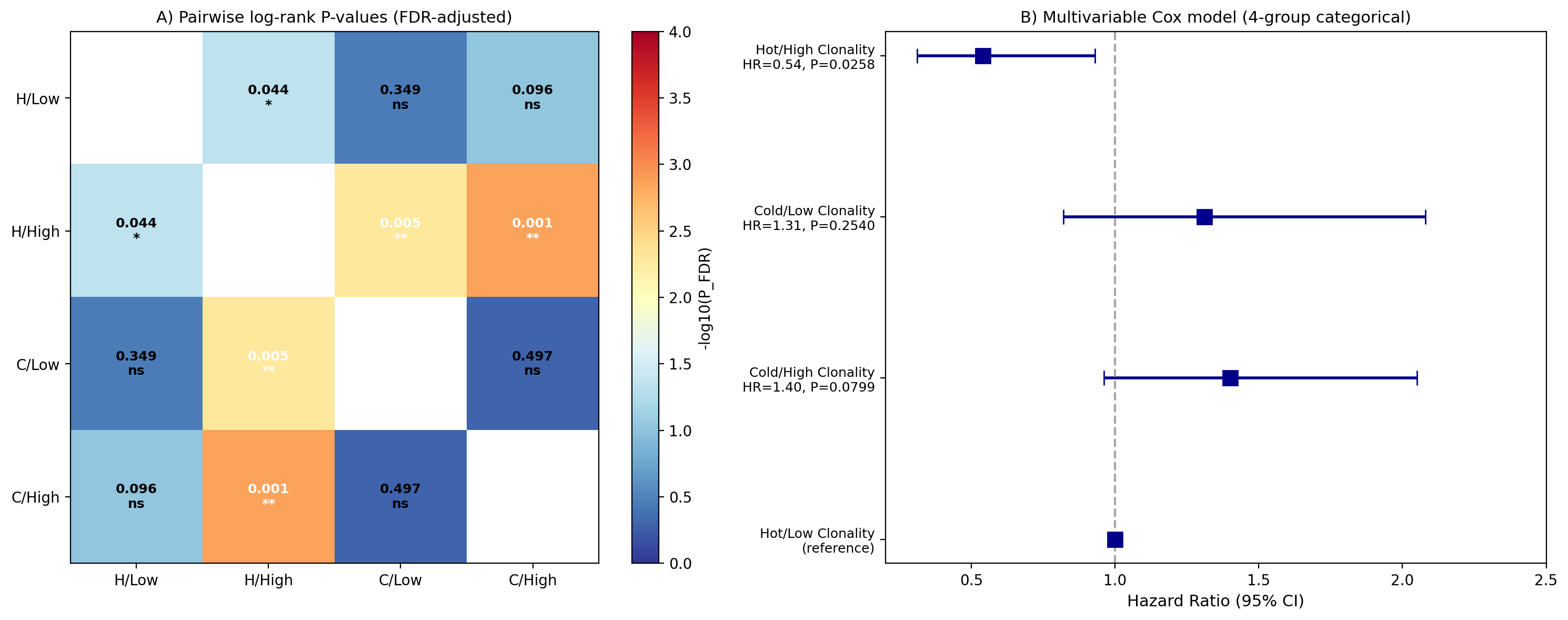

Supplement: Supplementary file 6 — Supplementary Material 6 [file 12967_2026_8371_MOESM6_ESM.docx]
