# Supplementary material for: Inverse relationship between neoantigen clonality and T-cell activity reveals distinct immune phenotypes in HNSCC
Source: J Transl Med. 2026 Jun 3;24:731. doi: 10.1186/s12967-026-08371-z (PMC13235206; doi:10.1186/s12967-026-08371-z)
Supplement: Supplementary file 7 — Supplementary Material 7 [file 12967_2026_8371_MOESM7_ESM.docx]

**Supplementary Table S1 | Correlations between neo‑antigen metrics and immune markers.**

Spearman correlation coefficients (ρ), two‑sided P values, and Benjamini–Hochberg FDR‑adjusted P values for associations between neo‑antigen metrics (TMB, neo_n_500, neo_frac_strong, binder‑weighted VAF, ΔIC50) and immune markers (CYT, CD8 fraction, PD‑L1, TIDE dysfunction, TIDE exclusion). Significant pairs after FDR correction include: binder‑weighted VAF vs TIDE dysfunction (ρ = −0.304; FDR = 1.01 × 10⁻¹⁰), binder‑weighted VAF vs PD‑L1 (ρ = −0.125; FDR = 0.043), and TMB vs TIDE dysfunction (ρ = −0.136; FDR = 2.96×10⁻²). Full results are provided in the table.

| Metric | Immune marker | Spearman ρ | P value | FDR-adjusted P |
| --- | --- | --- | --- | --- |
| TMB | CYT | −0.010 | 8.20×10⁻¹ | 9.32×10⁻¹ |
| TMB | CD8 fraction | 0.047 | 2.95×10⁻¹ | 6.71×10⁻¹ |
| TMB | PD-L1 (CD274) | −0.023 | 6.02×10⁻¹ | 8.36×10⁻¹ |
| TMB | **TIDE dysfunction** | **−0.136** | **2.37×10⁻³** | **2.96×10⁻²** |
| TMB | TIDE exclusion | −0.018 | 6.95×10⁻¹ | 8.97×10⁻¹ |
| neo_n_500 | CYT | 0.014 | 7.56×10⁻¹ | 9.00×10⁻¹ |
| neo_n_500 | CD8 fraction | 0.055 | 2.18×10⁻¹ | 6.71×10⁻¹ |
| neo_n_500 | PD-L1 (CD274) | −0.001 | 9.90×10⁻¹ | 9.91×10⁻¹ |
| neo_n_500 | TIDE dysfunction | −0.102 | 2.26×10⁻² | 1.13×10⁻¹ |
| neo_n_500 | TIDE exclusion | −0.033 | 4.63×10⁻¹ | 8.07×10⁻¹ |
| neo_frac_strong | CYT | −0.024 | 6.01×10⁻¹ | 8.36×10⁻¹ |
| neo_frac_strong | CD8 fraction | 0.030 | 5.09×10⁻¹ | 8.07×10⁻¹ |
| neo_frac_strong | PD-L1 (CD274) | −0.048 | 2.89×10⁻¹ | 6.71×10⁻¹ |
| neo_frac_strong | TIDE dysfunction | −0.069 | 1.22×10⁻¹ | 4.78×10⁻¹ |
| neo_frac_strong | TIDE exclusion | −0.007 | 8.77×10⁻¹ | 9.35×10⁻¹ |
| neo_binder_weighted_vaf_500 | CYT | −0.105 | 1.95×10⁻² | 1.13×10⁻¹ |
| neo_binder_weighted_vaf_500 | CD8 fraction | 0.016 | 7.18×10⁻¹ | 8.97×10⁻¹ |
| neo_binder_weighted_vaf_500 | **PD-L1 (CD274)** | **−0.125** | **5.18×10⁻³** | **4.31×10⁻²** |
| neo_binder_weighted_vaf_500 | **TIDE dysfunction** | **−0.304** | **4.03×10⁻¹²** | **1.01×10⁻¹⁰** |
| neo_binder_weighted_vaf_500 | TIDE exclusion | 0.067 | 1.34×10⁻¹ | 4.79×10⁻¹ |
| neo_mean_delta_ic50 | CYT | −0.040 | 3.70×10⁻¹ | 7.71×10⁻¹ |
| neo_mean_delta_ic50 | CD8 fraction | −0.029 | 5.16×10⁻¹ | 8.07×10⁻¹ |
| neo_mean_delta_ic50 | PD-L1 (CD274) | −0.050 | 2.61×10⁻¹ | 6.71×10⁻¹ |
| neo_mean_delta_ic50 | TIDE dysfunction | −0.006 | 8.98×10⁻¹ | 9.35×10⁻¹ |
| neo_mean_delta_ic50 | TIDE exclusion | 0.038 | 4.03×10⁻¹ | 7.74×10⁻¹ |
