# Supplementary material for: Inverse relationship between neoantigen clonality and T-cell activity reveals distinct immune phenotypes in HNSCC
Source: J Transl Med. 2026 Jun 3;24:731. doi: 10.1186/s12967-026-08371-z (PMC13235206; doi:10.1186/s12967-026-08371-z)
Supplement: Supplementary file 8 — Supplementary Material 8 [file 12967_2026_8371_MOESM8_ESM.docx]

**Supplementary Table S2 | Spearman correlations between immune‑cell fractions and neo‑antigen/mutational features.**

The supplementary table provides the full correlation matrix (ρ values) with P values and FDR‑adjusted P values, formatted in scientific notation. FDR‑significant associations include: memory‑activated CD4 T cells vs TMB (FDR = 2.88 × 10⁻²), memory‑activated CD4 T cells vs neo_n_500 (FDR = 3.29 × 10⁻²), Tregs vs TMB (FDR = 2.88 × 10⁻²) and Tregs vs binder‑weighted VAF (FDR = 8.92 × 10⁻³).

| Immune cell | Feature | Spearman ρ | P value | FDR-adjusted P |
| --- | --- | --- | --- | --- |
| T.cells.CD8 | TMB | 0.047 | 2.953e-01 | 6.930e-01 |
| T.cells.CD8 | neo_n_500 | 0.055 | 2.178e-01 | 6.534e-01 |
| T.cells.CD8 | neo_binder_weighted_vaf_500 | 0.016 | 7.179e-01 | 7.832e-01 |
| T.cells.CD8 | neo_mean_delta_ic50 | –0.029 | 5.164e-01 | 7.290e-01 |
| T.cells.CD4.memory.activated | **TMB** | **0.130** | **3.597e-03** | **2.878e-02** |
| T.cells.CD4.memory.activated | **neo_n_500** | **0.124** | **5.487e-03** | **3.292e-02** |
| T.cells.CD4.memory.activated | neo_binder_weighted_vaf_500 | 0.111 | 1.315e-02 | 6.311e-02 |
| T.cells.CD4.memory.activated | neo_mean_delta_ic50 | –0.034 | 4.473e-01 | 7.278e-01 |
| NK.cells.activated | TMB | –0.030 | 4.967e-01 | 7.290e-01 |
| NK.cells.activated | neo_n_500 | –0.045 | 3.176e-01 | 6.930e-01 |
| NK.cells.activated | neo_binder_weighted_vaf_500 | –0.008 | 8.556e-01 | 8.905e-01 |
| NK.cells.activated | neo_mean_delta_ic50 | 0.006 | 8.905e-01 | 8.905e-01 |
| Macrophages.M1 | TMB | –0.037 | 4.048e-01 | 7.278e-01 |
| Macrophages.M1 | neo_n_500 | –0.024 | 5.888e-01 | 7.465e-01 |
| Macrophages.M1 | neo_binder_weighted_vaf_500 | –0.090 | 4.460e-02 | 1.529e-01 |
| Macrophages.M1 | neo_mean_delta_ic50 | 0.049 | 2.772e-01 | 6.930e-01 |
| Macrophages.M2 | TMB | 0.024 | 5.910e-01 | 7.465e-01 |
| Macrophages.M2 | neo_n_500 | 0.039 | 3.830e-01 | 7.278e-01 |
| Macrophages.M2 | neo_binder_weighted_vaf_500 | 0.034 | 4.549e-01 | 7.278e-01 |
| Macrophages.M2 | neo_mean_delta_ic50 | 0.021 | 6.416e-01 | 7.700e-01 |
| T.cells.regulatory.Tregs. | **TMB** | **–0.131** | **3.462e-03** | **2.878e-02** |
| T.cells.regulatory.Tregs. | neo_n_500 | –0.099 | 2.653e-02 | 1.061e-01 |
| T.cells.regulatory.Tregs. | **neo_binder_weighted_vaf_500** | **–0.159** | **3.715e-04** | **8.915e-03** |
| T.cells.regulatory.Tregs. | neo_mean_delta_ic50 | –0.017 | 7.080e-01 | 7.832e-01 |
