# Supplementary material for: Inverse relationship between neoantigen clonality and T-cell activity reveals distinct immune phenotypes in HNSCC
Source: J Transl Med. 2026 Jun 3;24:731. doi: 10.1186/s12967-026-08371-z (PMC13235206; doi:10.1186/s12967-026-08371-z)
Supplement: Supplementary file 9 — Supplementary Material 9 [file 12967_2026_8371_MOESM9_ESM.docx]

**Supplementary Table S3 | Comparison of neo‑antigen and immune metrics between LOH‑positive and LOH‑negative tumours.**

The supplementary table presents medians and interquartile ranges (IQR) for neo‑epitope burden, binder‑weighted clonal neo‑antigen load, tumour mutational burden (TMB), cytolytic activity (CYT) and TIDE scores in tumours with HLA loss of heterozygosity (LOH+) versus those without LOH (LOH–). Mann–Whitney U tests were used to compare groups. The only significant difference was observed for the TIDE dysfunction score, which was lower in LOH‑positive tumours (median –0.071 vs 0.067; P = 0.048), suggesting reduced T-cell infiltration despite similar neo‑epitope burden and binder‑weighted VAF across LOH strata.

| Variable | LOH+ median | LOH+ IQR | n  LOH+ | LOH‑  median | LOH‑  IQR | n  LOH‑ | Mann–Whitney *P* |
| --- | --- | --- | --- | --- | --- | --- | --- |
| neo_n_500 (binders) | 1 124.0 | 1 109.0 | 189 | 1 328.5 | 1 252.8 | 316 | 0.1450 |
| neo_binder_weighted_vaf_500 | 256.09 | 273.43 | 189 | 287.43 | 349.04 | 316 | 0.5994 |
| TMB (mut/Mb) | 2.665 | 1.931 | 190 | 2.844 | 2.695 | 317 | 0.4641 |
| CYT | 6.31 | 10.33 | 186 | 6.85 | 12.33 | 329 | 0.4422 |
| TIDE_dysfunction | **–0.071** | **1.012** | **187** | **0.067** | **1.031** | **333** | **0.0484** |
| TIDE_exclusion | –0.020 | 1.618 | 187 | 0.101 | 1.540 | 333 | 0.9099 |
