# Supplementary material for: Inverse relationship between neoantigen clonality and T-cell activity reveals distinct immune phenotypes in HNSCC
Source: J Transl Med. 2026 Jun 3;24:731. doi: 10.1186/s12967-026-08371-z (PMC13235206; doi:10.1186/s12967-026-08371-z)
Supplement: Supplementary file 10 — Supplementary Material 10 [file 12967_2026_8371_MOESM10_ESM.docx]

**Supplementary Table S4 | HLA supertype coverage statistics and immune correlations.**

This supplementary table provides comprehensive statistics for each HLA supertype, including: the number of patients (n), total public peptides, mean and median peptides per patient, coverage fraction using the top 10 and top 50 patients, Spearman correlations (ρ) between public peptide counts and PD-L1 expression or cytolytic activity (CYT), and corresponding P values. HLA-A/B supertypes are distinguished from HLA-C supertypes. Supertypes with fewer than 5 patients were excluded from correlation analyses due to insufficient power.

| Supertype | HLA Class | n | Total Peptides | Mean/Patient | Median/Patient | Coverage (Top 10) | Coverage (Top 50) | ρ PD-L1 | P PD-L1 | ρ CYT | P CYT |
| --- | --- | --- | --- | --- | --- | --- | --- | --- | --- | --- | --- |
| C07 | HLA-C | 111 | 1123 | 10.1 | 5.0 | 0.136 | 0.229 | 0.038 | 0.696 | −0.012 | 0.905 |
| C03 | HLA-C | 65 | 765 | 11.8 | 2.0 | 0.136 | 0.178 | 0.045 | 0.721 | −0.077 | 0.540 |
| C12 | HLA-C | 50 | 575 | 11.5 | 6.0 | 0.085 | 0.134 | 0.031 | 0.834 | −0.100 | 0.495 |
| Other | HLA-C | 68 | 485 | 7.1 | 4.5 | 0.059 | 0.111 | 0.121 | 0.340 | 0.134 | 0.290 |
| C16 | HLA-C | 43 | 316 | 7.3 | 3.0 | 0.051 | 0.073 | 0.042 | 0.794 | 0.297 | 0.059 |
| C01 | HLA-C | 16 | 202 | 12.6 | 2.5 | 0.047 | 0.047 | −0.033 | 0.906 | 0.111 | 0.694 |
| B44 | HLA-A/B | 19 | 131 | 6.9 | 4.0 | 0.028 | 0.030 | 0.018 | 0.943 | 0.020 | 0.934 |
| C05 | HLA-C | 19 | 129 | 6.8 | 5.0 | 0.029 | 0.030 | −0.239 | 0.325 | −0.106 | 0.665 |
| C15 | HLA-C | 15 | 128 | 8.5 | 8.0 | 0.028 | 0.030 | **0.810** | **2.5×10⁻⁴** | **0.799** | **3.5×10⁻⁴** |
| A02 | HLA-A/B | 25 | 100 | 4.0 | 2.0 | 0.020 | 0.023 | 0.128 | 0.543 | 0.154 | 0.463 |
| B58 | HLA-A/B | 16 | 85 | 5.3 | 2.0 | 0.020 | 0.020 | — | — | — | — |
| C06 | HLA-C | 14 | 57 | 4.1 | 3.5 | 0.013 | 0.013 | — | — | — | — |
| B27 | HLA-A/B | 6 | 57 | 9.5 | 8.5 | 0.013 | 0.013 | — | — | — | — |
| A03 | HLA-A/B | 13 | 48 | 3.7 | 3.0 | 0.011 | 0.011 | — | — | — | — |
| B07 | HLA-A/B | 7 | 37 | 5.3 | 5.0 | 0.009 | 0.009 | — | — | — | — |
| B08 | HLA-A/B | 7 | 31 | 4.4 | 3.0 | 0.007 | 0.007 | — | — | — | — |
