# Supplementary material for: Inverse relationship between neoantigen clonality and T-cell activity reveals distinct immune phenotypes in HNSCC
Source: J Transl Med. 2026 Jun 3;24:731. doi: 10.1186/s12967-026-08371-z (PMC13235206; doi:10.1186/s12967-026-08371-z)
Supplement: Supplementary file 11 — Supplementary Material 11 [file 12967_2026_8371_MOESM11_ESM.docx]

**Supplementary Table S5 | Sensitivity Analysis Across Alternative Clonality Metrics.**

Spearman correlation coefficients (ρ) between five clonality metrics and eight immune variables in the HNSCC cohort. All five metrics—each capturing variant allele frequency weighting—reproduce significant negative correlations with all immune variables, demonstrating that the observed clonality–immune relationship is robust to metric definition. Significance: ***P < 0.001, **P < 0.01, *P < 0.05.

| **Immune Variable** | **Clonality Score (primary)** | **Mean VAF per binder** | **Binder-weighted VAF (raw)** | **BwVAF / (total neoAg + 1)** | **Log(Clonality Score + 1)** |
| --- | --- | --- | --- | --- | --- |
| **Exhaustion Score** | -0.412*** | -0.411*** | -0.203*** | -0.296*** | -0.412*** |
| **Core Exhaustion Score** | -0.373*** | -0.372*** | -0.185*** | -0.262*** | -0.373*** |
| **Pan-Immune Score** | -0.500*** | -0.500*** | -0.263*** | -0.382*** | -0.500*** |
| **TIDE Dysfunction** | -0.533*** | -0.533*** | -0.304*** | -0.446*** | -0.533*** |
| **Cytolytic Activity (CYT)** | -0.301*** | -0.300*** | -0.105* | -0.225*** | -0.301*** |
| **Antigen Presentation Score** | -0.312*** | -0.312*** | -0.161*** | -0.227*** | -0.312*** |
| **MHC Class I Score** | -0.240*** | -0.240*** | -0.127** | -0.199*** | -0.240*** |
| **Immunosuppressive Gene Score** | -0.409*** | -0.408*** | -0.274*** | -0.336*** | -0.409*** |
