# Supplementary material for: Inverse relationship between neoantigen clonality and T-cell activity reveals distinct immune phenotypes in HNSCC
Source: J Transl Med. 2026 Jun 3;24:731. doi: 10.1186/s12967-026-08371-z (PMC13235206; doi:10.1186/s12967-026-08371-z)
Supplement: Supplementary file 12 — Supplementary Material 12 [file 12967_2026_8371_MOESM12_ESM.docx]

**Supplementary Table S6 | Partial Correlations Controlling for Immune Abundance.**

Spearman partial correlations between Clonality Score and immune variables, controlling separately for Pan-Immune Score, CD8 T-cell fraction (CIBERSORT), and total lymphocyte fraction (n = 497). Controlling for Pan-Immune Score substantially attenuates or reverses most correlations (indicating infiltration-mediated confounding), whereas controlling for CD8 fraction alone has minimal effect. TIDE dysfunction retains a residual partial ρ = −0.233 after Pan-Immune adjustment, suggesting a clonality-specific effect beyond immune abundance.

| **Variable** | **Unadjusted ρ** | **Adj. Pan-Immune ρ** | **Adj. Pan-Immune p** | **Adj. CD8 ρ** | **Adj. Lymphocyte ρ** | **Interpretation** |
| --- | --- | --- | --- | --- | --- | --- |
| **Exhaustion Score** | -0.412 | 0.089 | 0.047 | -0.429 | -0.441 | Infiltration-mediated |
| **Core Exhaustion Score** | -0.373 | 0.183 | 4.3e-5 | -0.391 | -0.396 | Infiltration-mediated |
| **TIDE dysfunction** | -0.533 | -0.233 | 1.5e-7 | -0.543 | -0.542 | Infiltration-mediated |
| **CYT** | -0.301 | 0.201 | 6.9e-6 | -0.320 | -0.319 | Residual signal |
| **Antigen Presentation Score** | -0.312 | 0.093 | 0.038 | -0.313 | -0.313 | Infiltration-mediated |
| **MHC Class I Score** | -0.240 | -0.003 | 0.940 | -0.228 | -0.225 | Infiltration-mediated |
| **Immunosuppressive Gene Score** | -0.409 | -0.141 | 0.002 | -0.403 | -0.403 | Infiltration-mediated |

Interpretation: 'Infiltration-mediated' = partial ρ attenuated by >50% vs unadjusted, indicating the association is largely driven by immune infiltration differences. 'Residual signal' = substantial correlation persists after adjustment.
