# Supplementary material for: Inverse relationship between neoantigen clonality and T-cell activity reveals distinct immune phenotypes in HNSCC
Source: J Transl Med. 2026 Jun 3;24:731. doi: 10.1186/s12967-026-08371-z (PMC13235206; doi:10.1186/s12967-026-08371-z)
Supplement: Supplementary file 13 — Supplementary Material 13 [file 12967_2026_8371_MOESM13_ESM.docx]

**Supplementary Table S7 | Stratified Correlations Within Hot and Cold Tumours.**

Spearman correlations between Clonality Score and immune variables stratified by immune status (Hot: Pan-Immune Score ≥ median, n = 249; Cold: Pan-Immune Score < median, n = 248). Within immune-hot tumours, TIDE dysfunction retains ρ = −0.417 (P = 7.2 × 10⁻¹²) and 11 of 17 individual exhaustion genes remain significantly negatively correlated with clonality (P < 0.05), demonstrating that the association is not purely a hot/cold confounder.

| **Variable** | **n** | **All (ρ, p)** | **Hot (ρ, p)** | **Cold (ρ, p)** |
| --- | --- | --- | --- | --- |
| **Exhaustion Score** | 497 | -0.412, 9.8e-22 | -0.223, 4.0e-4 | -0.149, 0.019 |
| **Core Exhaustion Score** | 497 | -0.373, 7.5e-18 | -0.132, 0.038 | -0.108, 0.090 |
| **TIDE dysfunction** | 497 | -0.533, 9.1e-38 | -0.417, 7.2e-12 | -0.381, 5.3e-10 |
| **CYT** | 495 | -0.301, 8.6e-12 | -0.071, 0.265 | -0.032, 0.616 |
| **Antigen Presentation Score** | 497 | -0.312, 1.0e-12 | -0.061, 0.339 | -0.130, 0.041 |
| **MHC Class I Score** | 497 | -0.240, 6.1e-8 | -0.086, 0.175 | -0.102, 0.108 |
| **Immunosuppressive Gene Score** | 497 | -0.409, 1.9e-21 | -0.242, 1.1e-4 | -0.263, 2.7e-5 |
| **Pan-Immune Score** | 497 | -0.500, 8.5e-33 | -0.377, 7.8e-10 | -0.270, 1.7e-5 |

TIDE dysfunction retains strong significant correlation within both hot (ρ = −0.417) and cold (ρ = −0.382) tumours. Most other metrics show attenuated but directionally consistent correlations within strata.
