# Supplementary material for: Inverse relationship between neoantigen clonality and T-cell activity reveals distinct immune phenotypes in HNSCC
Source: J Transl Med. 2026 Jun 3;24:731. doi: 10.1186/s12967-026-08371-z (PMC13235206; doi:10.1186/s12967-026-08371-z)
Supplement: Supplementary file 14 — Supplementary Material 14 [file 12967_2026_8371_MOESM14_ESM.docx]

**Supplementary Table S8 | HPV-stratified correlation analysis between clonality and immune variables.**

This table presents Spearman correlations between neoantigen clonality and immune variables stratified by HPV status. The inverse clonality-immune relationships are preserved in both HPV-positive and HPV-negative subgroups, with comparable correlation strengths across strata. All associations remain highly significant (P < 0.001) regardless of HPV status, with the exception of Antigen Presentation Score in HPV-positive tumours (P = 0.18). These findings demonstrate that the clonality-immune relationship represents a fundamental tumour biology principle independent of HPV-driven carcinogenesis.

| HPV Status | Variable 1 | Variable 2 | N | Spearman ρ | P-value | Sig |
| --- | --- | --- | --- | --- | --- | --- |
| HPV+ | Clonality Score | Exhaustion Score | 65 | -0.449 | 1.73e-04 | *** |
| HPV+ | Clonality Score | TIDE dysfunction | 65 | -0.417 | 5.45e-04 | *** |
| HPV+ | Clonality Score | Pan-Immune Score | 65 | -0.570 | 7.20e-07 | *** |
| HPV+ | Clonality Score | CYT | 64 | -0.422 | 5.12e-04 | *** |
| HPV+ | Clonality Score | Antigen Presentation Score | 65 | -0.170 | 0.18 | ns |
| HPV- | Clonality Score | Exhaustion Score | 432 | -0.457 | 1.02e-23 | *** |
| HPV- | Clonality Score | TIDE dysfunction | 433 | -0.558 | 8.56e-37 | *** |
| HPV- | Clonality Score | Pan-Immune Score | 432 | -0.535 | 2.49e-33 | *** |
| HPV- | Clonality Score | CYT | 431 | -0.312 | 3.27e-11 | *** |
| HPV- | Clonality Score | Antigen Presentation Score | 432 | -0.346 | 1.40e-13 | *** |
