# Supplementary material for: Inverse relationship between neoantigen clonality and T-cell activity reveals distinct immune phenotypes in HNSCC
Source: J Transl Med. 2026 Jun 3;24:731. doi: 10.1186/s12967-026-08371-z (PMC13235206; doi:10.1186/s12967-026-08371-z)
Supplement: Supplementary file 15 — Supplementary Material 15 [file 12967_2026_8371_MOESM15_ESM.docx]

**Supplementary Table S9 | Validation of Clonality Score against ABSOLUTE Subclonal Genome Fraction and purity-adjusted partial correlations.**

Panel A: Spearman correlation coefficients (ρ) between immune variables and two independent clonality metrics: the Clonality Score (CS) and the ABSOLUTE-estimated Subclonal Genome Fraction (SGF). The CS correlates negatively with SGF (ρ = −0.262, P = 3.95 × 10⁻⁹, n = 488), validating it as a clonality proxy. SGF independently reproduces key immune associations, confirming the inverse clonality-immune relationship reflects genuine biology. Panel B: Partial Spearman correlations between CS and immune variables after controlling for ABSOLUTE tumour purity. Core associations are preserved after purity adjustment, indicating that the clonality-immune relationship is not solely driven by tumour cellularity confounding.

**Panel A: CS vs SGF validation**

| **Immune Variable** | **CS rho** | **CS p-value** | **SGF rho** | **SGF p-value** | **Direction Concordant** | **n** |
| --- | --- | --- | --- | --- | --- | --- |
| Exhaustion Score | -0.471 | 6.83e-28 | 0.009 | 0.837 | Yes | 481 |
| TIDE Dysfunction | -0.534 | 7.79e-37 | 0.024 | 0.603 | Yes | 481 |
| Cytolytic Activity | -0.3 | 2.07e-11 | -0.094 | 0.0396 | Yes | 478 |
| CD8+ T cells (CIBERSORT) | -0.077 | 0.0905 | -0.165 | 0.000268 | Yes | 481 |
| CD4+ Memory Activated T cells | 0.01 | 0.822 | -0.114 | 0.0122 | Opposite* | 481 |
| NK cells Activated | 0.033 | 0.473 | -0.09 | 0.0489 | Opposite* | 481 |
| M1 Macrophages | -0.203 | 6.97e-06 | -0.093 | 0.0405 | Yes | 481 |
| PD-L1 Expression | -0.328 | 1.5e-13 | -0.033 | 0.475 | Yes | 481 |
| APM Score | -0.367 | 8.6e-17 | -0.081 | 0.0766 | Yes | 481 |
| IFN-gamma Score | -0.368 | 6.58e-17 | -0.113 | 0.0132 | Yes | 481 |

**Panel B: Partial correlations controlling for ABSOLUTE purity**

| **Immune Variable** | **Unadjusted rho** | **Purity-adjusted rho** | **Purity-adjusted p** | **n** |
| --- | --- | --- | --- | --- |
| Exhaustion Score | -0.471 | -0.273 | 1.17e-09 | 481 |
| TIDE Dysfunction | -0.534 | -0.256 | 1.25e-08 | 481 |
| Cytolytic Activity | -0.3 | -0.178 | 9.01e-05 | 478 |
| CD8+ T cells (CIBERSORT) | -0.077 | -0.061 | 0.185 | 481 |
| CD4+ Memory Activated T cells | 0.01 | 0.03 | 0.513 | 481 |
| NK cells Activated | 0.033 | -0.031 | 0.504 | 481 |
| M1 Macrophages | -0.203 | -0.112 | 0.0141 | 481 |
| PD-L1 Expression | -0.328 | -0.15 | 0.000961 | 481 |
| APM Score | -0.367 | -0.115 | 0.0116 | 481 |
| IFN-gamma Score | -0.368 | -0.192 | 2.15e-05 | 481 |
