# Supplementary material for: Inverse relationship between neoantigen clonality and T-cell activity reveals distinct immune phenotypes in HNSCC
Source: J Transl Med. 2026 Jun 3;24:731. doi: 10.1186/s12967-026-08371-z (PMC13235206; doi:10.1186/s12967-026-08371-z)
Supplement: Supplementary file 16 — Supplementary Material 16 [file 12967_2026_8371_MOESM16_ESM.docx]

**Supplementary Table S10 | IFN-γ signalling and immune exclusion pathway correlations with Clonality Score.**

Panel A: Spearman correlations between individual IFN-γ pathway genes and the Clonality Score, with purity-adjusted partial correlations. All ten IFN-γ pathway genes show significant negative correlations with clonality, suggesting attenuated IFN-γ signalling in high-clonality tumours. The composite IFN-γ Score and the pre-computed IFNG signature score from the TCGA PanImmune resource are also shown. Panel B: Correlations between immune exclusion pathway genes and both the Clonality Score and TIDE dysfunction. PTEN and AXL show significant negative correlations with clonality and positive correlations with TIDE dysfunction, consistent with roles in immune exclusion. MAPK pathway genes show no direct correlation with clonality but differ across immune phenotypes.

**Panel A: IFN-γ pathway**

| **Gene/Score** | **Spearman rho** | **p-value** | **Purity-adjusted rho** | **Purity-adjusted p** | **n** |
| --- | --- | --- | --- | --- | --- |
| IFNG | -0.287 | 6.4e-11 | -0.157 | 0.000535 | 498 |
| STAT1 | -0.32 | 2.45e-13 | -0.129 | 0.00448 | 498 |
| IRF1 | -0.255 | 8.26e-09 | -0.148 | 0.00116 | 498 |
| JAK1 | -0.36 | 1.22e-16 | -0.165 | 0.000284 | 498 |
| JAK2 | -0.363 | 6.21e-17 | -0.301 | 1.45e-11 | 498 |
| CXCL9 | -0.398 | 2.66e-20 | -0.205 | 5.64e-06 | 498 |
| CXCL10 | -0.314 | 7.53e-13 | -0.166 | 0.000246 | 498 |
| CXCL11 | -0.307 | 2.52e-12 | -0.116 | 0.0107 | 498 |
| IFNGR1 | -0.237 | 8.71e-08 | -0.194 | 1.85e-05 | 498 |
| IFNGR2 | -0.106 | 0.0183 | 0.01 | 0.832 | 498 |
| IFNg_Score | -0.368 | 2.18e-17 | -0.192 | 2.15e-05 | 498 |
| sig_IFNG_score_21050467 | -0.336 | 2.15e-14 | -0.07 | 0.126 | 491 |

**Panel B: Immune exclusion pathways**

| **Gene** | **Pathway** | **CS rho** | **CS p-value** | **TIDE_dys rho** | **TIDE_dys p-value** | **n** |
| --- | --- | --- | --- | --- | --- | --- |
| CTNNB1 | WNT/beta-catenin | -0.129 | 0.004 | 0.034 | 0.441 | 498 |
| PTEN | PTEN/PI3K | -0.304 | 4.48e-12 | 0.244 | 1.79e-08 | 498 |
| BRAF | MAPK | 0.037 | 0.405 | -0.11 | 0.0121 | 498 |
| KRAS | MAPK | 0.017 | 0.711 | -0.212 | 1.07e-06 | 498 |
| MAP2K1 | MAPK | 0.033 | 0.462 | -0.184 | 2.5e-05 | 498 |
| AXL | EMT/exclusion | -0.275 | 4.42e-10 | 0.426 | 2.5399999999999998e-24 | 498 |
| WNT5A | WNT/beta-catenin | -0.127 | 0.00462 | 0.009 | 0.837 | 498 |
