# Supplementary material for: Inverse relationship between neoantigen clonality and T-cell activity reveals distinct immune phenotypes in HNSCC
Source: J Transl Med. 2026 Jun 3;24:731. doi: 10.1186/s12967-026-08371-z (PMC13235206; doi:10.1186/s12967-026-08371-z)
Supplement: Supplementary file 17 — Supplementary Material 17 [file 12967_2026_8371_MOESM17_ESM.docx]

**Supplementary Table S11 | Multivariable Cox proportional hazards models with and without HPV adjustment.**

Hazard ratios (HR) with 95% confidence intervals from Cox regression models assessing the prognostic value of neoantigen clonality with and without HPV status as covariate. The original models (without HPV) demonstrate that Clonality Score independently predicts survival and that the prognostic effect depends on immune context (significant CS × TIDE interaction). HPV-adjusted models confirm that all associations are preserved after controlling for HPV status, which is not independently prognostic in any model. All continuous variables were z-standardised; hazard ratios represent the change in risk per one standard deviation increase. Hot tumours: TIDE dysfunction ≥ median; cold tumours: TIDE dysfunction < median. Stratified models adjusted for age at diagnosis and clinical stage. *P < 0.05, **P < 0.01.

| **Variable** | **HR (95% CI)** | **P-value** |
| --- | --- | --- |
| **Main Effects Model (n = 386, events = 153)** |  |  |
| Neoantigen count (neo_n_500) | 1.04 (0.89–1.20) | 0.64 |
| Clonality Score | 0.79 (0.64–0.98) | 0.030* |
| TIDE dysfunction | 0.72 (0.58–0.88) | 0.002** |
| TIDE exclusion | 1.23 (1.02–1.49) | 0.030* |
| PD-L1 expression (CD274) | 1.01 (0.87–1.17) | 0.93 |
| Age at diagnosis | 1.30 (1.09–1.55) | 0.003** |
| Advanced stage (III–IV) | 1.32 (0.89–1.94) | 0.16 |
| **HPV-Adjusted Main Effects Model (n = 386, events = 153)** |  |  |
| Neoantigen count (neo_n_500) | 1.04 (0.89–1.21) | 0.61 |
| Clonality Score | 0.79 (0.65–0.98) | 0.030* |
| TIDE dysfunction | 0.71 (0.57–0.88) | 0.002** |
| HPV positive | 1.12 (0.79–1.59) | 0.51 |
| **Interaction Model** |  |  |
| CS × TIDE dysfunction | 0.85 (0.72–0.99) | 0.034* |
| **HPV-Adjusted Interaction Model** |  |  |
| CS × TIDE dysfunction | 0.84 (0.71–0.98) | 0.030* |
| HPV positive | 1.15 (0.81–1.62) | 0.43 |
| **Stratified by Immune Status** |  |  |
| Hot tumours (n = 193): CS | 0.72 (0.53–0.99) | 0.043* |
| Cold tumours (n = 193): CS | 1.00 (0.79–1.27) | 0.98 |
| **HPV-Adjusted Stratified by Immune Status** |  |  |
| Hot tumours (n = 198): CS | 0.71 (0.52–0.97) | 0.029* |
| Hot tumours: HPV positive | 1.16 (0.68–1.98) | 0.59 |
| Cold tumours (n = 188): CS | 0.99 (0.78–1.26) | 0.96 |
| Cold tumours: HPV positive | 1.12 (0.71–1.76) | 0.62 |
