# Supplementary material for: Inverse relationship between neoantigen clonality and T-cell activity reveals distinct immune phenotypes in HNSCC
Source: J Transl Med. 2026 Jun 3;24:731. doi: 10.1186/s12967-026-08371-z (PMC13235206; doi:10.1186/s12967-026-08371-z)
Supplement: Supplementary file 18 — Supplementary Material 18 [file 12967_2026_8371_MOESM18_ESM.docx]

**Supplementary Table S12 | Sensitivity analysis across alternative Clonality Score formulations.**

Spearman correlation coefficients (ρ) between five alternative Clonality Score formulations and key immune variables. CS_original: primary Clonality Score (binder-weighted VAF / [neo_n_500 + 1]); CS_log: log-transformed Clonality Score; Raw_binder_weighted_VAF: unnormalised binder-weighted VAF; Mean_VAF_per_binder: average VAF across binder peptides; CS_purity_adjusted: Clonality Score divided by ABSOLUTE tumour purity. All formulations except the purity-adjusted variant reproduce significant negative correlations with immune variables, demonstrating robustness to the specific metric definition. The attenuation with purity adjustment is expected given the strong CS–purity correlation (ρ = 0.805) and is addressed by the ABSOLUTE Subclonal Genome Fraction validation (Supplementary Table S9).

| **Clonality Metric** | **ExhaustionScore rho** | **ExhaustionScore p** | **TIDE_dysfunction rho** | **TIDE_dysfunction p** | **CYT rho** | **CYT p** | **APM_Score rho** | **APM_Score p** | **IFNg_Score rho** | **IFNg_Score p** |
| --- | --- | --- | --- | --- | --- | --- | --- | --- | --- | --- |
| CS_original | -0.468 | 2e-28 | -0.533 | 6.71e-38 | -0.301 | 8.6e-12 | -0.367 | 2.43e-17 | -0.368 | 2.18e-17 |
| CS_log | -0.468 | 2e-28 | -0.533 | 6.71e-38 | -0.301 | 8.6e-12 | -0.367 | 2.43e-17 | -0.368 | 2.18e-17 |
| Raw_binder_weighted_VAF | -0.236 | 9.63e-08 | -0.304 | 4.03e-12 | -0.105 | 0.0194 | -0.181 | 4.67e-05 | -0.213 | 1.71e-06 |
| Mean_VAF_per_binder | -0.467 | 2.31e-28 | -0.533 | 8.01e-38 | -0.3 | 9.07e-12 | -0.367 | 2.58e-17 | -0.367 | 2.31e-17 |
| CS_purity_adjusted | -0.023 | 0.612 | 0.041 | 0.365 | -0.029 | 0.526 | 0.046 | 0.311 | -0.02 | 0.66 |
