# Supplementary material for: Inverse relationship between neoantigen clonality and T-cell activity reveals distinct immune phenotypes in HNSCC
Source: J Transl Med. 2026 Jun 3;24:731. doi: 10.1186/s12967-026-08371-z (PMC13235206; doi:10.1186/s12967-026-08371-z)
Supplement: Supplementary file 19 — Supplementary Material 19 [file 12967_2026_8371_MOESM19_ESM.docx]

**Supplementary Table S13 | Unsupervised clustering validation of four-phenotype classification.**

Results of unsupervised clustering analyses applied to z-standardised Clonality Score and TIDE dysfunction values. K-means clustering (k = 2–5) and Gaussian Mixture Models (GMM; k = 2–5) were evaluated. Silhouette scores assess cluster separation (higher is better). The Bayesian Information Criterion (BIC) favours k = 2, consistent with the primary hot/cold biological axis, while the Akaike Information Criterion (AIC; not shown) favours k = 4. The Adjusted Rand Index (ARI) quantifies concordance between unsupervised clusters and the median-split four-phenotype classification (ARI = 0 indicates random agreement; ARI = 1 indicates perfect concordance). K-means k = 4 achieves ARI = 0.496, indicating moderate-to-good concordance. Tertile-based stratification confirmed a monotonic gradient: CS tertile Low (mean TIDE dysfunction = 0.438, mean CYT = 13.4), Mid (0.057, 12.5), and High (−0.391, 6.3).

| **Method & k** | **Silhouette Score** | **Inertia** | **BIC** | **ARI vs median split** | **Notes** |
| --- | --- | --- | --- | --- | --- |
| K-means, k = 2 | 0.405 | 508.4 |  |  |  |
| K-means, k = 3 | 0.356 | 359.1 |  |  |  |
| K-means, k = 4 | 0.359 | 280.6 |  | 0.496 |  |
| K-means, k = 5 | 0.353 | 227.9 |  |  |  |
| GMM, k = 2 |  |  | 2691.7 |  |  |
| GMM, k = 3 |  |  | 2714.5 |  |  |
| GMM, k = 4 |  |  | 2736.7 | 0.402 |  |
| GMM, k = 5 |  |  | 2770.0 |  |  |
