# Supplementary material for: Inverse relationship between neoantigen clonality and T-cell activity reveals distinct immune phenotypes in HNSCC
Source: J Transl Med. 2026 Jun 3;24:731. doi: 10.1186/s12967-026-08371-z (PMC13235206; doi:10.1186/s12967-026-08371-z)
Supplement: Supplementary file 20 — Supplementary Material 20 [file 12967_2026_8371_MOESM20_ESM.docx]

**Supplementary Table S14 | Survival cohort composition and comparison of included versus excluded patients.**

Breakdown of the survival analysis cohort. Of 527 tumours in the full cohort, 457 had complete data for survival analysis (overall survival time > 0, Clonality Score, TIDE dysfunction score). Exclusions comprised patients with missing survival time (n = 45), missing Clonality Score due to absence of predicted neoantigen binders (n = 19), and missing TIDE dysfunction scores (n = 6). Comparison of included versus excluded patients revealed no significant differences in age, TMB, Clonality Score, or HPV status distribution, suggesting that missingness was non-informative.

| **Category** | **Value** | **Percentage / comparison** |
| --- | --- | --- |
| Total cohort | 527 | 100.0 |
| With vital status | 527 | 100.0 |
| With overall survival time > 0 | 482 | 91.5 |
| With Clonality Score | 505 | 95.8 |
| With TIDE dysfunction score | 520 | 98.7 |
| Complete cases for survival analysis | 457 | 86.7 |
|  |  |  |
| **Exclusion reasons** | n excluded | % |
| Missing survival time | 45 | 8.5 |
| Missing Clonality Score (no neoantigen binders) | 19 | 3.6 |
| Missing TIDE dysfunction | 6 | 1.1 |
|  |  |  |
| **Comparison: included vs excluded** | Included (n=457) | Excluded (n=70) |
| Median age at diagnosis | 61 | 59 |
| Mann-Whitney P | 0.237 |  |
| Median TMB (mut/Mb) | 2.75 | 2.78 |
| Mann-Whitney P | 0.998 |  |
| Median Clonality Score | 0.23 | 0.22 |
| Mann-Whitney P | 0.588 |  |
| HPV+ frequency | 66.3% | 62.9% |
| Chi-squared P | 0.702 |  |
