# Supplementary material for: Inverse relationship between neoantigen clonality and T-cell activity reveals distinct immune phenotypes in HNSCC
Source: J Transl Med. 2026 Jun 3;24:731. doi: 10.1186/s12967-026-08371-z (PMC13235206; doi:10.1186/s12967-026-08371-z)
Supplement: Supplementary file 21 — Supplementary Material 21 [file 12967_2026_8371_MOESM21_ESM.docx]

**Supplementary Table S15 | Validation of hot/cold immune classification using alternative immune scores.**

Spearman correlation coefficients (rho) between the Clonality Score and eleven alternative immune-context measures derived from bulk RNA-seq, single-cell-marker genes, and CIBERSORT deconvolution. Hot/cold classifications based on each alternative score were compared against the primary TIDE-dysfunction-based classification using percent concordance and Cohen’s kappa. The Clonality Score showed significant negative correlations with 10/11 alternative metrics. Hot/cold classifications were highly concordant across transcriptomic immune scores (concordance 67.7–79.8%, Cohen’s kappa 0.353–0.597), supporting the robustness of the four-phenotype framework to the choice of immune metric. The Mann–Whitney P-value compares Clonality Score distributions between hot and cold tumours classified by each alternative score.

| **Immune Score** | **n** | **CS rho** | **CS p-value** | **Hot/Cold Concordance with TIDE (%)** | **Cohen's kappa** | **CS difference Hot vs Cold P** |
| --- | --- | --- | --- | --- | --- | --- |
| CD8A (gene) | 498 | -0.078 | 0.0808 | 61.6 | 0.233 | 0.125 |
| CD3D expression | 498 | -0.38 | 1.55e-18 | 78.1 | 0.562 | 3.81e-12 |
| GZMB expression | 498 | -0.306 | 2.73e-12 | 69.7 | 0.394 | 8.51e-10 |
| GNLY expression | 498 | -0.243 | 3.84e-08 | 67.7 | 0.353 | 7.81e-07 |
| NKG7 expression | 498 | -0.336 | 1.35e-14 | 73.3 | 0.466 | 1.97e-06 |
| CYT (Rooney GZMA*PRF1) | 495 | -0.301 | 8.6e-12 | 69.7 | 0.394 | 1.07e-08 |
| ESTIMATE-style Immune Score | 498 | -0.389 | 1.75e-19 | 74.1 | 0.482 | 3.22e-13 |
| IFN-gamma Score | 498 | -0.368 | 2.18e-17 | 70.9 | 0.418 | 5.16e-12 |
| CIBERSORT CD8 T cells | 498 | -0.078 | 0.0808 | 61.6 | 0.233 | 0.125 |
| sig_LIexpression_score (Wolf) | 491 | -0.455 | 1.87e-26 | 79.8 | 0.597 | 3.62e-15 |
| sig_ICR_SCORE | 491 | -0.385 | 9.47e-19 | 74.9 | 0.499 | 5.3e-12 |
