# Supplementary material for: Inverse relationship between neoantigen clonality and T-cell activity reveals distinct immune phenotypes in HNSCC
Source: J Transl Med. 2026 Jun 3;24:731. doi: 10.1186/s12967-026-08371-z (PMC13235206; doi:10.1186/s12967-026-08371-z)
Supplement: Supplementary file 22 — Supplementary Material 22 [file 12967_2026_8371_MOESM22_ESM.docx]

**Supplementary Table S16 | Pairwise log-rank tests between immune phenotype groups.**

Pairwise log-rank tests for overall survival between all six pairs of the four immune phenotypes, with Benjamini–Hochberg false discovery rate (FDR) and Bonferroni corrections for multiple comparisons. The global multivariate log-rank test was significant (chi-squared = 14.48, P = 0.0023, n = 476). Three pairwise contrasts remained significant after FDR adjustment (P_FDR < 0.05): Hot/High Clonality vs. Cold/High Clonality, Hot/High Clonality vs. Cold/Low Clonality, and Hot/Low Clonality vs. Hot/High Clonality. The survival advantage is therefore concentrated in the Hot/High Clonality group, consistent with the interaction model demonstrating that clonality confers prognostic benefit only in immune-infiltrated tumours.

| **Group 1** | **Group 2** | **n1** | **n2** | **events1** | **events2** | **chi-squared** | **P (raw)** | **P (FDR-BH)** | **P (Bonferroni)** | **Significant (FDR < 0.05)** |
| --- | --- | --- | --- | --- | --- | --- | --- | --- | --- | --- |
| Hot/Low Clonality | Hot/High Clonality | 161 | 78 | 74 | 26 | 5.23 | 0.0221 | 0.0443 | 0.1328 | Yes |
| Hot/Low Clonality | Cold/Low Clonality | 161 | 84 | 74 | 38 | 1.12 | 0.2906 | 0.3487 | 1.0000 | No |
| Hot/Low Clonality | Cold/High Clonality | 161 | 153 | 74 | 82 | 3.42 | 0.0642 | 0.0963 | 0.3853 | No |
| Hot/High Clonality | Cold/Low Clonality | 78 | 84 | 26 | 38 | 9.81 | 0.0017 | 0.0052 | 0.0104 | Yes |
| Hot/High Clonality | Cold/High Clonality | 78 | 153 | 26 | 82 | 13.61 | 0.0002 | 0.0013 | 0.0013 | Yes |
| Cold/Low Clonality | Cold/High Clonality | 84 | 153 | 38 | 82 | 0.46 | 0.4972 | 0.4972 | 1.0000 | No |
