# Supplementary material for: Inverse relationship between neoantigen clonality and T-cell activity reveals distinct immune phenotypes in HNSCC
Source: J Transl Med. 2026 Jun 3;24:731. doi: 10.1186/s12967-026-08371-z (PMC13235206; doi:10.1186/s12967-026-08371-z)
Supplement: Supplementary file 23 — Supplementary Material 23 [file 12967_2026_8371_MOESM23_ESM.docx]

**Supplementary Table S17 | Multivariable Cox regression with four-phenotype categorical predictor.**

Hazard ratios (HR) with 95% confidence intervals from a multivariable Cox proportional hazards model treating the four-phenotype variable as a single categorical predictor with Hot/Low Clonality as the reference category. The model was adjusted for age at diagnosis (z-standardised), advanced clinical stage (III–IV vs. I–II), and HPV status (n = 405). Hot/High Clonality tumours showed significantly improved survival relative to the Hot/Low Clonality reference (HR = 0.54, P = 0.026), whereas Cold/High Clonality and Cold/Low Clonality did not differ significantly from the reference. HPV status and advanced stage were not independently prognostic in this model. This categorical analysis confirms that the prognostic benefit of the Hot/High Clonality phenotype is independent of HPV status and other clinical covariates.

| **Variable** | **HR** | **95% CI lower** | **95% CI upper** | **p-value** |
| --- | --- | --- | --- | --- |
| Hot/Low Clonality (reference) | 1.00 (ref) | - | - | - |
| Age_z | 1.31 | 1.10 | 1.56 | 0.0023 |
| AdvancedStage | 1.36 | 0.93 | 2.00 | 0.1116 |
| HPV_positive | 1.10 | 0.78 | 1.55 | 0.5709 |
| Cold/High Clonality | 1.40 | 0.96 | 2.05 | 0.0799 |
| Cold/Low Clonality | 1.31 | 0.82 | 2.08 | 0.2540 |
| Hot/High Clonality | 0.54 | 0.31 | 0.93 | 0.0258 |
